# High-Fidelity Simulation with Transvaginal Ultrasound in the Emergency Department

**DOI:** 10.21980/J8606Q

**Published:** 2024-07-31

**Authors:** Levi Filler, Katrina Lettang

**Affiliations:** * Creighton University School of Medicine Phoenix Program, Valleywise Health Medical Center, Department of Emergency Medicine, Phoenix, AZ

## Abstract

**Audience:**

Intern and junior emergency medicine residents.

**Introduction:**

Abdominal pain and vaginal bleeding in the first trimester of pregnancy are common presentations to the emergency department (ED).^1^ Formal transvaginal ultrasound (TVUS) is considered the test of choice for evaluation of first trimester pregnancy due to its high sensitivity and specificity for identifying intrauterine and ectopic pregnancies.^1^ Additionally, TVUS can evaluate for various uterine and ovarian pathology as well as identify other non-gynecologic conditions and is within the scope of practice for the emergency physician.^2^ Given the emergent and time sensitive nature of certain obstetric and gynecologic conditions, formal transvaginal ultrasound imaging may not be feasible. A rapid assessment with transvaginal point-of-care ultrasound (TVPOCUS) can be utilized by emergency medicine physicians (EMP) to confirm intrauterine pregnancies (IUP) and identify any associated complications. There are multiple advantages to TVPOCUS including reduced cost and length of stay, patient satisfaction, and improved resource utilization.^1^,^3^ Additionally, multiple studies demonstrate that EMPs can learn this skill and perform TVPOCUS accurately and safely.^1^,^3^ Developing the skills and comfort with TVPOCUS in a simulation setting during residency is beneficial and can have important implications in future practice.

**Educational Objectives:**

By the end of the session, learners should be able to 1) recognize the clinical indications for transvaginal ultrasound in the ED, 2) practice the insertion, orientation, and sweeping motions used to perform a TVPOCUS study, 3) interpret transvaginal ultrasound images showing an IUP or alternative pathologies, and 4) understand proper barrier, disinfection, and storage techniques for endocavitary probes.

**Educational Methods:**

This session included three high-fidelity simulation cases that allowed participants to utilize TVPOCUS in a safe and conducive environment. There was a total of 32 emergency medicine (EM) residents who participated. The simulation sessions were divided into two separate rooms and included four learners for each session that actively managed the patient, for a total of 12 active participants. The 20 remaining residents were observers. Participants learned evidence-based indications, performance, and interpretation of transvaginal ultrasound. Three cases were reviewed and included IUP, ruptured ectopic pregnancy with hemorrhagic shock, and appendicitis in pregnancy. The cases were followed by a debriefing session and discussion regarding the evidence behind bedside transvaginal ultrasound, its incorporation into EM workflow, and practice-based learning.

**Research Methods:**

The educational content and efficacy were evaluated by oral feedback in a debriefing session after the workshop. Additionally, pre-simulation and post-simulation surveys were sent to participants to assess prior ultrasound experience and confidence on the indications, performance, and interpretation of transvaginal ultrasound. Responses were collected using a Likert scale of 1 to 5, with 1 being “not at all confident” and 5 being “very confident.”

**Results:**

Ten learners responded to the survey consisting of EM residents in a three-year EM residency program. Prior to the workshop, the median reported confidence level across all questions was “1- not at all confident” for the PGY-1 class, and “3-neutral” for the PGY-2 and PGY-3 classes. Following the workshop, all median scores across all classes were “4-confident,” demonstrating an increase in confidence scoring across all measurements and participants. Incorporating transvaginal ultrasound into clinical workflow demonstrated the largest increase in confidence score (median 1.5 to 4), followed by insertion/orientation of the endocavitary probe (median 2.5 to 5).

**Discussion:**

This high-fidelity simulation familiarized learners with transvaginal ultrasound and how it can be appropriately utilized for a variety of high-yield clinical scenarios that present regularly to the ED. Given the variation in ultrasound training among residency programs, and the lack of specific simulation content addressing this modality, it is important to implement scenarios that improve learner comfort with TVPOCUS. Overall, this workshop resulted in an increase in confidence scores of participants in the indication, performance, and interpretation of TVPOCUS in the ED.

**Topics:**

Transvaginal ultrasound, POCUS, intrauterine pregnancy, ectopic pregnancy, hemorrhagic shock, appendicitis in pregnancy, abdominal pain, emergency medicine.

## USER GUIDE

[Table t1-9-3-s65]

**Table t1-9-3-s65:** 

List of Resources:	
Abstract	65
User Guide	67
Case 1: Instructor Materials	71
Case 1: Operator Materials	82
Case 1: Debriefing and Evaluation Pearls	84
Case 1: Simulation Assessment	86
Case 2: Instructor Materials	91
Case 2: Operator Materials	102
Case 2: Debriefing and Evaluation Pearls	105
Case 2: Simulation Assessment	107
Case 3: Instructor Materials	112
Case 3: Operator Materials	124
Case 3: Debriefing and Evaluation Pearls	128
Case 3: Simulation Assessment	131

**Learner Audience:**

Intern and junior emergency medicine residents

**Time Required for Implementation:**

Instructor Preparation: 15–20 minutes

Time for case: 20–30 minutes

Time for debriefing: 20–30 minutes

**Recommended Number of Learners per Instructor:**

3–4

**Topics:**

Transvaginal ultrasound, POCUS, intrauterine pregnancy, ectopic pregnancy, hemorrhagic shock, appendicitis in pregnancy, abdominal pain, emergency medicine.

**Objectives:**

By the end of this simulation session, the learner will be able to:

1. Recognize the clinical indications for transvaginal ultrasound in the ED.
2. Practice the insertion, orientation, and sweeping motions used to perform a TVPOCUS study.
3. Interpret transvaginal ultrasound images showing an intrauterine pregnancy (IUP) or alternative pathologies.
4. Understand proper barrier, disinfection, and storage techniques for endocavitary probes. (This was institution specific, limiting generalizability.)

### Linked objectives and methods

Obstetric and gynecologic emergencies are common presentations to the ED, and expeditious evaluation and diagnosis are essential. In this simulation, learners are presented with three cases. The first case is of a young female with lower abdominal pain and vaginal spotting in the setting of missed menses. Once pregnancy is suspected, learners are presented with transabdominal ultrasound (TAUS) imaging that appears indeterminate for IUP (objective #1). Learners will then need to discuss, set up and perform a proper transvaginal ultrasound study (objective #2) and properly interpret normal anatomy and identify an IUP (objective #3). In the second case, learners are presented with a case of right lower quadrant pain in pregnancy and will need to recognize secondary features of appendicitis as a concurrent diagnosis in the setting of a visualized IUP (objective #3). In the final case, residents are given a case of an unstable patient who has a ruptured ectopic pregnancy and an absent IUP on TVPOCUS (objective #3). At the end of the sessions, learners will need to discuss proper barrier, disinfection, and storage techniques of the endocavitary probe based on their specific institution’s policies (objective #4).

### Recommended pre-reading for instructor

- Tahapary M, Cornelis A, Peersman B, Van den Bosch T. Diagnosis of appendicitis by transvaginal ultrasound examination. *Australas J Ultrasound Med.* 2021;24(2):102–105. At: doi:https://doi.org/10.1002/ajum.12235
- Shih CH. Effect of emergency physician-performed pelvic sonography on length of stay in the emergency department. *Ann Emerg Med.* 1997;29(3):348–352. At: doi:https://doi.org/10.1016/s0196-0644(97)70346-9
- Van Schaik GW, Van Schaik KD, Murphy MC. Point-of-care ultrasonography (pocus) in a community emergency department: an analysis of decision making and Cost Savings Associated With POCUS. *J Ultrasound Med.* 2018;38(8):2133–2140. At: doi:https://doi.org/10.1002/jum.14910
- Dawson M, Mallin M. *Introduction to Bedside Ultrasound*. Vol 1. Emergency Ultrasound Solutions. Accessed June 26, 2024. At: https://www.scribd.com/document/533844537/Introduction-to-Bedside-Ultrasound-Volume-1-Matt-and-Mike

### Results and tips for successful implementation

This simulation session was conducted with a total of 32 EM residents, with 12 PGY-1–3 residents actively managing the case, and 20 observers. One actor served as a nurse during the simulation. Roles were assigned among residents prior to the start of the cases. Proper equipment is paramount for a successful simulation and may prove to be a barrier if availability is limited. The endocavitary probe was used during all the cases and shared between both simulation rooms. A second probe would allow the simulation to run with minimal interruption if two rooms are utilized simultaneously. Ideally, a female TVUS mannequin would be used for TVPOCUS performance and image acquisition. However, these models may be cost prohibitive or difficult to obtain. If none are available, a female high fidelity simulation mannequin with a vaginal canal would be necessary to allow for endocavitary probe insertion. In this case, the probe would not need to be plugged into an ultrasound machine, and visual or video prompts would need to be provided. Endocavitary probe covers are required to promote the importance of transvaginal ultrasound safety, and prompts discussion of institution-specific infection control policies. Furthermore, a simulated moulage kit, obscured under the sheet covering the mannequin, can be used to simulate severe vaginal bleeding in the setting of ectopic pregnancy. This provides learners with further education surrounding the importance of a prompt and thorough physical examination at the bedside, especially with an unstable patient. In our simulation workshop, we did not have access to a mannequin compatible with TVUS. However, participants still found that holding the endocavitary probe, applying the probe cover, inserting the probe into the simulation mannequin, and practicing the sweeping motion to be a valuable experience that familiarized them with a sensitive procedure in a controlled and safe setting.

For simulation personnel, it was easier to execute case #3 first because it enabled them to apply the moulage prior to the workshop to save time. The patient set-up for cases #1 and #2 are very similar and we do not recommend a particular order. Note that the cases are ordered by level of acuity with #1 being the lowest and #3 being the highest. Some participants found ending with case #1 to be anticlimactic after working through cases #2 and #3. Therefore, the preferred order of cases for efficiency and rhythm may be #3, #1, and then #2.

A faculty member trained in ultrasound, preferably fellowship trained, is recommended to help run the simulation sessions and provide proper instruction and guidance, especially if learners have never used an endocavitary probe before. Taking the time to address the importance of obtaining a chaperone as well as proper verbal consent is recommended either during the simulation or at debrief to reinforce proper practices. A debrief session was held following each simulation. A survey was sent to all 32 participating residents via surveymonkey.com both before and after the simulation workshop. The survey assessed knowledge regarding TVPOCUS across three topics: clinical integration, performance, and interpretation. Responses were collected using a Likert scale from 1 to 5, with 1 being “not at all confident” and 5 being “very confident.” The survey collected responses to the following questions:

Demographics:

1. What is your PGY year?
2. Approximately how many clinical ultrasound studies have you performed in your life, including medical school?

Confidence:

1. How confident are you with the clinical indications for using transvaginal ultrasound?
2. How confident are you with the incorporation of transvaginal ultrasound into your patient workflow?
3. How confident are you with the storage, barrier protection, and cleaning processes of the endocavitary transducer?
4. How confident are you with the insertion and orientation of the endocavitary transducer?
5. How confident are you with the identification of normal anatomy on a transvaginal ultrasound study?
6. How confident are you with the identifications of an IUP on a transvaginal ultrasound study?
7. How confident are you with the identification of pelvic pathology on a transvaginal ultrasound study?

**Figure 2:**
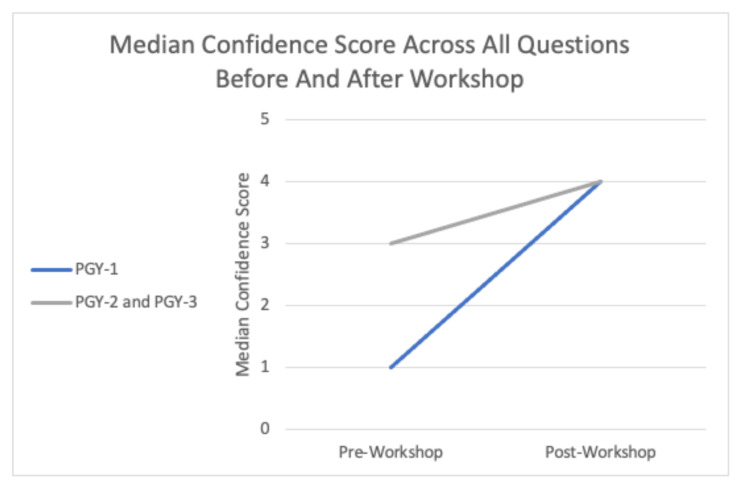
Mean confidence scores across all questions pre- and post-simulation

The workshop demonstrated a significant increase in participant confidence across all three topics (Figure 1). The largest improvement in median scores was observed in the PGY-1 class (Figure 2). The largest change in median confidence score was noted pre- and post-workshop in the incorporation of transvaginal ultrasound into patient workflow (pre-workshop median 1.5 to post-workshop median 4) and the insertion and orientation of the endocavitary probe (pre-workshop median of 2.5 to post-workshop median of 5).

## Supplementary Information





## Figures and Tables

**Table t2-9-3-s65:** 

#1	Complete blood count (CBC)
#2	Comprehensive metabolic panel (CMP)
#3	ABO/Rh blood groups
#4	Beta human chorionic gonadotropin (B-hcg)
#5	Urinalysis (UA)
#6	Transabdominal ultrasound
#7	Transvaginal ultrasound

**Table t3-9-3-s65:** 

Critical Action	Met	Not Met	Comments
Obtain appropriate history of present illness and medical history from the patient			
Perform focused physical exam on the patient			
Request appropriate diagnostic tests on the patient including pregnancy test and bedside ultrasound imaging			
Correctly apply endocavitary probe barrier and insert with appropriate probe orientation. Demonstrate sweeping motion in transverse and sagittal planes			
Correctly interpret a TVUS IUP image			
Educate patient on diagnosis and disposition			

**Table t4-9-3-s65:** 

**Vitals:**	Heart Rate (HR) 92	Blood Pressure (BP) 112/65	Respiratory Rate (RR) 14
	Temperature (T) 37.4°C	Oxygen Saturation (O_2_Sat) 99% on room air	

**Table t5-9-3-s65:** 

*Complete blood count (CBC)*	
White blood count (WBC)	10.0 × 1000/mm^3^
Hemoglobin (Hgb)	9.8 g/dL
Hematocrit (HCT)	30.5%
Red blood cells (RBC)	5.5 × 1000/mm^3^
Mean Corpuscular Volume (MCV)	67 fL
Platelet (Plt)	250 × 1000/mm^3^
Neutrophils	3.5 × 1000/mm^3^
Lymphocytes	2.5 × 1000/mm^3^

**Table t6-9-3-s65:** 

*ABO/Rh*	O positive/Rh+
*B-hcg*	4,500 mlU/mL

**Table t7-9-3-s65:** 

*Comprehensive metabolic panel (CMP)*	
Sodium	135 mEq/L
Potassium	3.5 mEq/L
Blood Urea Nitrogen (BUN)	20 mg/dL
Creatinine (Cr)	0.6 mg/dL
Calcium	8.5 mg/dL
Chloride	100 mEq/L
Bicarbonate	24 mEq/L
Aspartate aminotransferase (AST)	35 U/L
Alanine aminotransferase (ALT)	45 U/L
Alkaline Phosphatase	55 U/L
Bilirubin	<1 mg/dL
Albumin	4.1 g/dL

**Table t8-9-3-s65:** 

*Urinalysis (UA)*	
Spec gravity:	1.010
Appearance	Clear
Color	Yellow
Glucose	Negative
Bilirubin	Negative
Ketones	Negative
Blood	None
pH	7.0
Protein	Negative
Urobilinogen	Negative
Leukocyte esterase	Negative
Nitrites	Negative
Bacteria	Negative
White blood cells	0–5 WBCs/HPF
Red blood cells	0–5 RBCs/HPF
Squamous cells	0–5 cells/HPF

**Figure f3-9-3-s65:**
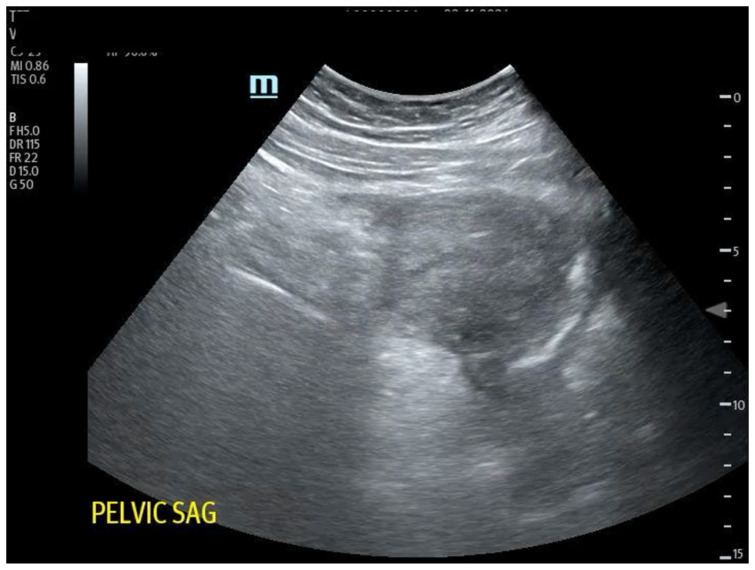
Stimulus 1

**Figure f4-9-3-s65:**
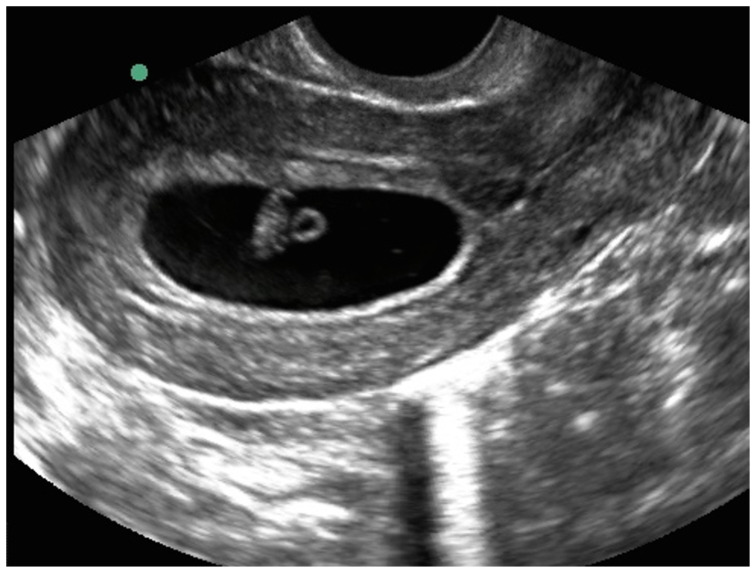
Stimulus 2

**Figure f5-9-3-s65:**
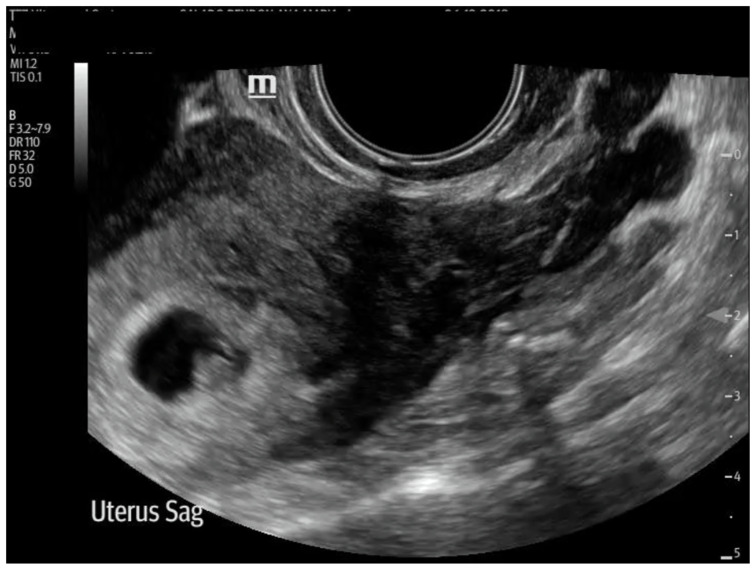
Stimulus 3

**Table t9-9-3-s65:** SIMULATION EVENTS TABLE Case 1 Abdominal Pain in Pregnancy:

Minute (state)	Participant action/trigger	Patient status (simulator response) & operator prompts	Monitor display (vital signs)
0:00 (Baseline)	Patient in a room, appears comfortable and in no distress. Participant should obtain history as well as physical exam during this step. The participant may opt to perform a pelvic exam here. Placing the patient on the monitor is not a required step but may happen here. While the participant is doing the examination, you may proceed to step two.	Patient discloses history but appears somewhat nervous. She has lower abdominal exam tenderness, but it is vague and across the whole abdomen	T 36.5° C HR 92 BP 112/65 RR 14 O2 99%
5:00	Participant should perform a trans abdominal ultrasound. The ultrasound images will be indeterminant and the participant will not be able to see an IUP. The participant should update the patient regarding these findings. Participant should opt to perform a transvaginal ultrasound to determine if there is an IUP. Participant should obtain verbal consent from the patient and walk through the steps of a transvaginal ultrasound.	Nurse informs participant that the ultrasound technician got in a car accident and will not be able to come in, but that there is a bedside ultrasound machine available. Display STIMULUS 1 image. Patient will be confused that the TAUS is indeterminant and ask what that means and what the next steps are. If the participant does not opt to perform a transvaginal ultrasound, they may be prompted by the nurse that the probe is available. If the patient does not receive information on the steps of a transvaginal ultrasound, the patient should ask what that is and what the steps are.	T 36.5° C HR 92 BP 112/65 RR 14 O2 99%
7:00	Participant performs a transvaginal ultrasound. The participant should interpret an IUP. If the participant does not interpret an IUP, they may confer with a colleague or call the radiologist.	Display STIMULUS 2 and 3	T 36.5° C HR 92 BP 112/65 RR 14 O2 99%
9.00 (Case Completion)	Participant should discuss next steps for the patient including OB follow up and return precautions.	Patient will ask questions about follow up and return precautions if not prompted.	T 36.5° C HR 92 BP 112/65 RR 14 O2 99%

**Figure f6-9-3-s65:**
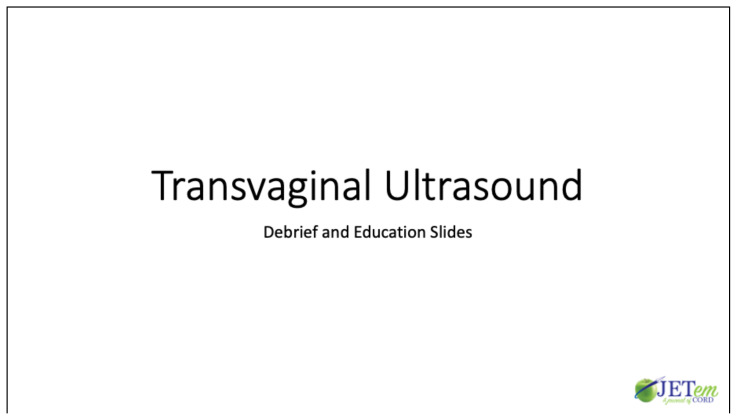
Please see associated PowerPoint file

**Table t10-9-3-s65:** 

**Critical Actions:** Obtain an appropriate HPI and medical history from the patient. Perform a focused physical exam. Request the appropriate diagnostic tests on the patient, including a pregnancy test and bedside ultrasound imaging. Correctly apply the endocavitary probe barrier and insert it with appropriate probe orientation. Demonstrate sweeping motion in transverse and sagittal planes. Correctly interpret a TVUS IUP image. Educate the patient on diagnosis and disposition.	0:00

**Table t11-9-3-s65:** 

	Milestone	Did not achieve level 1	Level 1	Level 2	Level 3
1	**Emergency Stabilization (PC1)**	□ Did not achieve Level 1	□ Recognizes abnormal vital signs	□ Recognizes an unstable patient, requiring intervention Performs primary assessment Discerns data to formulate a diagnostic impression/plan	□ Manages and prioritizes critical actions in a critically ill patient Reassesses after implementing a stabilizing intervention
2	**Performance of focused history and physical (PC2)**	□ Did not achieve Level 1	□ Performs a reliable, comprehensive history and physical exam	□ Performs and communicates a focused history and physical exam based on chief complaint and urgent issues	□ Prioritizes essential components of history and physical exam given dynamic circumstances
3	**Diagnostic studies (PC3)**	□ Did not achieve Level 1	□ Determines the necessity of diagnostic studies	□ Orders appropriate diagnostic studies. Performs appropriate bedside diagnostic studies/procedures	□ Prioritizes essential testing Interprets results of diagnostic studies Reviews risks, benefits, contraindications, and alternatives to a diagnostic study or procedure
4	**Diagnosis (PC4)**	□ Did not achieve Level 1	□ Considers a list of potential diagnoses	□ Considers an appropriate list of potential diagnosis May or may not make correct diagnosis	□ Makes the appropriate diagnosis Considers other potential diagnoses, avoiding premature closure
5	**Pharmacotherapy (PC5)**	□ Did not achieve Level 1	□ Asks patient for drug allergies	□ Selects an medication for therapeutic intervention, consider potential adverse effects	□ Selects the most appropriate medication and understands mechanism of action, effect, and potential side effects Considers and recognizes drug-drug interactions
6	**Observation and reassessment (PC6)**	□ Did not achieve Level 1	□ Reevaluates patient at least one time during case	□ Reevaluates patient after most therapeutic interventions	□ Consistently evaluates the effectiveness of therapies at appropriate intervals
7	**Disposition (PC7)**	□ Did not achieve Level 1	□ Appropriately selects whether to admit or discharge the patient	□ Appropriately selects whether to admit or discharge Involves the expertise of some of the appropriate specialists	□ Educates the patient appropriately about their disposition Assigns patient to an appropriate level of care (ICU/Tele/Floor) Involves expertise of all appropriate specialists
9	**General Approach to Procedures (PC9)**	□ Did not achieve Level 1	□ Identifies pertinent anatomy and physiology for a procedure Uses appropriate Universal Precautions	□ Obtains informed consent Knows indications, contraindications, anatomic landmarks, equipment, anesthetic and procedural technique, and potential complications for common ED procedures	□ Determines a back-up strategy if initial attempts are unsuccessful Correctly interprets results of diagnostic procedure
20	**Professional Values (PROF1)**	□ Did not achieve Level 1	□ Demonstrates caring, honest behavior	□ Exhibits compassion, respect, sensitivity and responsiveness	□ Develops alternative care plans when patients’ personal beliefs and decisions preclude standard care
22	**Patient centered communication (ICS1)**	□ Did not achieve level 1	□ Establishes rapport and demonstrates empathy to patient (and family) Listens effectively	□ Elicits patient’s reason for seeking health care	□ Manages patient expectations in a manner that minimizes potential for stress, conflict, and misunderstanding. Effectively communicates with vulnerable populations, (at risk patients and families)
23	**Team management (ICS2)**	□ Did not achieve level 1	□ Recognizes other members of the patient care team during case (nurse, techs)	□ Communicates pertinent information to other healthcare colleagues	□ Communicates a clear, succinct, and appropriate handoff with specialists and other colleagues Communicates effectively with ancillary staff

**Table t12-9-3-s65:** 

#1	Complete blood count (CBC)
#2	Comprehensive metabolic panel (CMP)
#3	ABO/Rh blood groups
#4	Beta human chorionic gonadotropin (B-hcg)
#5	Urinalysis (UA)
#6	Prothrombin time/international normalized ratio (PT/INR)
#7	Transabdominal ultrasound
#8	Transvaginal ultrasound image
#9	Transvaginal ultrasound image with fetal heart tone measurement
#10	MRI image of the abdomen

**Table t13-9-3-s65:** 

Critical Action	Met	Not Met	Comments
Obtain appropriate history of present illness and medical history from the patient.			
Perform focused physical exam on the patient.			
Request appropriate diagnostic tests on the patient including serologic tests, acquisition of fetal heart tones, and ultrasound.			
Address patient’s symptoms with medication.			
Correctly apply endocavitary probe barrier and insert with appropriate probe orientation. Demonstrate sweeping motion in transverse and sagittal planes.			
Correctly interpret a TVUS IUP image and fetal heart tones using M-mode ultrasonography			
Recognize appendicitis as potential etiology to patient’s symptoms.			
Obtain correct diagnostic test for diagnosis of appendicitis in pregnancy.			
Counsel patient on diagnosis and management of appendicitis in pregnancy.			
Consult general surgery.			

**Table t14-9-3-s65:** 

**Vitals:**	Heart Rate (HR) 110	Blood Pressure (BP) 104/62	Respiratory Rate (RR) 18
	Temperature (T) 36.5°C	Oxygen Saturation (O_2_Sat) 99% on room air	

**Table t15-9-3-s65:** 

*Complete blood count (CBC)*	
White blood count (WBC)	15.0 × 1000/mm^3^
Hemoglobin (Hgb)	12.8 g/dL
Hematocrit (HCT)	33.5%
Red blood cells (RBC)	5.5 × 1000/mm^3^
Mean Corpuscular Volume (MCV)	88 fL
Platelet (Plt)	250 × 1000/mm^3^
Neutrophils	3.5 × 1000/mm^3^
Lymphocytes	2.5 × 1000/mm^3^

**Table t16-9-3-s65:** 

*ABO/Rh*	O positive/Rh+
*B-hcg*	52,250 mlU/mL

**Table t17-9-3-s65:** 

*PT*	13 seconds
*INR*	1.0

**Table t18-9-3-s65:** 

*Comprehensive metabolic panel (CMP)*	
Sodium	135 mEq/L
Potassium	3.5 mEq/L
Blood Urea Nitrogen (BUN)	28 mg/dL
Creatinine (Cr)	0.87 mg/dL
Calcium	8.5 mg/dL
Chloride	100 mEq/L
Bicarbonate	24 mEq/L
Aspartate aminotransferase (AST)	35 U/L
Alanine aminotransferase (ALT)	45 U/L
Alkaline Phosphatase	55 U/L
Bilirubin	<1 mg/dL
Albumin	4.1 g/dL

**Table t19-9-3-s65:** 

*Urinalysis (UA)*	
Spec gravity:	1.020
Appearance	Cloudy
Color	Yellow
Glucose	Negative
Bilirubin	Negative
Ketones	Negative
Blood	None
pH	7.0
Protein	Negative
Urobilinogen	Negative
Leukocyte esterase	Large
Nitrites	Negative
Bacteria	Negative
White blood cells	10–20 WBCs/HPF
Red blood cells	0–5 RBCs/HPF
Squamous cells	0–5 cells/HPF

**Figure f7-9-3-s65:**
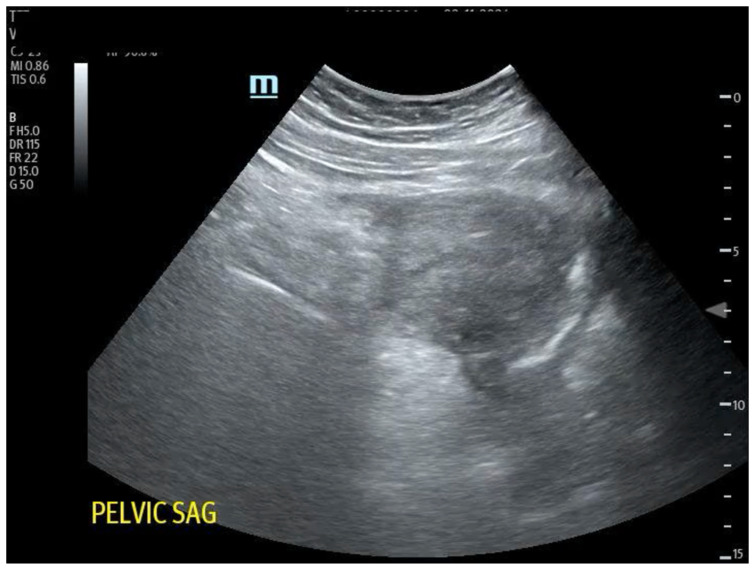
Stimulus 1

**Figure f8-9-3-s65:**
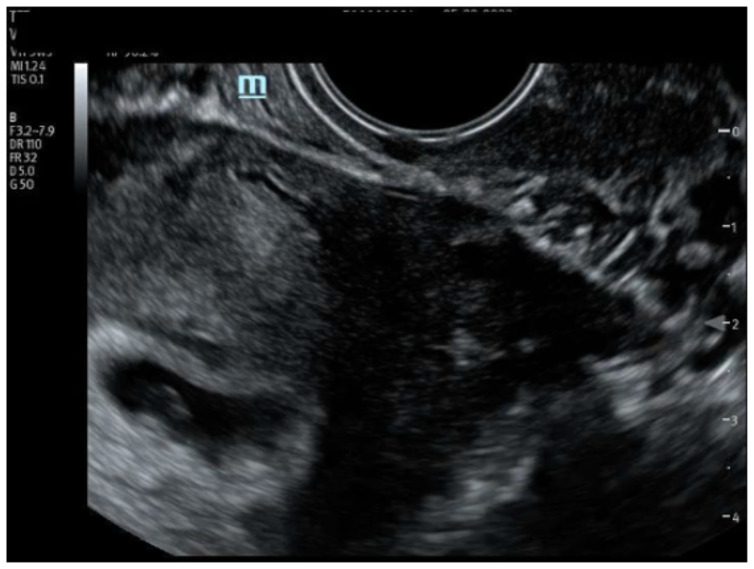
Stimulus 4

**Figure f9-9-3-s65:**
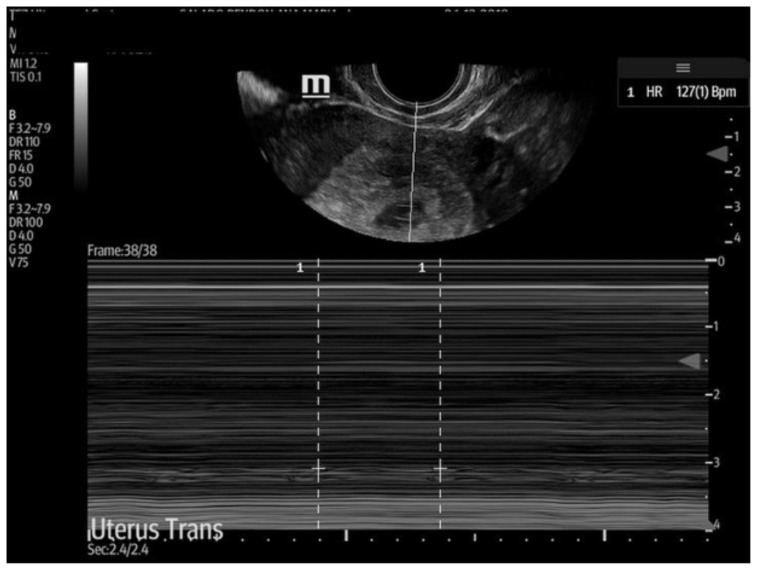
Stimulus 5

**Table t20-9-3-s65:** SIMULATION EVENTS TABLE Case 2 Right Lower Quadrant Abdominal Pain in Pregnancy:

Minute (State)	Participant Action/Trigger	Patient Status (Simulator Response) & Operator Prompts	Monitor Display (Vital Signs)
0:00 (Baseline)	The patient on arrival appears uncomfortable. The participant should place the patient on the monitor, obtain IV access, and obtain a history as well as conduct a physical exam. The participant may also obtain a pelvic exam here or defer until later.	Patient discloses history but is somewhat uncomfortable throughout history and examination. She has notable tenderness on the right side of the abdomen.	HR 110 BP 104/62 RR 18 T 36.5° C O_2_Sat 99%
2:00	The participant should request labs and provide pain medication to the patient. The participant may opt to obtain a bedside ultrasound at this point for FHTs. The participant may perform a pelvic examination.	Patient feels significant relief with pain medication. Bedside ultrasound demonstrates an IUP but there are no clear fetal heart tones. STIMULUS 1. Pelvic examination demonstrates no abnormalities (no bleeding, closed os, no CMT or discharge).	HR 110 (105 if pain medication is given) BP 104/62 RR 18 T 36.5° C O_2_Sat 99%
6:00	With no identifiable fetal heart tones, the participant may opt to obtain a TVUS.	The ultrasound tech is busy in another ultrasound study but will be available in 60 minutes. The nurse should prompt the participant to perform a TVUS.	HR 110 (105 if pain medication is given) BP 104/62 RR 18 T 36.5° C O_2_Sat 99%
8:00	The participant will perform a TVUS. The participant should counsel the patient on the steps of a TVUS and obtain verbal consent. The participant should interpret the TVUS and update the patient on their interpretation.	STIMULUS 4 AND 5 TVUS demonstrates +FHTs (HR 127) with trace pelvic free fluid. After interpretation of the TVUS, the participant should receive laboratory results and interpret them.	HR 110 (105 if pain medication is given) BP 104/62 RR 18 T 36.5° C O_2_Sat 99%
10:00	The participant should interpret laboratory results. Given trace pelvic free fluid on bedside ultrasound, the participant should obtain a formal lower abdominal ultrasound.	Ultrasound tech is now available and able to obtain an abdominal ultrasound. If requested, this demonstrates findings equivocal for an acute appendicitis.	HR 110 (105 if pain medication is given) BP 104/62 RR 18 T 36.5° C O_2_Sat 99%
13:00	Participant must discuss results with the patient and next steps. The participant should be concerned for appendicitis. The participant may opt for MRI or shared decision making for CT abdomen/pelvis.	MRI and CT both demonstrate acute appendicitis.	HR 110 (105 if pain medication is given) BP 104/62 RR 18 T 36.5° C O_2_Sat 99%
(Case Completion)	Participant should discuss results with the patient and initiate disposition and management including antibiotics, IVF, NPO status, and general surgery/OBGYN consultation.	Consultants may give antibiotic recommendations if they are not started yet. Consultants agree with operative management and will come to see the patient.	HR 110 (105 if pain medication is given) BP 104/62 RR 18 T 36.5° C O_2_Sat 99%

**Figure f10-9-3-s65:**
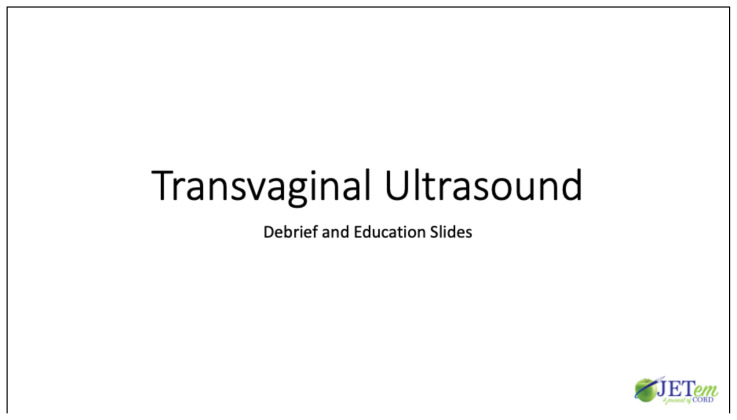
Please see associated PowerPoint file

**Table t21-9-3-s65:** Non-obstetrical causes of abdominal pain in pregnancy^15^,^15^

	Common	Uncommon
Upper abdomen	GERD (17–45%) Gallbladder disease (1%) Pneumonia (1 in 1000)	Acute hepatitis Pancreatic disease Rectus sheath hematoma Adrenal hemorrhage Hiatal hernia Venous thrombosis
Lower abdomen	Acute appendicitis (1 in 800) Anterior cutaneous nerve entrapment (2%) Round ligament pain (10–30%)	Nephrolithiasis (1 in 3300) IBD/colitis Diverticulitis Psoas abscess

**Table t22-9-3-s65:** 

**Critical Actions:** Obtain appropriate history of present illness and medical history from the patient Perform a focused physical exam Request the appropriate diagnostic tests on the patient, including labs, appropriate acquisition of fetal heart tones, and ultrasound imaging Correctly apply the endocavitary probe barrier and insert it with appropriate probe orientation. Demonstrate sweeping motion in transverse and sagittal planes Correctly interpret a TVUS IUP image and fetal heart tones using M-mode ultrasonography Recognize appendicitis as potential etiology to patient’s symptoms. Obtain correct diagnostic test for diagnosis of appendicitis in pregnancy Counsel patient on diagnosis and management of appendicitis in pregnancy Consult general surgery	0:00

**Table t23-9-3-s65:** 

	Milestone	Did not achieve level 1	Level 1	Level 2	Level 3
1	**Emergency Stabilization (PC1)**	□ Did not achieve Level 1	□ Recognizes abnormal vital signs	□ Recognizes an unstable patient, requiring intervention Performs primary assessment Discerns data to formulate a diagnostic impression/plan	□ Manages and prioritizes critical actions in a critically ill patient Reassesses after implementing a stabilizing intervention
2	**Performance of focused history and physical (PC2)**	□ Did not achieve Level 1	□ Performs a reliable, comprehensive history and physical exam	□ Performs and communicates a focused history and physical exam based on chief complaint and urgent issues	□ Prioritizes essential components of history and physical exam given dynamic circumstances
3	**Diagnostic studies (PC3)**	□ Did not achieve Level 1	□ Determines the necessity of diagnostic studies	□ Orders appropriate diagnostic studies. Performs appropriate bedside diagnostic studies/procedures	□ Prioritizes essential testing Interprets results of diagnostic studies Reviews risks, benefits, contraindications, and alternatives to a diagnostic study or procedure
4	**Diagnosis (PC4)**	□ Did not achieve Level 1	□ Considers a list of potential diagnoses	□ Considers an appropriate list of potential diagnosis May or may not make correct diagnosis	□ Makes the appropriate diagnosis Considers other potential diagnoses, avoiding premature closure
5	**Pharmacotherapy (PC5)**	□ Did not achieve Level 1	□ Asks patient for drug allergies	□ Selects an medication for therapeutic intervention, consider potential adverse effects	□ Selects the most appropriate medication and understands mechanism of action, effect, and potential side effects Considers and recognizes drug-drug interactions
6	**Observation and reassessment (PC6)**	□ Did not achieve Level 1	□ Reevaluates patient at least one time during case	□ Reevaluates patient after most therapeutic interventions	□ Consistently evaluates the effectiveness of therapies at appropriate intervals
7	**Disposition (PC7)**	□ Did not achieve Level 1	□ Appropriately selects whether to admit or discharge the patient	□ Appropriately selects whether to admit or discharge Involves the expertise of some of the appropriate specialists	□ Educates the patient appropriately about their disposition Assigns patient to an appropriate level of care (ICU/Tele/Floor) Involves expertise of all appropriate specialists
9	**General Approach to Procedures (PC9)**	□ Did not achieve Level 1	□ Identifies pertinent anatomy and physiology for a procedure Uses appropriate Universal Precautions	□ Obtains informed consent Knows indications, contraindications, anatomic landmarks, equipment, anesthetic and procedural technique, and potential complications for common ED procedures	□ Determines a back-up strategy if initial attempts are unsuccessful Correctly interprets results of diagnostic procedure
20	**Professional Values (PROF1)**	□ Did not achieve Level 1	□ Demonstrates caring, honest behavior	□ Exhibits compassion, respect, sensitivity and responsiveness	□ Develops alternative care plans when patients’ personal beliefs and decisions preclude standard care
22	**Patient centered communication (ICS1)**	□ Did not achieve level 1	□ Establishes rapport and demonstrates empathy to patient (and family) Listens effectively	□ Elicits patient’s reason for seeking health care	□ Manages patient expectations in a manner that minimizes potential for stress, conflict, and misunderstanding. Effectively communicates with vulnerable populations, (at risk patients and families)
23	**Team management (ICS2)**	□ Did not achieve level 1	□ Recognizes other members of the patient care team during case (nurse, techs)	□ Communicates pertinent information to other healthcare colleagues	□ Communicates a clear, succinct, and appropriate handoff with specialists and other colleagues Communicates effectively with ancillary staff

**Table t24-9-3-s65:** 

#1	Complete blood count (CBC)
#2	Comprehensive metabolic panel (CMP)
#3	ABO/Rh blood groups
#4	Beta human chorionic gonadotropin (B-hcg)
#5	Urinalysis (UA)
#6	Prothrombin time/international normalized ratio (PT/INR)
#7	Transabdominal ultrasound image revealing an empty uterus or pseudogestational sac
#8	Transvaginal ultrasound image revealing pelvic free fluid and no IUP

**Table t25-9-3-s65:** 

Critical Action	Met	Not Met	Comments
Obtain appropriate history of present illness and medical history from the patient.			
Perform focused physical exam on the patient **including pelvic exam.**			
Place patient on cardiopulmonary monitor and obtain IV access.			
Request appropriate diagnostic tests on the patient including serologic tests and ultrasound.			
Address patient’s symptoms with medication.			
Correctly apply endocavitary probe barrier and insert with appropriate probe orientation. Demonstrate sweeping motion in transverse and sagittal planes.			
Correctly interpret a TVUS with no identified IUP.			
Recognize hemorrhagic shock and initiate transfusion of blood products.			
Counsel patient on diagnosis and management of ruptured ectopic pregnancy.			
Verbalize need for transfer to facility with surgical capabilities.			
Consult OBGYN			

**Table t26-9-3-s65:** 

**Vitals:**	Heart Rate (HR) 111	Blood Pressure (BP) 102/62	Respiratory Rate (RR) 18
	Temperature (T) 37.0°C	Oxygen Saturation (O_2_Sat) 99% on room air	

**Table t27-9-3-s65:** 

*Complete blood count (CBC)*	
White blood count (WBC)	10.0 × 1000/mm^3^
Hemoglobin (Hgb)	7.8 g/dL
Hematocrit (HCT)	20.5%
Red blood cells (RBC)	5.5 × 1000/mm^3^
Mean Corpuscular Volume (MCV)	88 fL
Platelet (Plt)	250 × 1000/mm^3^
Neutrophils	3.5 × 1000/mm^3^
Lymphocytes	2.5 × 1000/mm^3^

**Table t28-9-3-s65:** 

*ABO/Rh*	O positive/Rh(−)
*B-hcg*	2,500 mlU/mL

**Table t29-9-3-s65:** 

*PT*	13 seconds
*INR*	1.0

**Table t30-9-3-s65:** 

*Comprehensive metabolic panel (CMP)*	
Sodium	135 mEq/L
Potassium	3.5 mEq/L
Blood Urea Nitrogen (BUN)	28 mg/dL
Creatinine (Cr)	0.87 mg/dL
Calcium	8.5 mg/dL
Chloride	100 mEq/L
Bicarbonate	24 mEq/L
Aspartate aminotransferase (AST)	35 U/L
Alanine aminotransferase (ALT)	45 U/L
Alkaline Phosphatase	55 U/L
Bilirubin	<1 mg/dL
Albumin	4.1 g/dL

**Table t31-9-3-s65:** 

*Urinalysis (UA)*	
Spec gravity:	1.002
Appearance	Cloudy
Color	Red
Glucose	Negative
Bilirubin	Negative
Ketones	Negative
Blood	Large
pH	7.0
Protein	Negative
Urobilinogen	Negative
Leukocyte esterase	Negative
Nitrites	Negative
Bacteria	Negative
White blood cells	0–5 WBCs/HPF
Red blood cells	50–100 RBCs/HPF
Squamous cells	15–20 cells/HPF

**Figure f11-9-3-s65:**
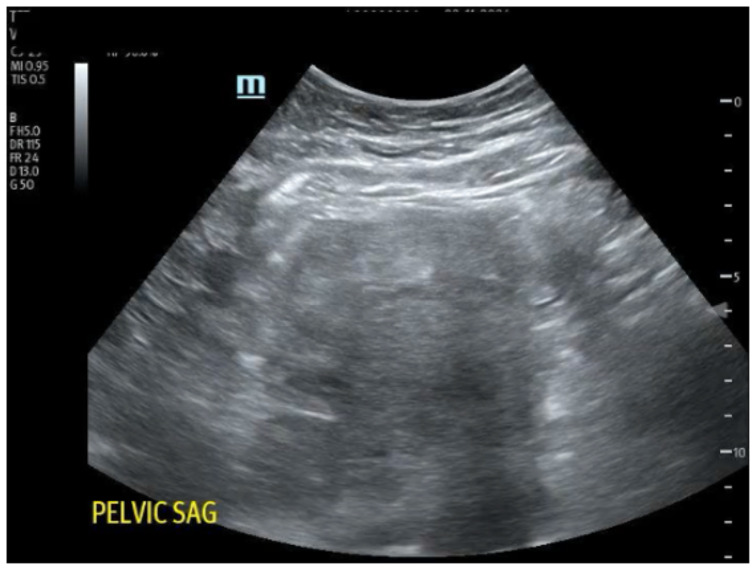
Stimulus 6

**Figure f12-9-3-s65:**
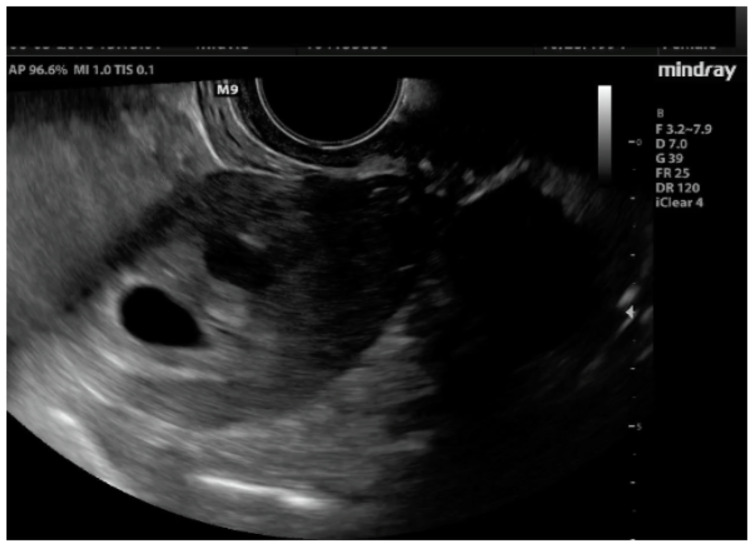
Stimulus 7

**Figure f13-9-3-s65:**
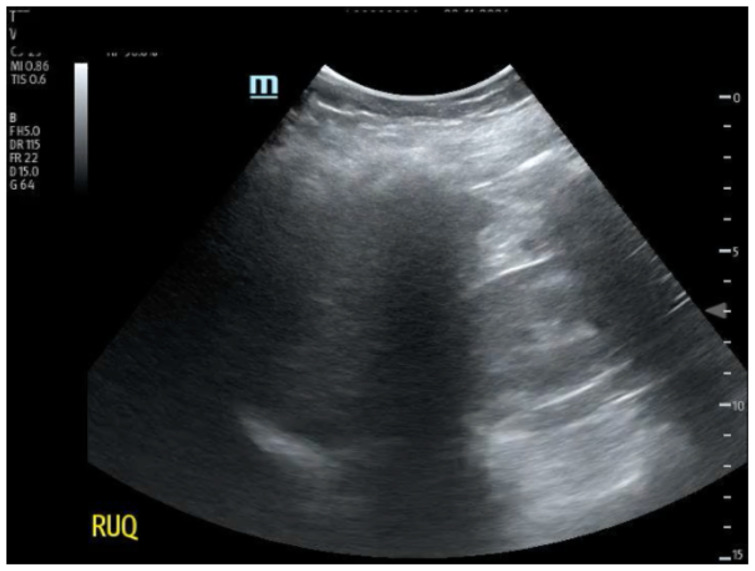
Stimulus 8

**Figure f14-9-3-s65:**
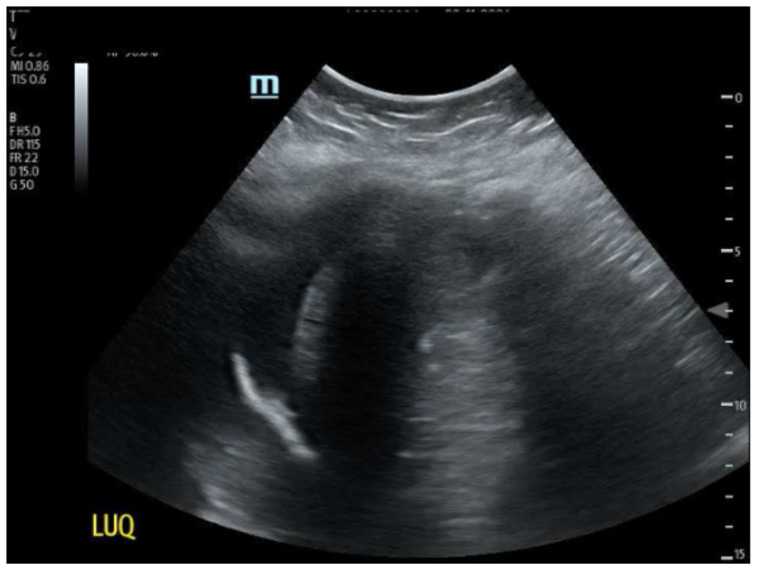
Stimulus 9

**Table t32-9-3-s65:** SIMULATION EVENTS TABLE Case 3 Vaginal Bleeding in Pregnancy:

Minute (State)	Participant Action/Trigger	Patient Status (Simulator Response) & Operator Prompts	Monitor Display (Vital Signs)
0:00 (Baseline)	The participant should place the patient on the monitor, obtain IV access, and obtain a history as well as conduct a physical exam. The participant may obtain a pelvic exam here.	Patient discloses history and is very uncomfortable during examination, which demonstrates significant pain in the lower abdomen.	T 37.4° C HR 111 BP 103/62 RR 18 O2 99%
2:00	Participant should request labs and provide pain medication to the patient. The participant may give the patient IVF bolus, but it will not change hemodynamic status. The labs may be displayed to the participant after exam and pain medication are administered. The participant should interpret that the patient has a positive pregnancy test and a low hemoglobin. If the participant has not yet done a pelvic exam, the participant should perform one as well as update the patient on labs results and next steps.	Patient will feel minimal relief with pain medication. The nurse will prompt the participant about pain medication if it is not given. The patient will have no improvement with IVF bolus. The pelvic examination should demonstrate heavy vaginal bleeding with moderate sized clots.	T 37.4° C HR 118 BP 103/62 RR 18 O2 99%
6:00	The participant may elect to obtain a FAST exam. The exam will be FAST positive. The participant may perform a transabdominal ultrasound. This will show a thickened endometrium and enlarged uterus but no IUP. The participant may consult OBGYN at this time for FAST+ and suspected ectopic. If so, move to time 12:00. Participant may perform a transvaginal ultrasound. They may go straight to the transvaginal ultrasound after performing the FAST exam. The participant should counsel the patient on the TVUS and obtain verbal consent.	Patient will demonstrate some relief if pain medications are re-dosed. If FAST is done, show STIMULUS 8+9. STIMULUS 6. If the participant does not opt to perform a transvaginal ultrasound, they may be prompted by the nurse that the probe is available. If the patient does not get information on the steps of a transvaginal ultrasound, the patient should ask what that is and what the steps are. STIMULUS 7	T 37.4° C HR 118 BP 103/62 RR 18 O2 99%
10:00	Participant performs a transvaginal ultrasound. The participant should interpret that there is no definitive IUP. If they elect to perform a FAST exam again, it will be FAST positive.	After the TVUS, the patient should report to the participant that they don’t feel very well and that they just felt a lot of liquid pool between their legs. STIMULUS 8 AND 9	T 37.4° C HR 120 BP 103/62 RR 18 O2 99%
12:00	Participant should re-examine the patient. They should note that there is heavier vaginal bleeding now with peritoneal abdominal pain and guarding. The participant should obtain a second IV access site if not done so already.	Patient becomes less responsive and more uncomfortable. She is still maintaining her airway but is more pale and tired appearing. If the participant does not obtain a second IV access site, the nurse should prompt them, asking if they would like another IV.	T 37.4° C HR 125 BP 76/43 RR 20 O2 99%
15:00	Participant should initiate blood products. Ideally, the participant will resuscitate with blood products 1:1:1 (RBC:FFP:platelets). The participant may also utilize TXA (1000mg IV). Participant should discuss additional resuscitative measures including MTP, pressors, and additional fluids.	Patient will have improved mentation and appearance after blood product transfusion. The patient will have continued vaginal bleeding and abdominal pain. The abdominal pain will lessen somewhat if pain medication is given. If the participant does not order blood products but instead orders infusion of IVF only, the BP will transiently improve but the patient’s appearance will worsen. BP will start to go down again unless blood products are given.	T 37.4° C HR 115 BP 99/68 RR 20 O2 99%
18:00 (Case Completion)	Participant should consult OBGYN for concern for a ruptured ectopic pregnancy, explaining the imaging results with positive pregnancy test, labs, and resuscitative efforts/interventions. The OBGYN will recommend having the patient transferred to the nearest hospital by fixed wing, which will take two hours. The participant should anticipate and verbalize what medications should be prepped and given while patient is en route.	Patient will continue to improve and be stable for transfer.	T 37.4° C HR 110 BP 105/74 RR 18 O2 99%

**Figure f15-9-3-s65:**
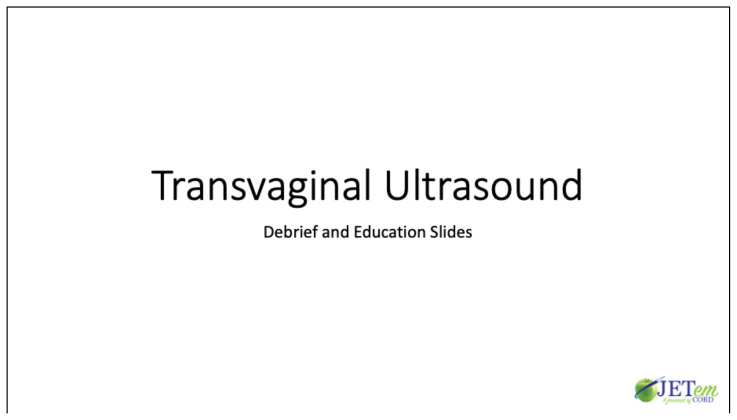
Please see associated PowerPoint file

**Figure 1 f16-9-3-s65:**
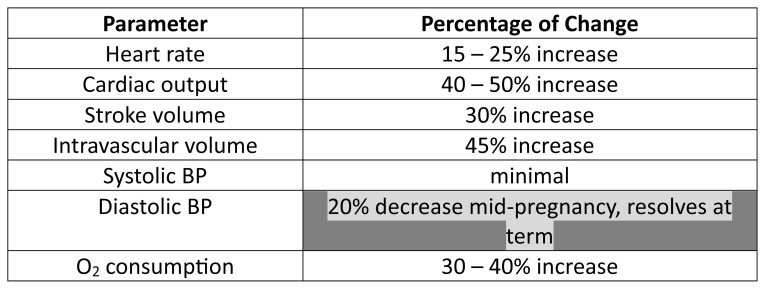
Physiologic changes in pregnancy (modified from Bhatia, et al.)^20^

**Table t33-9-3-s65:** 

**Critical Actions:** Request the appropriate diagnostic tests on the patient, including labs, and ultrasound imaging. Place patient on cardiopulmonary monitor and obtain IV access. Correctly apply endocavitary probe barrier and insert with appropriate probe orientation. Demonstrate sweeping motion in transverse and sagittal planes. Correctly interpret a TVUS with no identified IUP. Recognize hemorrhagic shock and initiate transfusion of blood products. Counsel patient on diagnosis and management of ruptured ectopic pregnancy. Verbalize need for transfer to facility with surgical capabilities. Consult OBGYN.	0:00

**Table t34-9-3-s65:** 

	Milestone	Did not achieve level 1	Level 1	Level 2	Level 3
1	**Emergency Stabilization (PC1)**	□ Did not achieve Level 1	□ Recognizes abnormal vital signs	□ Recognizes an unstable patient, requiring intervention Performs primary assessment Discerns data to formulate a diagnostic impression/plan	□ Manages and prioritizes critical actions in a critically ill patient Reassesses after implementing a stabilizing intervention
2	**Performance of focused history and physical (PC2)**	□ Did not achieve Level 1	□ Performs a reliable, comprehensive history and physical exam	□ Performs and communicates a focused history and physical exam based on chief complaint and urgent issues	□ Prioritizes essential components of history and physical exam given dynamic circumstances
3	**Diagnostic studies (PC3)**	□ Did not achieve Level 1	□ Determines the necessity of diagnostic studies	□ Orders appropriate diagnostic studies. Performs appropriate bedside diagnostic studies/procedures	□ Prioritizes essential testing Interprets results of diagnostic studies Reviews risks, benefits, contraindications, and alternatives to a diagnostic study or procedure
4	**Diagnosis (PC4)**	□ Did not achieve Level 1	□ Considers a list of potential diagnoses	□ Considers an appropriate list of potential diagnosis May or may not make correct diagnosis	□ Makes the appropriate diagnosis Considers other potential diagnoses, avoiding premature closure
5	**Pharmacotherapy (PC5)**	□ Did not achieve Level 1	□ Asks patient for drug allergies	□ Selects an medication for therapeutic intervention, consider potential adverse effects	□ Selects the most appropriate medication and understands mechanism of action, effect, and potential side effects Considers and recognizes drug-drug interactions
6	**Observation and reassessment (PC6)**	□ Did not achieve Level 1	□ Reevaluates patient at least one time during case	□ Reevaluates patient after most therapeutic interventions	□ Consistently evaluates the effectiveness of therapies at appropriate intervals
7	**Disposition (PC7)**	□ Did not achieve Level 1	□ Appropriately selects whether to admit or discharge the patient	□ Appropriately selects whether to admit or discharge Involves the expertise of some of the appropriate specialists	□ Educates the patient appropriately about their disposition Assigns patient to an appropriate level of care (ICU/Tele/Floor) Involves expertise of all appropriate specialists
9	**General Approach to Procedures (PC9)**	□ Did not achieve Level 1	Identifies pertinent anatomy and physiology for a procedure Uses appropriate Universal Precautions	□ Obtains informed consent Knows indications, contraindications, anatomic landmarks, equipment, anesthetic and procedural technique, and potential complications for common ED procedures	□ Determines a back-up strategy if initial attempts are unsuccessful Correctly interprets results of diagnostic procedure
20	**Professional Values (PROF1)**	□ Did not achieve Level 1	□ Demonstrates caring, honest behavior	□ Exhibits compassion, respect, sensitivity and responsiveness	□ Develops alternative care plans when patients’ personal beliefs and decisions preclude standard care
22	**Patient centered communication (ICS1)**	□ Did not achieve level 1	□ Establishes rapport and demonstrates empathy to patient (and family) Listens effectively	□ Elicits patient’s reason for seeking health care	□ Manages patient expectations in a manner that minimizes potential for stress, conflict, and misunderstanding. Effectively communicates with vulnerable populations, (at risk patients and families)
23	**Team management (ICS2)**	Did not achieve level 1	□ Recognizes other members of the patient care team during case (nurse, techs)	□ Communicates pertinent information to other healthcare colleagues	□ Communicates a clear, succinct, and appropriate handoff with specialists and other colleagues Communicates effectively with ancillary staff

**Figure 1:**
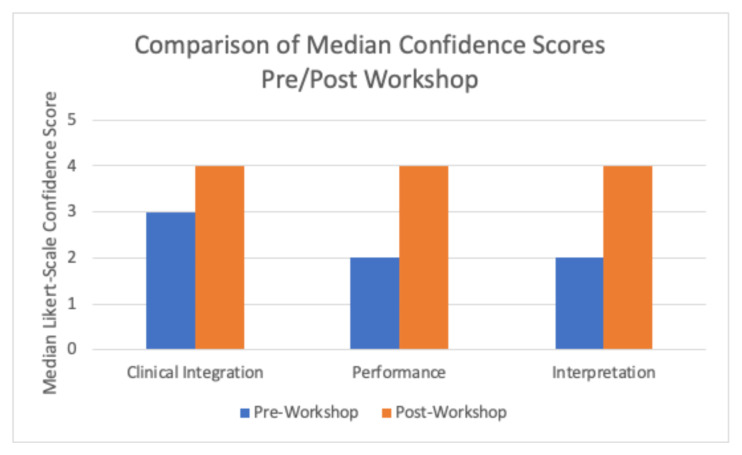
Comparison of median confidence scores pre and post-simulation

## INSTRUCTOR MATERIALS

**Case 1 Title:** Abdominal Pain in Pregnancy

**Case Description & Diagnosis (short synopsis):** This case involves a young female who presents with lower abdominal pain and is found to have a normal IUP on bedside transvaginal ultrasound, following an indeterminate transabdominal ultrasound (TAUS). This case allows the learner to recognize a normal appearing IUP and become familiar with the proper indication and technique surrounding transvaginal ultrasound.

**Equipment or Props Needed:**

- High or low fidelity simulation mannequin, female model with vaginal canal (for endocavitary probe insertion, does not need to be ultrasound compatible)
- Endocavitary probe
- Endocavitary probe covers, one per participant
- Monitors: blood pressure (BP) cuff, heart monitor leads, spO2 probe
- Mannequin moulage for heavy vaginal bleeding
- Peripheral IV materials
- Medication materials: empty or water filled containers and IV bags for intravenous fluids, blood products, tranexamic acid (TXA)
- TXA, analgesics, antibiotics
- Oxygen delivering system
- Lab data and imaging screen (or hard copy if no computer or screen available)

**Materials preferred but not required:**

- Ultrasound machine-compatible mannequin with endocavitary probe
- CAE Blue Phantom Combination IUP Ectopic Pregnancy Transvaginal Ultrasound Training Model
- Ultrasound gel

**Actors needed:**

One actor needed to play the role of a nurse. Another optional actor may play a spouse in a support role.

Stimulus Inventory:[Table t2-9-3-s65]

**Background and brief information:** 21-year-old female who is brought into a tertiary care, urban ED for a 3-day history of worsening lower abdominal pain.

**Initial presentation:** Patient presents for abdominal pain, described as a cramping sensation across the lower abdomen. It is constant and is relieved with ibuprofen. It feels like her typical menstrual pain, but she has not had her period yet this month. She has some associated nausea and one episode of vomiting this morning. She has had mild vaginal spotting, but no discharge or urinary symptoms.

**How the scene unfolds:** Upon initial presentation to the ED, learners will determine that the patient appears cooperative, comfortable, in no acute distress and able to provide an appropriate history. The patient provides a history and answers questions but appears nervous during the encounter. The patient is found to have lower abdominal tenderness on exam, although poorly localized. Learners should obtain labs, including a pregnancy test, which will be positive. If a pregnancy test is not ordered, the patient will prompt the learner by voicing concerns about a possible pregnancy. The patient will need to be informed of her test results and confirmatory imaging should be discussed among the participants. If a formal study is requested, learners will be informed that the sonographer is unavailable. A bedside TAUS should be performed, but an IUP will not be obvious. The patient will express confusion about the indeterminate results and the health of her baby. Learners should discuss confirmatory imaging with the patient and obtain verbal consent for a transvaginal ultrasound to be performed at the bedside. If this is not discussed, the nurse can prompt learners that a probe is available for use. The patient will ask the learners about the next steps if she does not receive proper information about the plan. Learners should obtain verbal consent, utilize a sterile probe cover, correctly insert and orient the endocavitary probe, and accurately interpret displayed images. The patient will remain hemodynamically stable during this entire encounter. Once an IUP is established, the patient should be updated regarding the results. Finally, a disposition plan should be discussed, including follow up with Obstetrics and Gynecology (OBGYN) as an outpatient with appropriate return precautions.

**Critical Actions Case 1:** Abdominal Pain in Pregnancy[Table t3-9-3-s65]

**Case 1 Title:** Abdominal Pain in Pregnancy

**Chief Complaint:** abdominal pain[Table t4-9-3-s65]

**General Appearance:** Resting comfortably in an upright position and in no acute distress. Appears visibly nervous and slightly withdrawn.

**Primary Survey:**

- **Airway:** speaking in full sentences
- **Breathing:** no distress, clear breath sounds bilaterally
- **Circulation:** normal pulses and heart sounds, not diaphoretic or clammy

**History:**

- **History of present illness:** Patient is a 21-year-old female who presents to the ED with abdominal pain. The pain is characterized as a constant, cramping sensation across the lower abdomen. It is somewhat alleviated with ibuprofen. It feels like her typical menstrual pain, although she missed her menses last month. She has associated nausea and one episode of vomiting this morning. She reports mild vaginal spotting, but no discharge or urinary symptoms.
- **Additional history (must ask):**
  ○ **Past medical history:** iron deficiency anemia
  ○ **Past surgical history:** cholecystectomy three years ago
  ○ **Medications:** iron supplements
  ○ **Allergies:** None
  ○ **Social history:** no drug, alcohol, or smoking history. Her last menstrual period (LMP) was six weeks ago. She has sex with one partner. She has never been pregnant before.
  ○ **Family history:** non-contributory

**Secondary Survey/Physical Examination:**

- **General appearance:** well appearing, no acute distress
- **HEENT:** normal
- **Neck:** normal
- **Heart:** normal
- **Lungs:** normal
- **Abdominal/GI:** vague lower abdominal tenderness. No rebound, guarding or rigidity. Active bowel sounds. Abdomen is not gravid.
- **Genitourinary:** if a pelvic exam is performed, there is a closed cervical os without bleeding or discharge, normal external genitalia.
- **Rectal:** normal
- **Extremities:** normal
- **Back:** normal
- **Neuro:** normal
- **Skin:** normal
- **Lymph:** normal
- **Psych:** normal

**Stimulus Case 1 Abdominal Pain in Pregnancy:** [Table t5-9-3-s65] [Table t6-9-3-s65] [Table t7-9-3-s65] [Table t8-9-3-s65]

*Transabdominal Ultrasound*

Author’s own image

See associated PowerPoint with videos.[Fig f3-9-3-s65]

*Transvaginal Ultrasound*

Author’s own images

See associated PowerPoint with videos.[Fig f4-9-3-s65]

*Transvaginal Ultrasound*

Author’s own images

See associated PowerPoint with videos.[Fig f5-9-3-s65]

## OPERATOR MATERIALS

[Table t9-9-3-s65]

**Diagnosis:**

IUP

**Disposition:**

Discharge with OBGYN referral for close follow-up

## DEBRIEFING AND EVALUATION PEARLS

### Case 1: Abdominal Pain in Pregnancy

[Fig f6-9-3-s65]

**Pearls:**

- Transvaginal ultrasound (TVUS) is the test of choice for diagnosis of IUP and ectopic pregnancy.^9^,^14^
- Transvaginal ultrasound has a sensitivity of 95% and specificity of 99% for IUP identification.^9^,^14^
- When a TVUS was performed by an ED physician after an indeterminant TAUS, they identified 48% more IUPs.^12^
- The goal of ED TVUS is not to identify ectopic pregnancies or other pathology; it is to identify an IUP only.
- All POCUS studies are reviewed by ultrasound ED faculty for quality assurance.
- If any study is unclear, indeterminant, or concerning, a formal ultrasound study should be obtained.

**Other debriefing points:**

Patients who present to the ED and are identified as having a first trimester pregnancy, but with indeterminate TAUS imaging, would benefit from a bedside transvaginal point-of-care ultrasound (TVPOCUS). TVPOCUS has been shown to identify 48% more IUPs, shave 35–75 minutes off ED length of stay, and save $181–$2,826 per patient.^11^–^13^ ED-performed TVUS is also comparable to radiology-performed TVUS (94% agreement) with a sensitivity of 90 – 94% and a specificity 98%.^14^ When surveyed, patients were comfortable with EMP-performed TVUS and were mostly concerned about the status of their pregnancy.^10^ Additionally, 96% of women reported that they were willing to have another TVUS study following a bedside TVPOCUS if necessary.^10^

**Wrap up:**

There should be a discussion at debrief regarding how to incorporate TVPOCUS into clinical care during a shift. This should include the proper steps and technique for performing TVUS, with particular focus on basic ultrasound functions, probe indicator and orientation, image landmarks and anatomy, proper barrier use, and institution-specific storage and sterilization protocols.

Steps when performing a TVPOCUS:

1. Verbal consent
2. Chaperone that is same gender as patient
3. Always use sterile gel packet and TVUS probe cover
4. Insertion, orientation, and sweeping in transverse and sagittal planes
5. Proper disinfection and storage protocols (institution specific)
6. Documentation of images obtained, findings, and interpretation (institution specific)

## SIMULATION ASSESSMENT

### Case 1: Abdominal Pain in Pregnancy

Learner: _________________________________________

***Assessment Timeline***

This timeline is to help observers assess their learners. It allows observer to make notes on when learners performed various tasks, which can help guide debriefing discussion.[Table t10-9-3-s65]

**Critical Actions:**

□ Obtain an appropriate HPI and medical history from the patient.
□ Perform a focused physical exam.
□ Request the appropriate diagnostic tests on the patient, including a pregnancy test and bedside ultrasound imaging.
□ Correctly apply the endocavitary probe barrier and insert it with appropriate probe orientation. Demonstrate sweeping motion in transverse and sagittal planes.
□ Correctly interpret a TVUS IUP image.
□ Educate the patient on diagnosis and disposition.

**Summative and formative comments:**

**Milestones assessment:** [Table t11-9-3-s65]

## INSTRUCTOR MATERIALS

**Case 2 Title:** Right Lower Quadrant Abdominal Pain in Pregnancy

**Case Description & Diagnosis (short synopsis):** This case involves a 31-year-old female who presents with a one-day history of worsening right lower abdominal pain. She is 10 weeks pregnant and reports having appropriate prenatal care and OBGYN follow up. Her examination is notable for right-sided lower abdominal tenderness without peritoneal signs and a gravid abdomen. Pelvic examination is unremarkable. A bedside TAUS should be performed without demonstrable fetal heart tones. A TVPOCUS should follow, revealing a proper fetal heart rate and trace pelvic free fluid. At this point, the learner should recognize free fluid and the possibility of an alternative diagnosis, including a high suspicion for appendicitis. A confirmatory imaging modality such as formal abdominal ultrasound, computed tomograpby (CT), or magnetic resonance imaging (MRI) must be obtained. After making a diagnosis of acute appendicitis in pregnancy, the appropriate subspecialties need to be consulted.

**Equipment or Props Needed:**

- High or low fidelity simulation mannequin, female model with vaginal canal (for endocavitary probe insertion, does not need to be ultrasound compatible)
- Endocavitary probe
- Endocavitary probe covers, one per participant
- Monitors: BP cuff, heart monitor leads, spO2 probe
- Mannequin moulage for heavy vaginal bleeding
- Peripheral IV materials
- Medication materials: empty or water-filled containers and IV bags for intravenous fluids, analgesics, antibiotics
- Oxygen delivering system
- Lab data and imaging screen (or hard copy if no computer or screen available)

**Materials preferred but not required:**

- Ultrasound machine-compatible mannequin with endocavitary probe
- CAE Blue Phantom Combination IUP Ectopic Pregnancy Transvaginal Ultrasound Training Model
- Ultrasound gel

**Actors needed:**

One actor needed to play the role of a nurse. Another optional actor may play a spouse in a support role.

**Stimulus Inventory:** [Table t12-9-3-s65]

**Background and brief information:** 31-year-old female who is brought into a tertiary care, urban ED for a one-day history of worsening lower abdominal pain.

**Initial presentation:** Thirty-one-year-old woman presenting with one day of worsening abdominal pain. The pain is described as vague with a cramping sensation that is worse on the right side. It has gradually gotten worse despite taking acetaminophen for pain. It does not worsen with movement. She is 10 weeks pregnant and has had appropriate prenatal care and regular OBGYN follow up. She denies diarrhea, fevers, or vomiting, but has nausea and a decreased appetite. She has no vaginal bleeding or urinary symptoms.

**How the scene unfolds:** Upon initial presentation to the ED, learners will determine that the patient appears uncomfortable, lying supine and speaking in few word sentences due to pain. The patient provides a history and answers questions appropriately but is uncomfortable during the exam. The patient is found to have lower abdominal tenderness on exam, localized to the right lower quadrant. A pelvic examination should be performed and will be unremarkable. She should receive analgesics which will result in significant relief. Learners should obtain labs and a bedside TAUS to assess for IUP and fetal status. When performed, images will be positive for an IUP, but with identifiable fetal heart tones. Given difficulty assessing for heart tones on transabdominal imaging, a TVPOCUS should be performed after proper patient counseling and consent. TVPOCUS should demonstrate appropriate fetal heart tones and free fluid in the pelvis. This should be a stopping point for the participants and a discussion should be held regarding alternative diagnoses in the setting of pregnancy with right lower quadrant abdominal pain and free fluid in the pelvis on imaging. Appendicitis should be a top consideration. With this, learners should discuss the different confirmatory imaging modalities, including formal abdominal ultrasound, CT, and MRI. Shared decision-making with the patient should be conducted, followed by confirmatory imaging. Once appendicitis is confirmed, the patient should be updated regarding results. She should be given IV antibiotics and placed on NPO status. General surgery and OBGYN services should be consulted. The patient has a final diagnosis of acute appendicitis in first trimester pregnancy and will go to the operating room (OR) for definitive management.

**Critical Actions Case 2:** Right Lower Quadrant Abdominal Pain in Pregnancy[Table t13-9-3-s65]

**Case 2 Title:** Right Lower Quadrant Abdominal Pain in Pregnancy

**Chief Complaint:** right lower quadrant abdominal pain[Table t14-9-3-s65]

**General Appearance:** Lying supine and uncomfortable appearing on exam, holding her right lower abdomen

**Primary Survey:**

- **Airway:** speaking in full sentences
- **Breathing:** no distress, clear breath sounds bilaterally
- **Circulation:** tachycardic with normal distal pulses and heart sounds

**History:**

- **History of present illness:** 31-year-old woman who presents with one day of worsening abdominal pain. She describes it as a vague and cramping sensation that is worse on the right side. It has gradually worsened throughout the day despite taking acetaminophen for pain. It does not worsen with movement. She is 10 weeks pregnant and has had regular OBGYN follow up. She denies diarrhea, fevers, or vomiting, but has had a decreased appetite with associated nausea. She denies vaginal bleeding or urinary symptoms.
- **Additional history (must ask)**:
  ○ **Past medical history:** none
  ○ **Past surgical history:** none
  ○ **Medications:** none
  ○ **Allergies:** none
  ○ **Social history:** no drug, alcohol, or smoking history
  ○ **Family history:** non-contributory

**Secondary Survey/Physical Examination:**

- **General appearance:** Appears uncomfortable, but otherwise in no acute distress.
- **HEENT:** normal
- **Neck:** normal
- **Heart:** tachycardic but regular rhythm.
- **Lungs:** normal
- **Abdominal/GI:** right-sided lower abdominal tenderness without guarding or rebound. Normoactive bowel sounds. Gravid uterus consistent with reported dates. No costovertebral angle (CVA) tenderness.
- **Genitourinary:** Closed os, no blood, discharge, or cervical motion tenderness.
- **Rectal:** normal
- **Extremities:** normal
- **Back:** normal
- **Neuro:** normal
- **Skin:** normal
- **Lymph:** normal
- **Psych:** normal

**Stimulus Case 2 Right Lower Quadrant Abdominal Pain in Pregnancy:** [Table t15-9-3-s65] [Table t16-9-3-s65] [Table t17-9-3-s65] [Table t18-9-3-s65] [Table t19-9-3-s65]

*Transabdominal Ultrasound: No fetal heart tones (FHT)*

Author’s own image

See associated PowerPoint with videos.[Fig f7-9-3-s65]

*Transvaginal Ultrasound: FHT 127*

Author’s own images

See associated PowerPoint with videos.[Fig f8-9-3-s65]

*Transvaginal Ultrasound: M-mode Image*

Author’s own images

See associated PowerPoint with videos.[Fig f9-9-3-s65]

*Magnetic Resonance Imaging*

Impression: +IUP. Acute uncomplicated appendicitis

## OPERATOR MATERIALS

[Table t20-9-3-s65]

**Diagnosis:**

Acute appendicitis

**Disposition:**

To the OR with surgery and OBGYN

## DEBRIEFING AND EVALUATION PEARLS

### Case 2: Right Lower Quadrant Abdominal Pain in Pregnancy

[Fig f10-9-3-s65]

**Pearls:**

- Acute appendicitis in pregnancy typically occurs in the second and third trimester.^15^,^16^
- Risk of fetal loss is to 6% in cases of complicated appendicitis.^15^,^16^
- Gravid women may have atypical pain presentations, including pain to the right flank, right upper quadrant, or no pain at all.^15^,^16^
- Laboratory studies can be unreliable. Leukocytosis can be a normal finding in pregnancy. C-Reactive Protein (CRP) can be elevated in pregnancy as well.^15^,^16^
- Microscopic hematuria and sterile pyuria can occur in acute appendicitis (20%).^15^,^16^

**Other debriefing points:**

Debriefing should include a discussion on non-obstetrical causes of abdominal pain in pregnancy and “red herrings.” A discussion about risks to the fetus is also important for anticipatory guidance. Additionally, certain lab abnormalities such as sterile pyuria and leukocytosis can be normal in pregnancy. Furthermore, given the need for definitive imaging, learners should be debriefed on proper risk/benefit discussions when obtaining imaging studies such as abdominal ultrasound, MRI, and CT in pregnancy. This should include a review of the evidence regarding radiation exposure to the growing fetus.[Table t21-9-3-s65]

**Wrap up:**

There should be a discussion at debrief regarding how to incorporate TVPOCUS into clinical care during a shift. This should include the proper steps and technique for performing TVUS with particular focus on basic ultrasound functions, probe indicator and orientation, and image landmarks and anatomy. In this specific case, measuring fetal heart tones should be reviewed by using M-mode ultrasonography and utilization of the appropriate beat-to-beat variability machine measurement tools. Additionally, a discussion surrounding the concept of as low as reasonably acceptable (ALARA) to include mechanical and thermal indices of sonographic imaging would be beneficial because using spectral doppler to assess fetal heart tones is not recommended. Finally, proper barrier use and institution-specific storage and sterilization protocols should be reviewed.

Steps when performing a TVPOCUS:

1. Verbal consent
2. Chaperone that is same gender as patient
3. Always use sterile gel packet and TVUS probe cover
4. Insertion, orientation, and sweeping in transverse and sagittal planes
5. Proper disinfection and storage protocols (institution specific)
6. Documentation of images obtained, findings, and interpretation (institution specific)

## SIMULATION ASSESSMENT

### Case 2: Right Lower Quadrant Abdominal Pain in Pregnancy

Learner: _________________________________________

***Assessment Timeline***

This timeline is to help observers assess their learners. It allows observer to make notes on when learners performed various tasks, which can help guide debriefing discussion.[Table t22-9-3-s65]

**Critical Actions:**

□ Obtain appropriate history of present illness and medical history from the patient
□ Perform a focused physical exam
□ Request the appropriate diagnostic tests on the patient, including labs, appropriate acquisition of fetal heart tones, and ultrasound imaging
□ Correctly apply the endocavitary probe barrier and insert it with appropriate probe orientation. Demonstrate sweeping motion in transverse and sagittal planes
□ Correctly interpret a TVUS IUP image and fetal heart tones using M-mode ultrasonography
□ Recognize appendicitis as potential etiology to patient’s symptoms.
□ Obtain correct diagnostic test for diagnosis of appendicitis in pregnancy
□ Counsel patient on diagnosis and management of appendicitis in pregnancy
□ Consult general surgery

**Summative and formative comments:**

**Milestones assessment:** [Table t23-9-3-s65]

## INSTRUCTOR MATERIALS

**Case 3 Title:** Vaginal Bleeding in Pregnancy

**Case Description & Diagnosis (short synopsis):** This case involves a 40-year-old female who presents with a one-day history of worsening abdominal pain and vaginal bleeding. The participant works at a critical access hospital where an ultrasound tech is on call but needs one to two hours to drive to the hospital. A family medicine physician can admit patients, but there are no OBGYN specialty services available. Transfer out to a higher level of care is typically by air if weather permits. CT imaging, a bedside ultrasound machine, and most medications are available. The patient presents with diffuse lower abdominal pain, mostly to the right side with radiation to her back. She describes heavy vaginal bleeding since pain onset. The patient’s vitals are significant for tachycardia, and a “soft” BP. On arrival, she appears uncomfortable, with pale, clammy skin. The pelvic exam demonstrates heavy bleeding with large clots. Labs show a positive pregnancy test and anemia. A focused assessment with sonography for trauma (FAST) is negative and a follow up TVPOCUS reveals an empty uterus and free fluid in the pelvis. The patient decompensates, prompting participants to expeditiously resuscitate the patient with blood products and other appropriate critical interventions. The patient is diagnosed with a ruptured ectopic pregnancy and after proper stabilization is transferred by air to a higher level of care.

**Equipment or Props Needed:**

- High or low fidelity simulation mannequin, female model with vaginal canal (for endocavitary probe insertion, does not need to be ultrasound compatible)
- Endocavitary probe
- Endocavitary probe covers, one per participant
- Monitors: BP cuff, heart monitor leads, spO2 probe
- Mannequin moulage for heavy vaginal bleeding
- Peripheral IV materials
- Medication materials: empty or water filled containers and IV bags for intravenous fluids, TXA, pressors, analgesics, antibiotics
- Oxygen delivering system
- Lab data and imaging screen (or hard copy if no computer or screen available)

**Materials preferred but not required:**

- Ultrasound machine-compatible mannequin with endocavitary probe
- CAE Blue Phantom Combination IUP Ectopic Pregnancy Transvaginal Ultrasound Training Model
- Ultrasound gel

**Actors needed:**

One actor needed to play the role of a nurse. Another optional actor may play a spouse in a support role.

**Stimulus Inventory:** [Table t24-9-3-s65]

**Background and brief information:** 40-year-old female who is brought into a critical access ED with limited subspecialty services for a one-day history of worsening lower abdominal pain and heavy vaginal bleeding.

**Initial presentation:** 40-year-old woman presenting with one day of worsening abdominal pain and vaginal bleeding. The pain is described as cramping and sharp, mostly localized to the right lower abdomen with involvement of her right low back. Her bleeding is heavy. She is soaking one pad every two hours with passage of large clots. She feels lightheaded but denies syncope. She denies fever, chills, vomiting or diarrhea. She notes nausea and generalized fatigue since her symptoms started.

**How the scene unfolds:** Upon initial presentation to the ED, learners will determine that the patient appears uncomfortable, lying supine, and speaking in few-word sentences due to pain. She appears somewhat sleepy as well. The patient provides a history and answers questions appropriately but is uncomfortable during the exam. The patient is found to have lower abdominal tenderness on exam, localized to the right lower quadrant and involving her right lower back. She receives minimal relief with analgesics. Her presenting vital signs reveal tachycardia and a “soft” BP. Given her history and exam, learners should order large bore IV access and start with crystalloids for initial resuscitation. Additionally, the use of simulated moulage is important during the exam prep because this will be hidden under the blanket. Once a proper exam is performed, learners will reveal the moulage on the gurney. This should prompt a sensation of urgency. A pelvic exam should be performed along with sonographic imaging. The pelvic examination should demonstrate heavy bleeding with moderately sized blood clot passage. Ultrasound imaging should demonstrate free pelvic fluid and lack of an IUP. Labs should be sent and will return with a positive pregnancy test and evidence of anemia. A FAST examination is an important step during this process and should be performed. The patient will have an acute change in her clinical and hemodynamic status, revealing progressive hemodynamic instability. Blood pressure should drop with a rising heart rate in the setting of worsening vaginal bleeding. Learners should recognize these changes and act immediately. Discussion should be facilitated on resuscitation options, including IV fluids, TXA, blood products, fresh frozen plasma (FFP), platelets, massive transfusion protocols (MTP), and pressors. Ultimately, the patient will improve if appropriate measures are taken but remain in critical condition. Learners should then recognize the importance of coordinating immediate air transport (with appropriate on-board equipment and personnel) to a hospital with OBGYN services for higher level of care. The final diagnosis of this case is ruptured ectopic pregnancy.

**Critical Actions Case 3:** Vaginal Bleeding in Pregnancy[Table t25-9-3-s65]

**Case 3 Title:** Vaginal Bleeding in Pregnancy

**Chief Complaint:** Right-sided abdominal pain, back pain, and vaginal bleeding[Table t26-9-3-s65]

**General Appearance:** Lying supine and uncomfortable appearing on exam, holding her right lower abdomen. Skin is damp and clammy. She seems slightly lethargic.

**Primary Survey:**

- **Airway:** speaking in full sentences
- **Breathing:** no distress, clear breath sounds bilaterally
- **Circulation:** tachycardic with normal distal pulses and heart sounds. Skin cool and clammy.

**History:**

- **History of present illness:** The patient has had three days of worsening abdominal pain and vaginal bleeding that started today. The pain is a cramping and sharp sensation that is mostly in the right lower quadrant and radiates around to her right lower back. The vaginal bleeding is moderately heavy. She is soaking one pad every two hours and has passed multiple large clots. She feels lightheaded, but denies syncope, chest pain, shortness of breath, or urinary symptoms.
- **Additional history (must ask):**
  ○ **Past medical history:** polycystic ovarian disease, hypertension
  ○ **Past surgical history:** cholecystectomy
  ○ **Social history:** Has multiple sexual partners but has never been pregnant. (Last menstrual period) LMP was eight weeks ago, but her periods are described as irregular. Denies drugs or alcohol use, but smokes tobacco, ½ pack-per-day
  ○ **Medications:** oral-contraceptive pills
  ○ **Allergies:** penicillin (rash)
  ○ **Family history:** non-contributory

**Secondary Survey/Physical Examination:**

- **General appearance:** Lying supine and uncomfortable appearing on exam, holding her right lower abdomen. Skin is damp and clammy. She seems slightly lethargic and intermittently moans in pain.
- **HEENT:** normal
- **Neck:** normal
- **Heart:** tachycardic but regular rhythm
- **Lungs:** normal
- **Abdominal/GI:** soft, no rigidity. Right lower quadrant tenderness to palpation with involuntary guarding
- **Genitourinary:** Blood in the vaginal vault with presence of clots. Open os with active bleeding and pooling in the vault. No identifiable tissue.
- **Rectal:** normal
- **Extremities:** normal
- **Back:** normal
- **Neuro:** normal
- **Skin:** cool, damp
- **Lymph:** normal
- **Psych:** normal

**Stimulus Case 3 Vaginal Bleeding in Pregnancy:** [Table t27-9-3-s65] [Table t28-9-3-s65] [Table t29-9-3-s65] [Table t30-9-3-s65] [Table t31-9-3-s65]

*Transabdominal Ultrasound*

Author’s own image

See associated PowerPoint with videos.[Fig f11-9-3-s65]

*Transvaginal Ultrasound*

Author’s own images

See associated PowerPoint with videos.[Fig f12-9-3-s65]

*FAST Exam*

Author’s own images

See associated PowerPoint with videos.[Fig f13-9-3-s65][Fig f14-9-3-s65]

## OPERATOR MATERIALS

[Table t32-9-3-s65]

**Diagnosis:**

Ruptured ectopic pregnancy

**Disposition:**

Fixed wing transfer to hospital with OBGYN services

## DEBRIEFING AND EVALUATION PEARLS

### Case 3: Vaginal Bleeding in Pregnancy

[Fig f15-9-3-s65]

**Pearls:**

- 25% of pregnant women experience bleeding in the first trimester.^17^,^19^
- Discriminatory zones should be considered, but the exact numbers are somewhat controversial. Generally, B-hcg levels above 3,000 mIU/mL should allow for visualization of an IUP on TVUS.^17^,^19^
- Ectopic pregnancy has a 1–2% incidence in the United States and accounts for 6% of maternal deaths.^17^,^19^
- Ectopic pregnancies can have a slower rise in B-hcg levels.
- Most common site for ectopic pregnancy is the ampulla of the fallopian tube.
- An extrauterine gestation is not seen 15–30% of the time.^17^,^19^
- TVUS increases the sensitivity and specificity of the diagnosis of ectopic pregnancy.^17^,^19^
- Free pelvic fluid and a positive B-hcg are 70% specific and 63% sensitive for ectopic pregnancy.^17^,^19^

**Causes of vaginal bleeding in the first trimester of pregnancy include:**

- Miscarriage
- Subchorionic hemorrhage
- Ectopic pregnancy
- Gestational trophoblastic disease
- Demise of a twin
- Implantation bleeding

**Other debriefing points:**

Debriefing should include a discussion surrounding the differential diagnosis of abdominal pain and vaginal bleeding in a female. Learners should recognize important physiologic changes in the mother during pregnancy.

Furthermore, the progressively worsening vital sign abnormalities during the simulation should prompt the learners to recognize that the patient is experiencing a ruptured ectopic pregnancy with severe blood loss. Learners should then understand the importance of recognizing features of hemorrhagic shock^18^ and act expeditiously. The utility of FAST examination in this setting should also be evaluated because assessing for RUQ free fluid reduces time to diagnosis and is highly suggestive that operative management will be necessary. The debrief should address various treatment options, such as blood products, TXA, MTP, crystalloids and vasopressors. Discussion on the proper ratio of transfusion products during MTP and the logistics of initiating the process should be addressed. Learners should consider key differences in resuscitating hemodynamically unstable patients at critical access hospitals and the logistics of air transport to a higher level of care.[Fig f16-9-3-s65]

**Wrap up:**

There should be a discussion at debrief regarding how to incorporate TVPOCUS into clinical care during a shift. This should include the proper steps and technique for performing TVUS, with particular focus on basic ultrasound functions, probe indicator and orientation, and image landmarks and anatomy. Measuring fetal heart tones should be reviewed by using M-mode ultrasonography and utilization of the appropriate beat-to-beat variability machine measurement tools. Additionally, a discussion surrounding the utility of the FAST examination as well as sonographic features of a ruptured ectopic pregnancy should be had. Finally, proper barrier use and institution-specific storage and sterilization protocols should be reviewed.

Steps when performing a TVPOCUS:

1. Verbal consent
2. Chaperone that is same gender as patient
3. Always use sterile gel packet and TVUS probe cover
4. Insertion, orientation, and sweeping in transverse and sagittal planes
5. Proper disinfection and storage protocols (institution specific)
6. Documentation of images obtained, findings, and interpretation (institution specific)

## SIMULATION ASSESSMENT

### Case 3: Vaginal Bleeding in Pregnancy

Learner: _________________________________________

***Assessment Timeline***

This timeline is to help observers assess their learners. It allows observer to make notes on when learners performed various tasks, which can help guide debriefing discussion.[Table t33-9-3-s65]

**Critical Actions:**

□ Request the appropriate diagnostic tests on the patient, including labs, and ultrasound imaging.
□ Place patient on cardiopulmonary monitor and obtain IV access.
□ Correctly apply endocavitary probe barrier and insert with appropriate probe orientation.
□ Demonstrate sweeping motion in transverse and sagittal planes.
□ Correctly interpret a TVUS with no identified IUP.
□ Recognize hemorrhagic shock and initiate transfusion of blood products.
□ Counsel patient on diagnosis and management of ruptured ectopic pregnancy.
□ Verbalize need for transfer to facility with surgical capabilities.
□ Consult OBGYN.

**Summative and formative comments:**

**Milestones assessment:** [Table t34-9-3-s65]
