# Supplementary material for: High-Fidelity Simulation with Transvaginal Ultrasound in the Emergency Department
Source: J Educ Teach Emerg Med. 2024 Jul 31;9(3):S65–S135. doi: 10.21980/J8606Q (PMC11312878; doi:10.21980/J8606Q)
Supplement: Supplementary file 1 [file 9-3-S65-Supp1.pptx]

## Slide 1
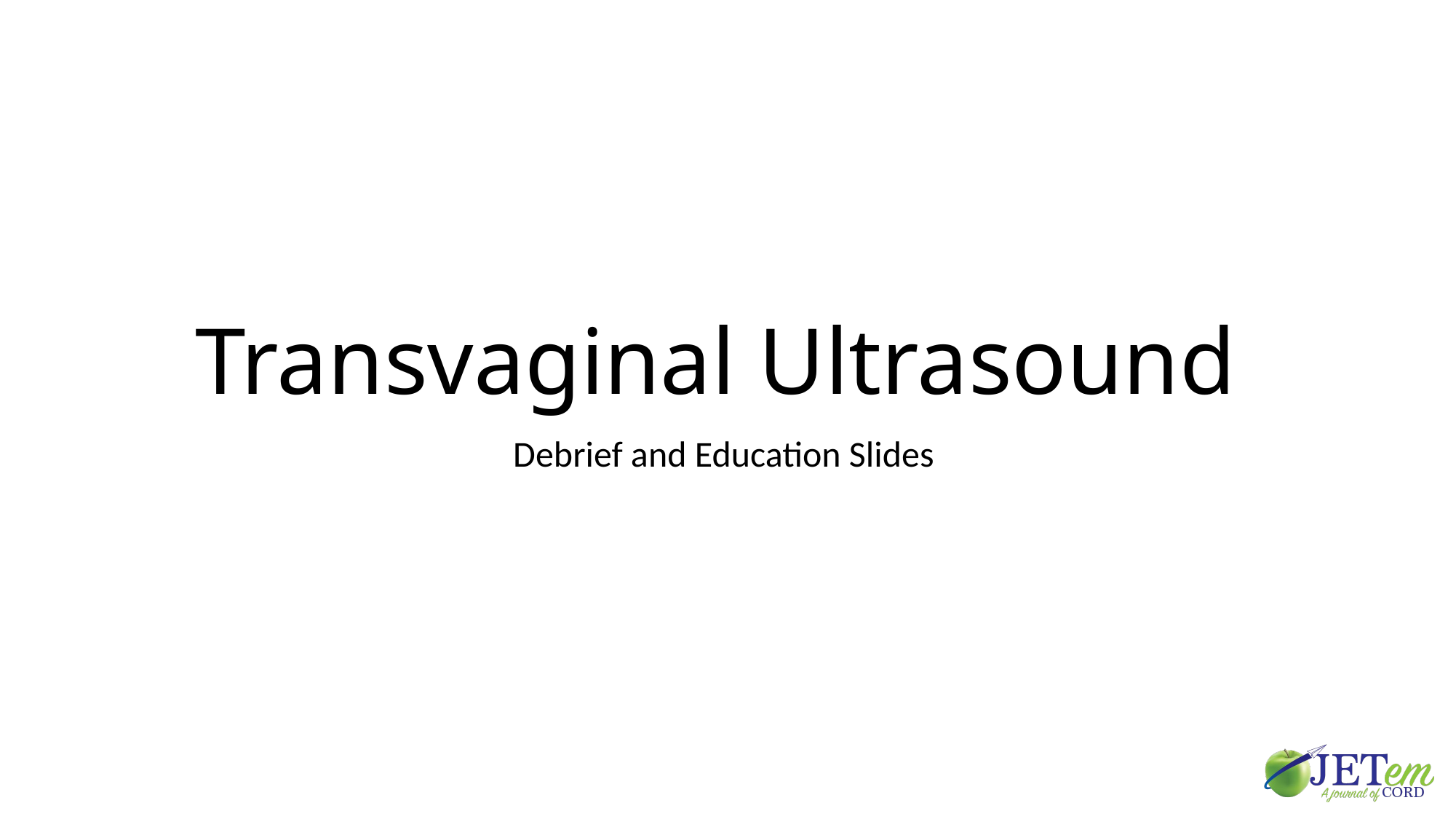

# Transvaginal Ultrasound
Debrief and Education Slides

## Slide 2
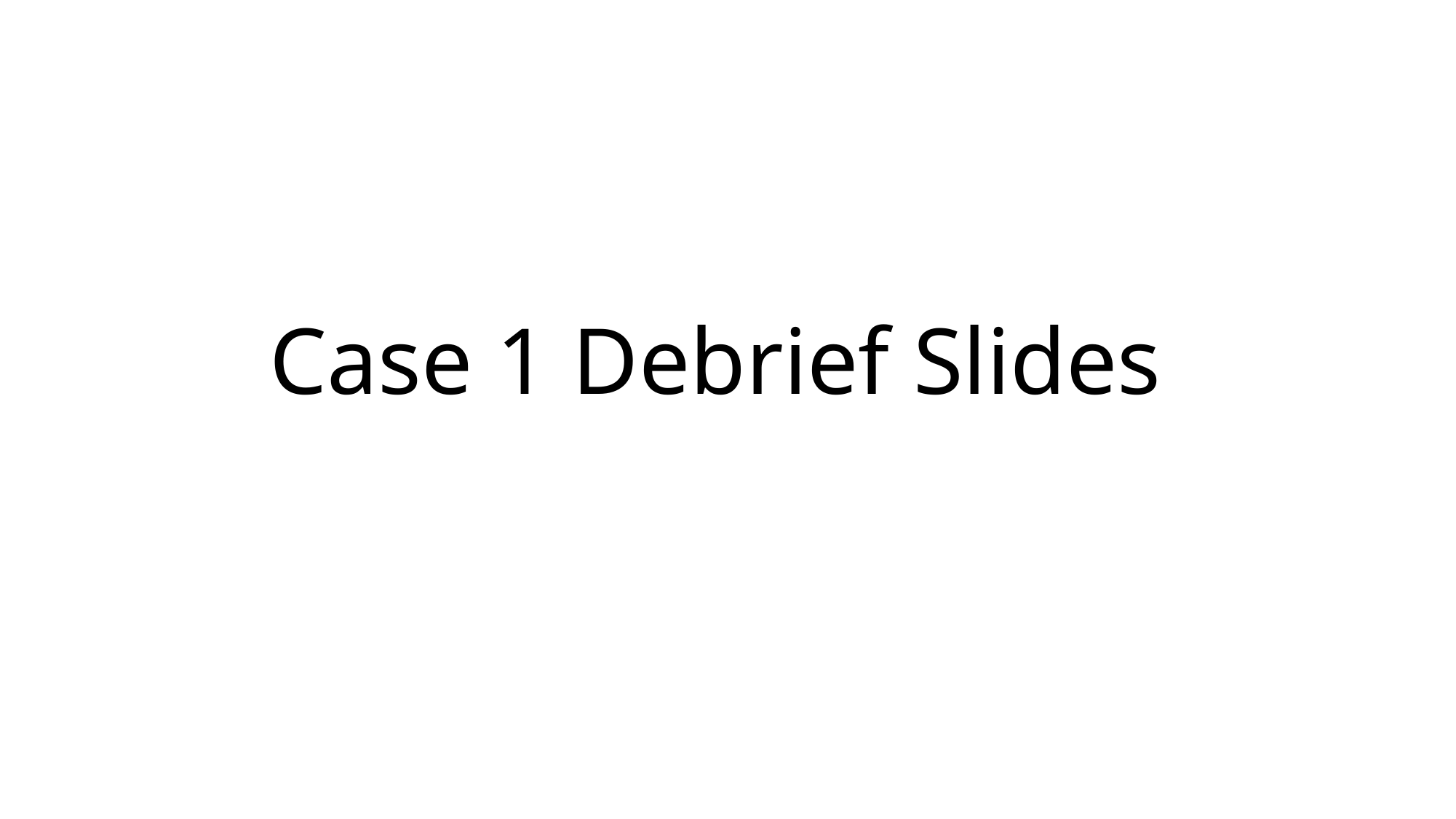

# Case 1 Debrief Slides

## Slide 3
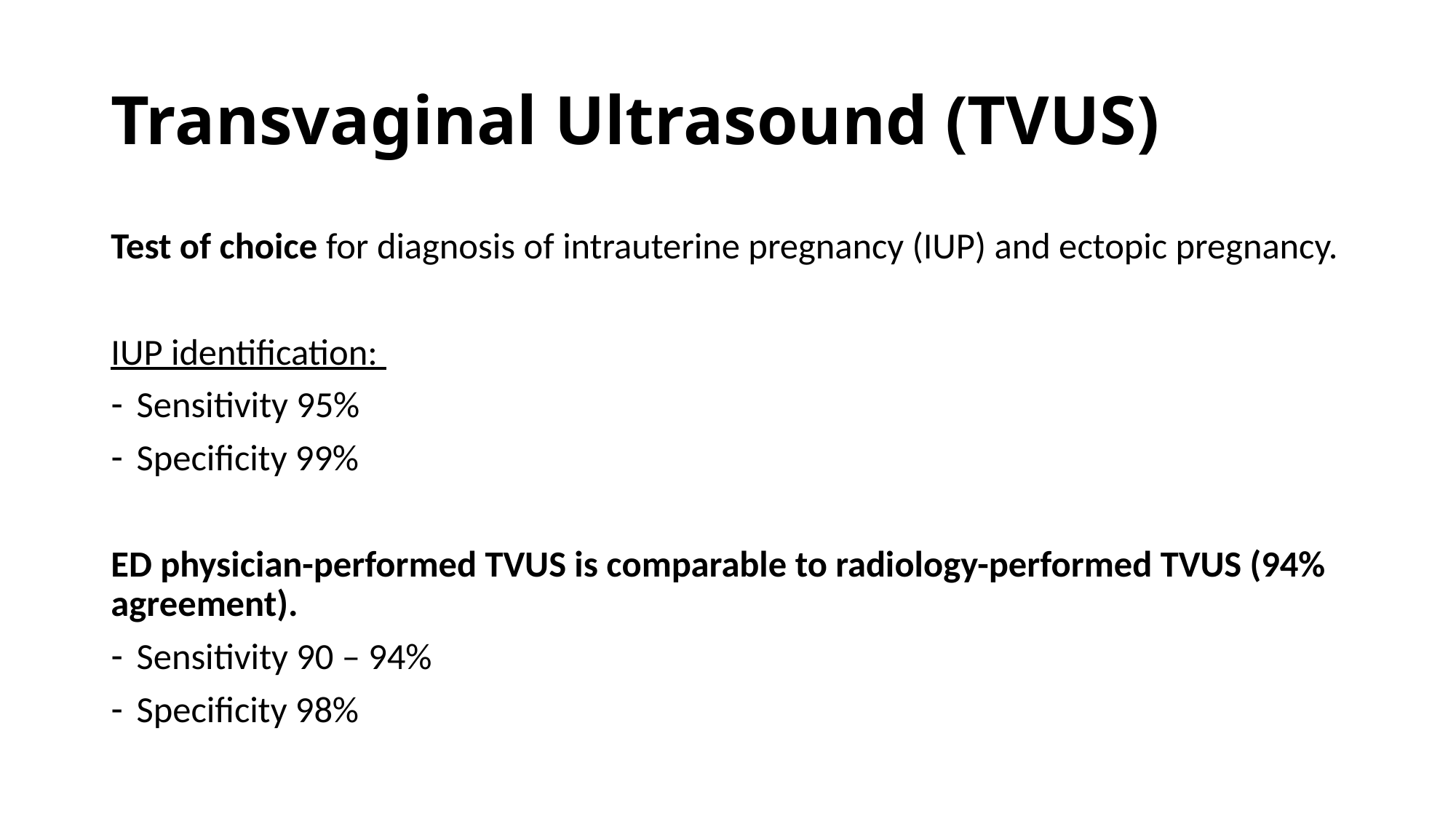

# Transvaginal Ultrasound (TVUS)
Test of choice for diagnosis of intrauterine pregnancy (IUP) and ectopic pregnancy.
IUP identification:
Sensitivity 95%
Specificity 99%
ED physician-performed TVUS is comparable to radiology-performed TVUS (94% agreement).
Sensitivity 90 – 94%
Specificity 98%

## Slide 4
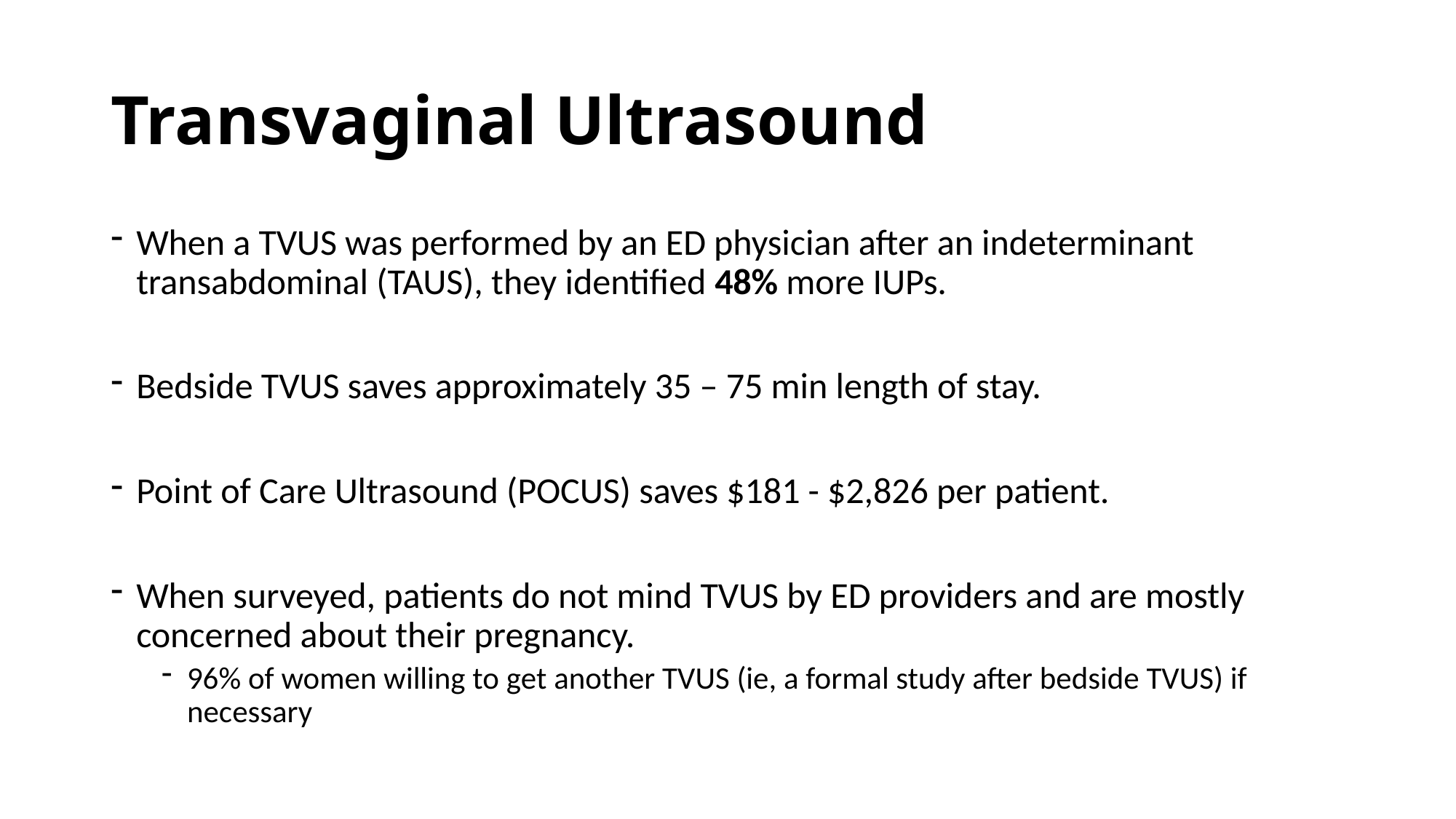

# Transvaginal Ultrasound
When a TVUS was performed by an ED physician after an indeterminant transabdominal (TAUS), they identified 48% more IUPs.
Bedside TVUS saves approximately 35 – 75 min length of stay.
Point of Care Ultrasound (POCUS) saves $181 - $2,826 per patient.
When surveyed, patients do not mind TVUS by ED providers and are mostly concerned about their pregnancy.
96% of women willing to get another TVUS (ie, a formal study after bedside TVUS) if necessary

## Slide 5
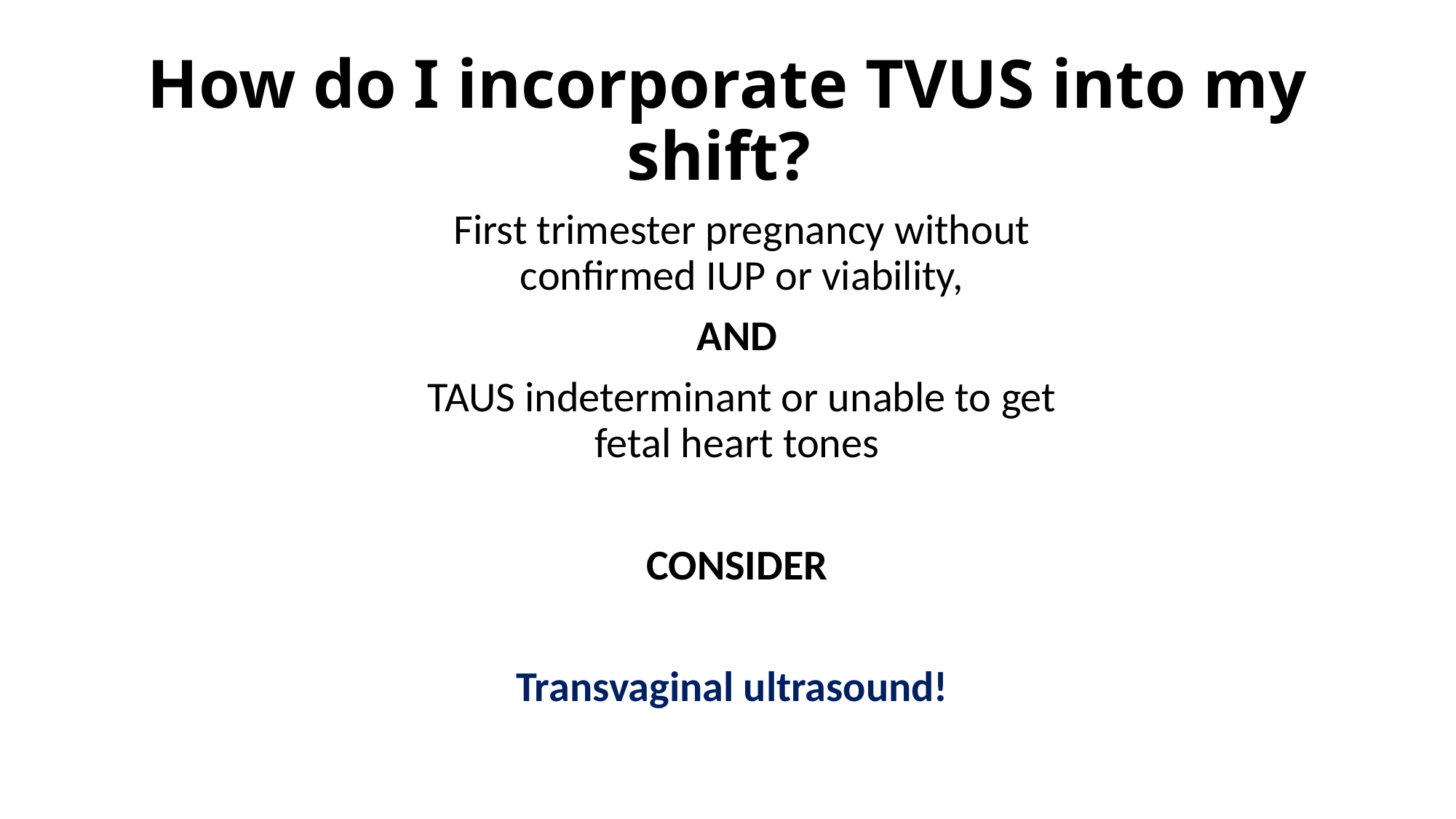

# How do I incorporate TVUS into my shift?
First trimester pregnancy without confirmed IUP or viability,
AND
TAUS indeterminant or unable to get fetal heart tones
CONSIDER
Transvaginal ultrasound!

## Slide 6
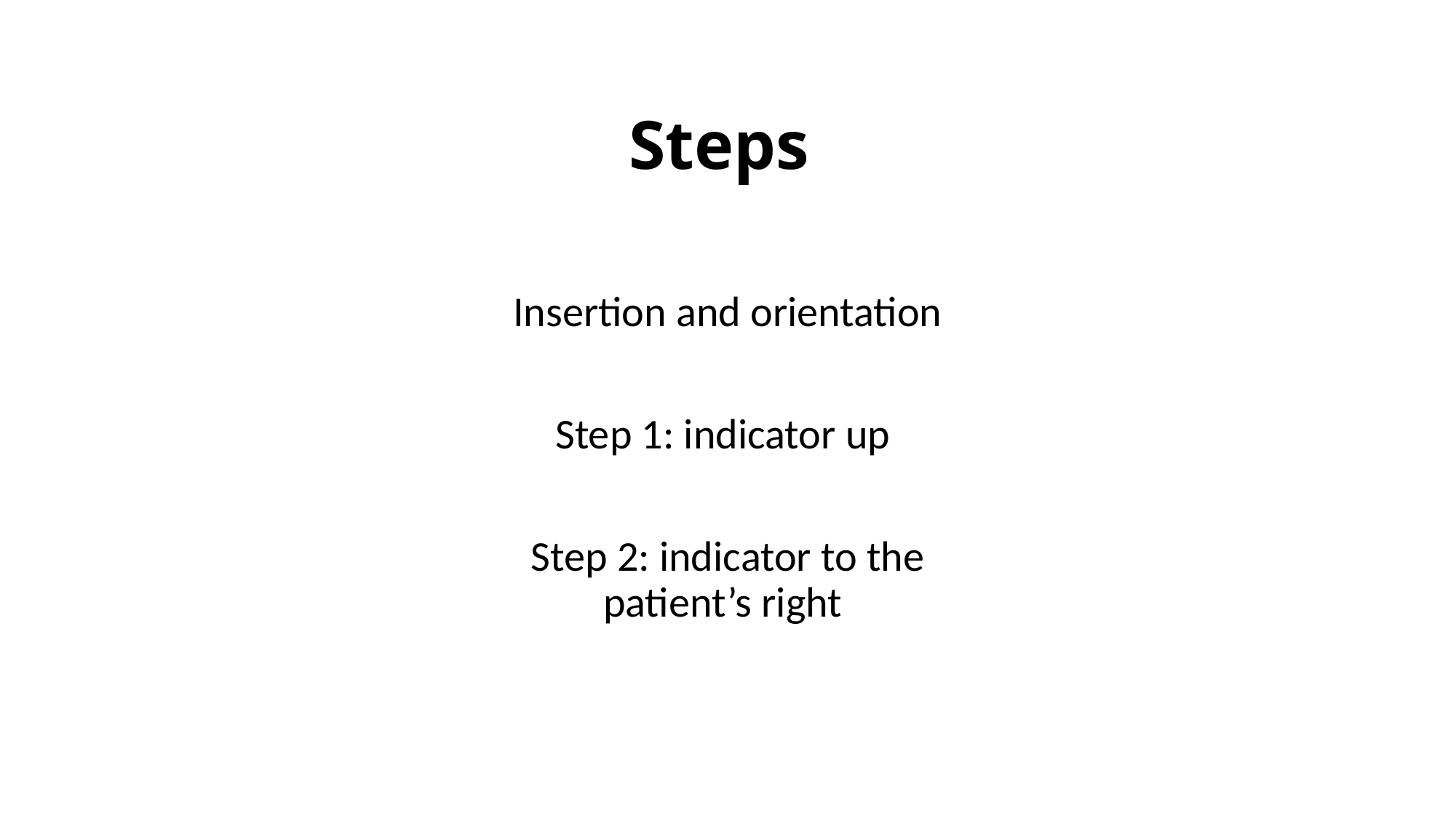

# Steps
Insertion and orientation
Step 1: indicator up
Step 2: indicator to the patient’s right

## Slide 7
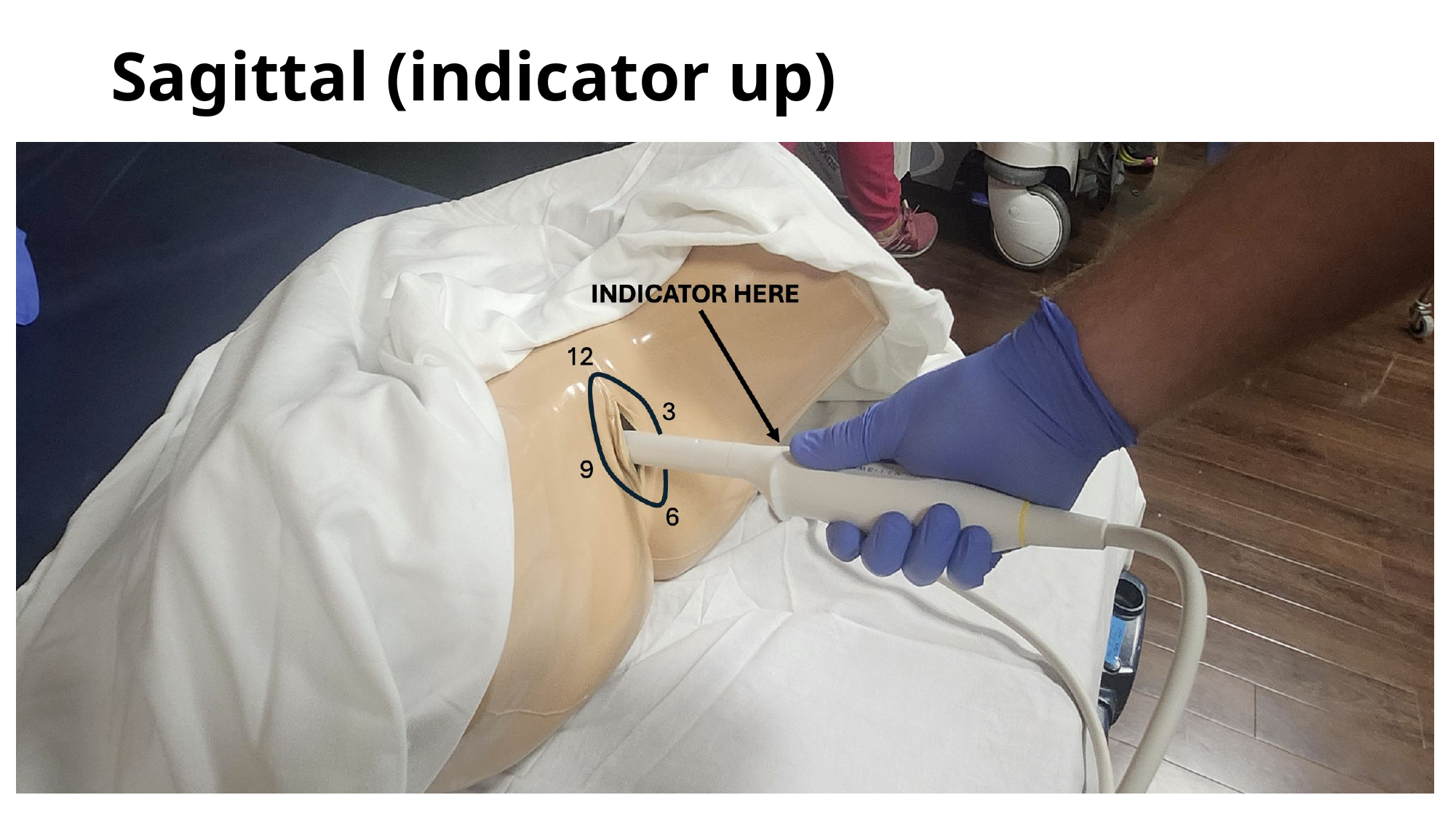

# Sagittal (indicator up)

## Slide 8
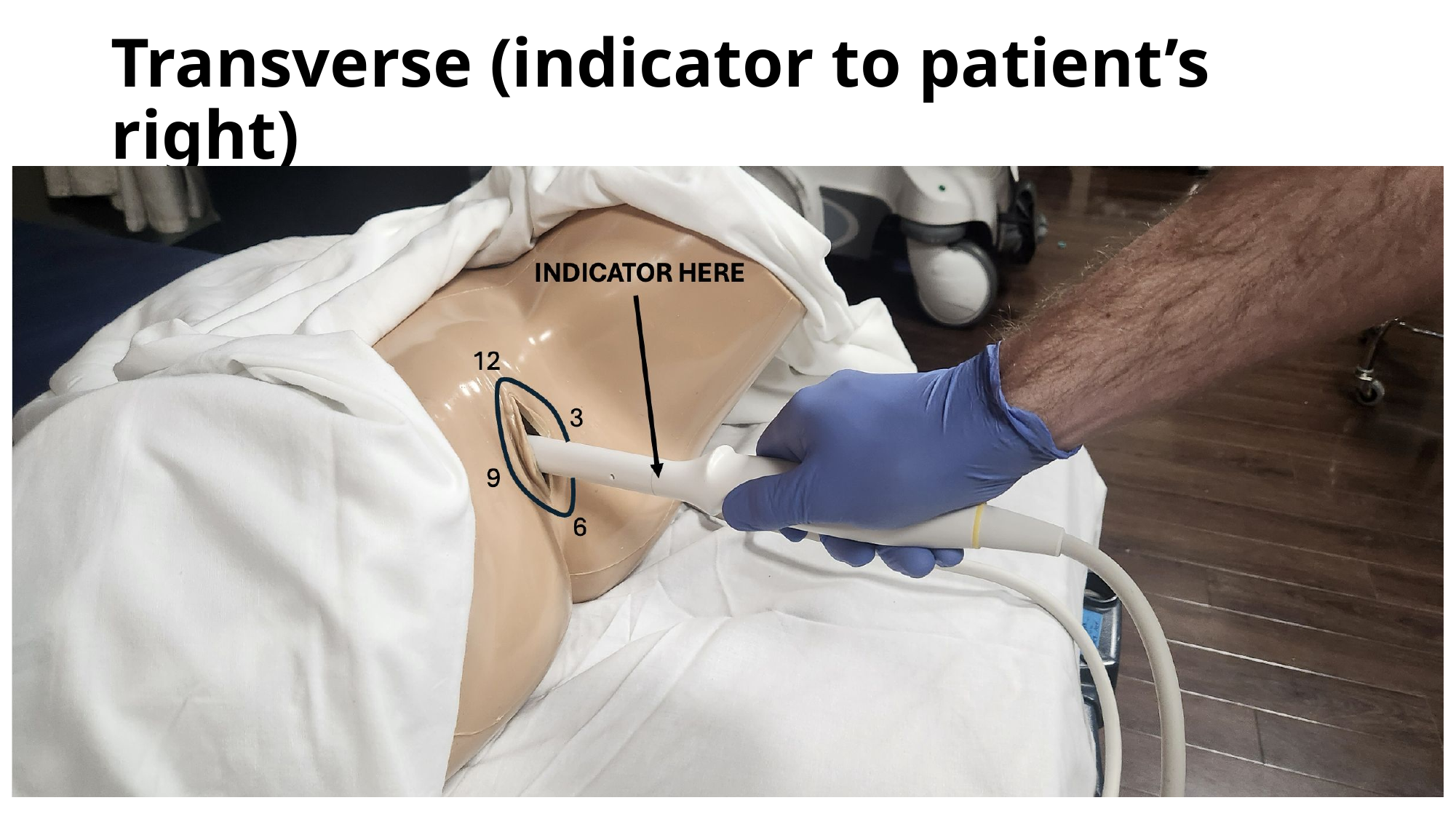

# Transverse (indicator to patient’s right)

## Slide 9
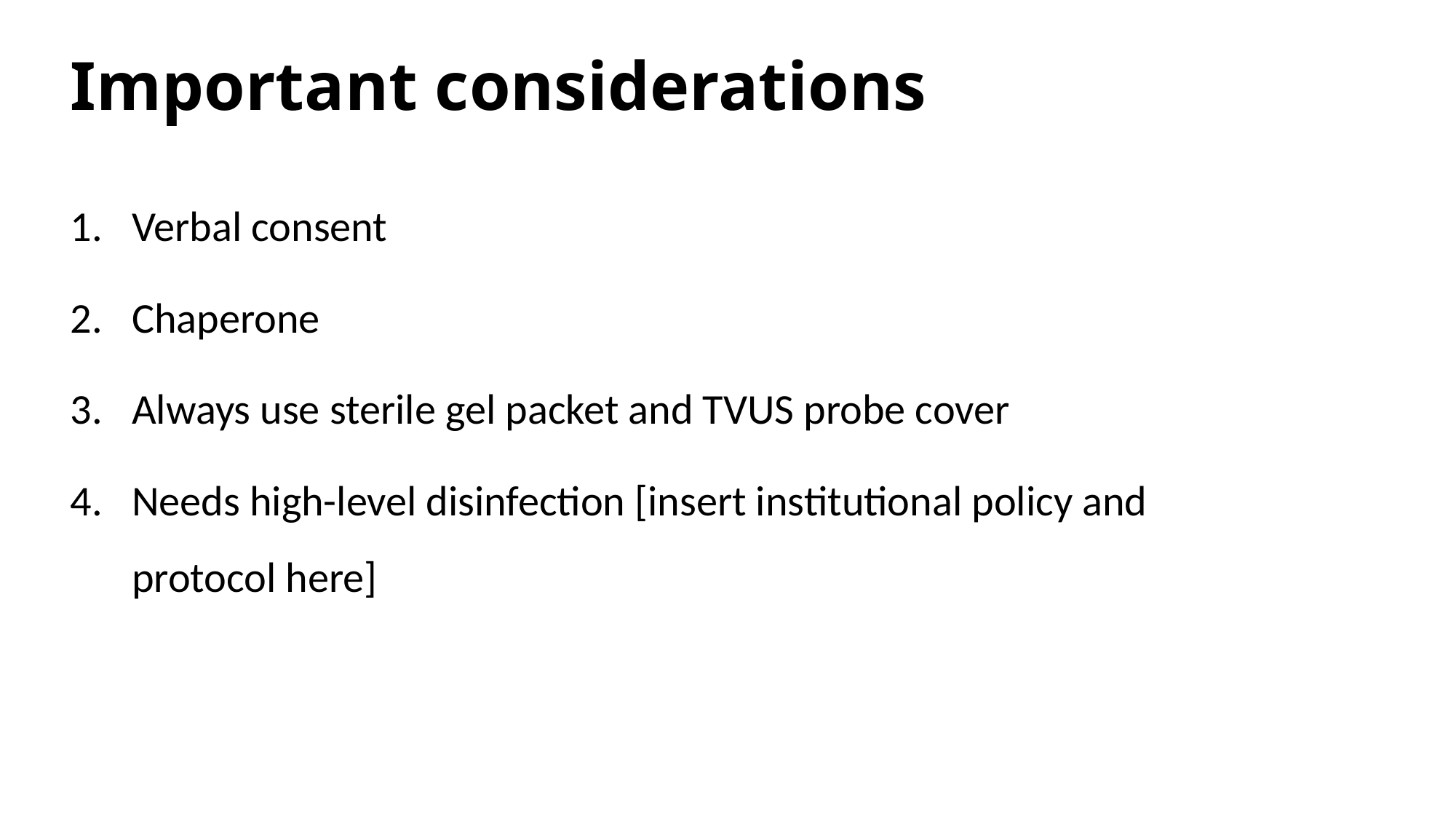

# Important considerations
Verbal consent
Chaperone
Always use sterile gel packet and TVUS probe cover
Needs high-level disinfection [insert institutional policy and protocol here]

## Slide 10
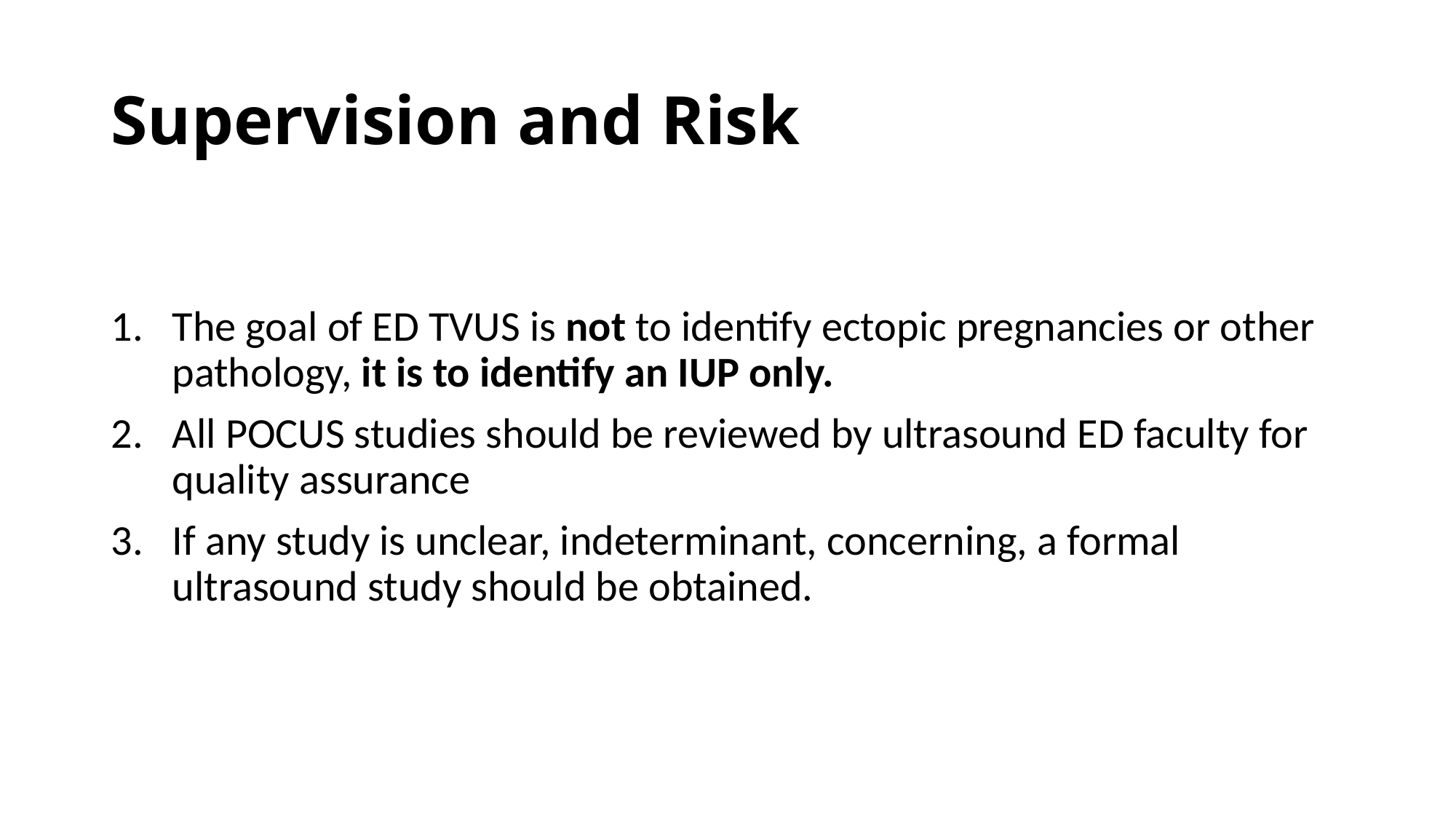

# Supervision and Risk
The goal of ED TVUS is not to identify ectopic pregnancies or other pathology, it is to identify an IUP only.
All POCUS studies should be reviewed by ultrasound ED faculty for quality assurance
If any study is unclear, indeterminant, concerning, a formal ultrasound study should be obtained.

## Slide 11
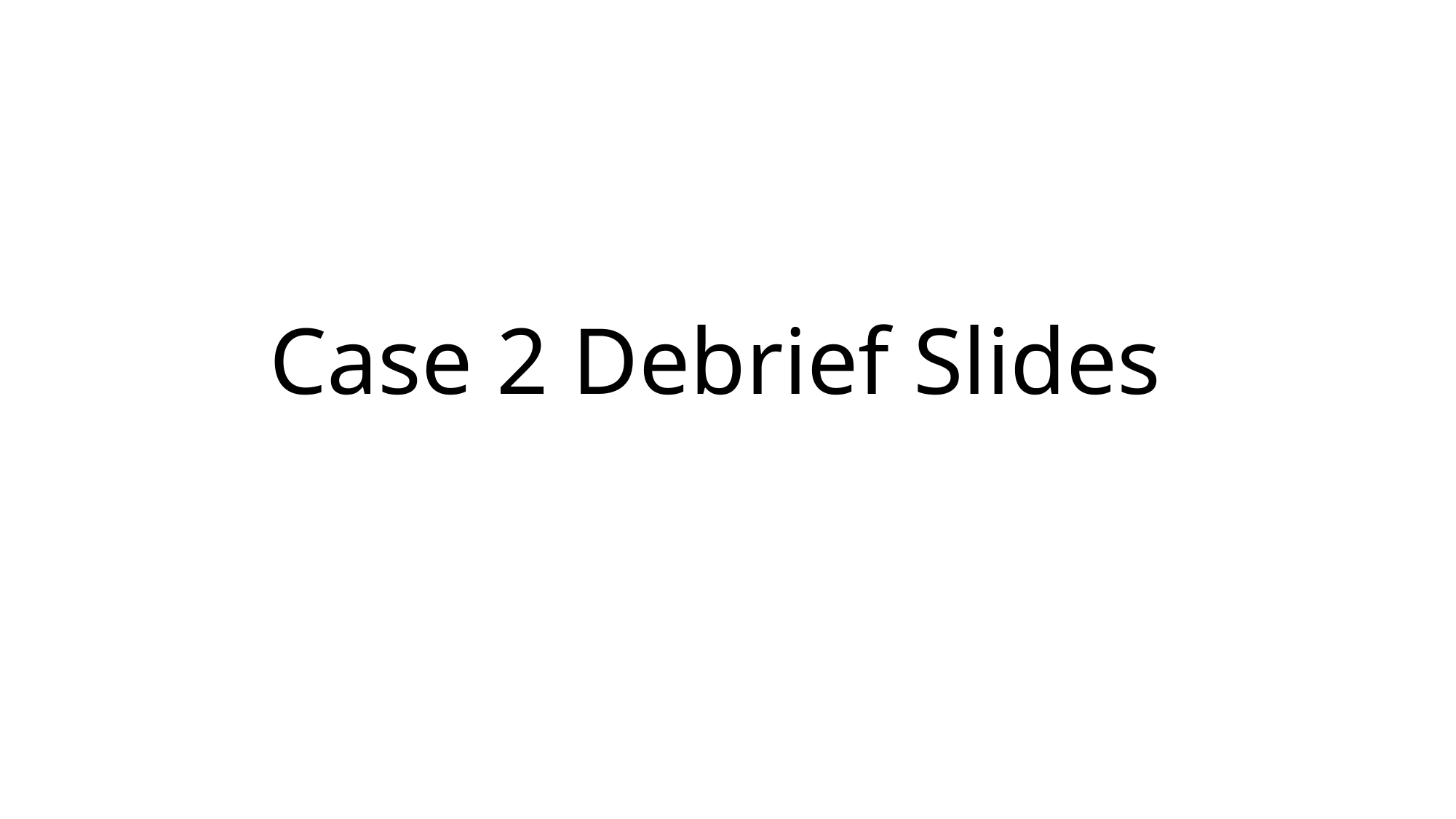

# Case 2 Debrief Slides

## Slide 12
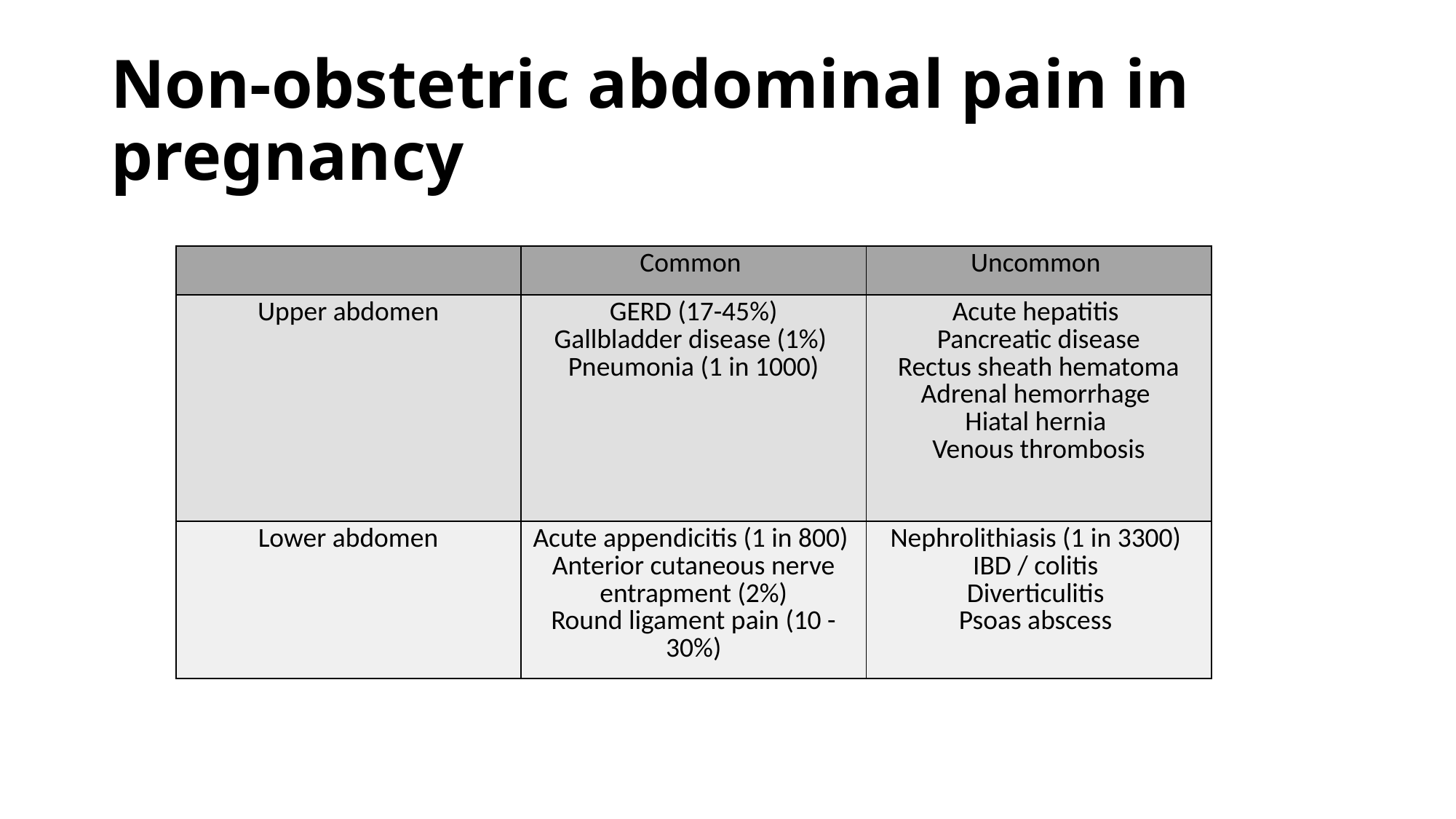

# Non-obstetric abdominal pain in pregnancy
| | Common | Uncommon |
| --- | --- | --- |
| Upper abdomen | GERD (17-45%) Gallbladder disease (1%) Pneumonia (1 in 1000) | Acute hepatitis Pancreatic disease Rectus sheath hematoma Adrenal hemorrhage Hiatal hernia Venous thrombosis |
| Lower abdomen | Acute appendicitis (1 in 800) Anterior cutaneous nerve entrapment (2%) Round ligament pain (10 -30%) | Nephrolithiasis (1 in 3300) IBD / colitis Diverticulitis Psoas abscess |

## Slide 13
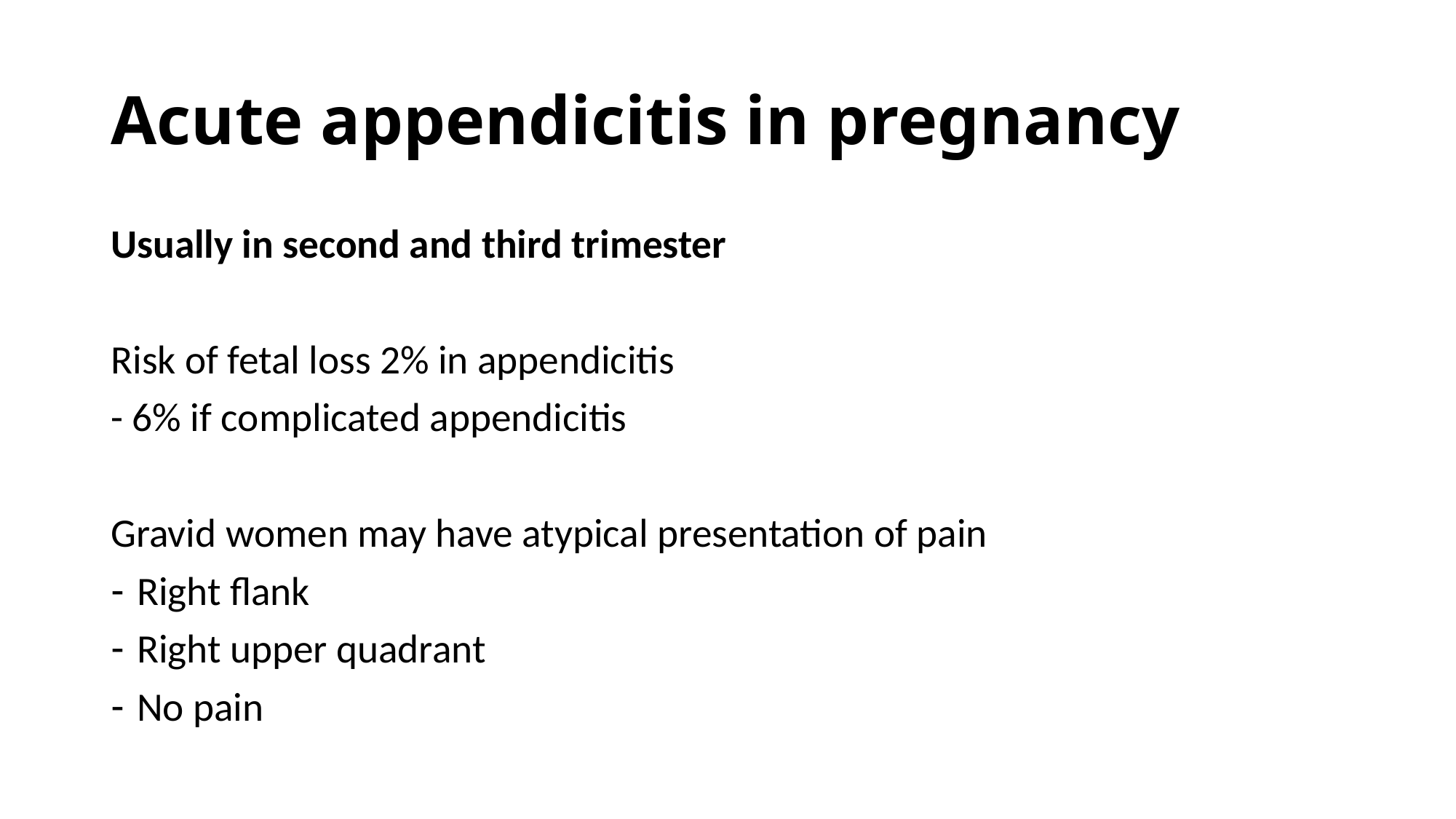

# Acute appendicitis in pregnancy
Usually in second and third trimester
Risk of fetal loss 2% in appendicitis
- 6% if complicated appendicitis
Gravid women may have atypical presentation of pain
Right flank
Right upper quadrant
No pain

## Slide 14
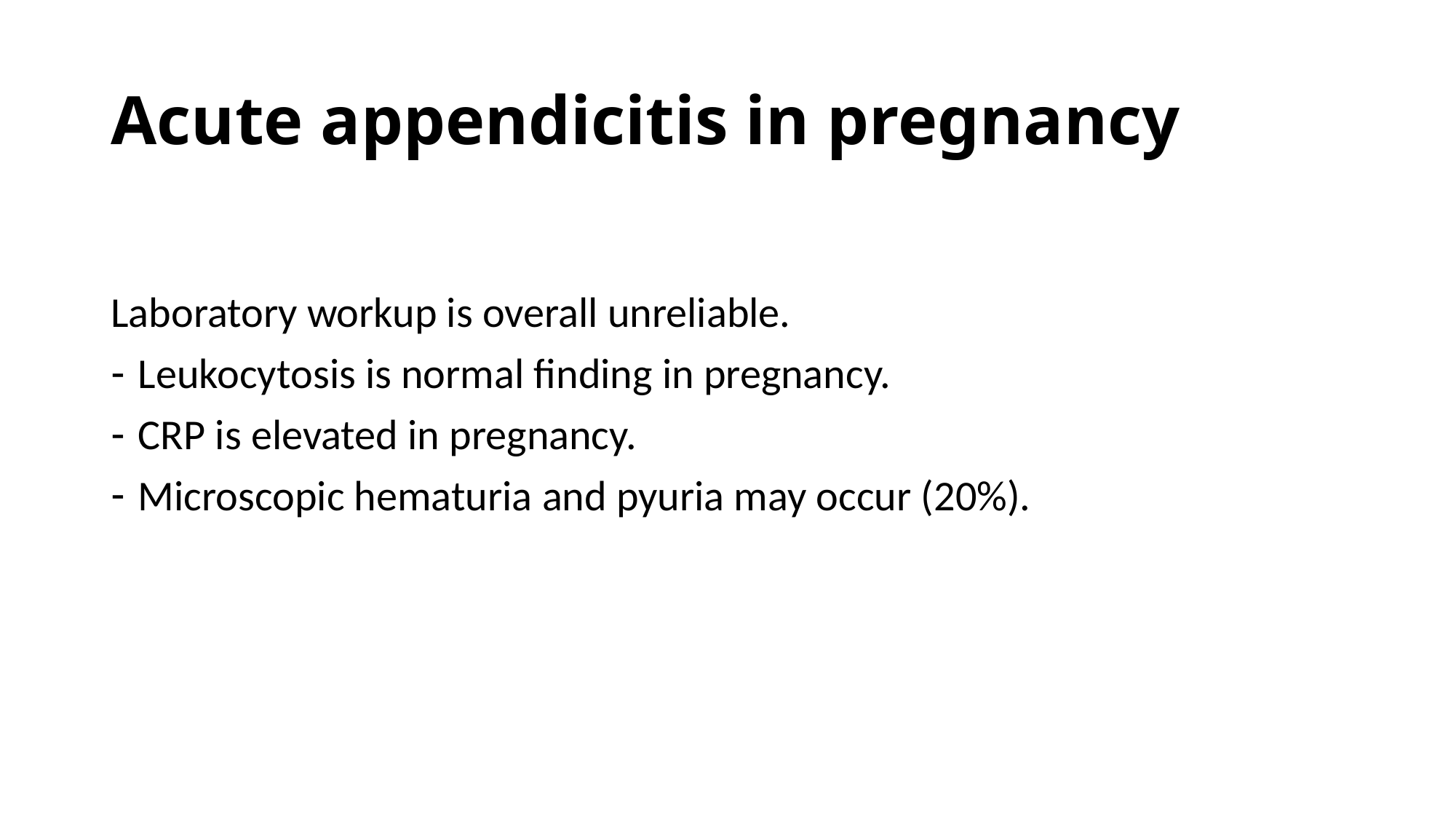

# Acute appendicitis in pregnancy
Laboratory workup is overall unreliable.
Leukocytosis is normal finding in pregnancy.
CRP is elevated in pregnancy.
Microscopic hematuria and pyuria may occur (20%).

## Slide 15
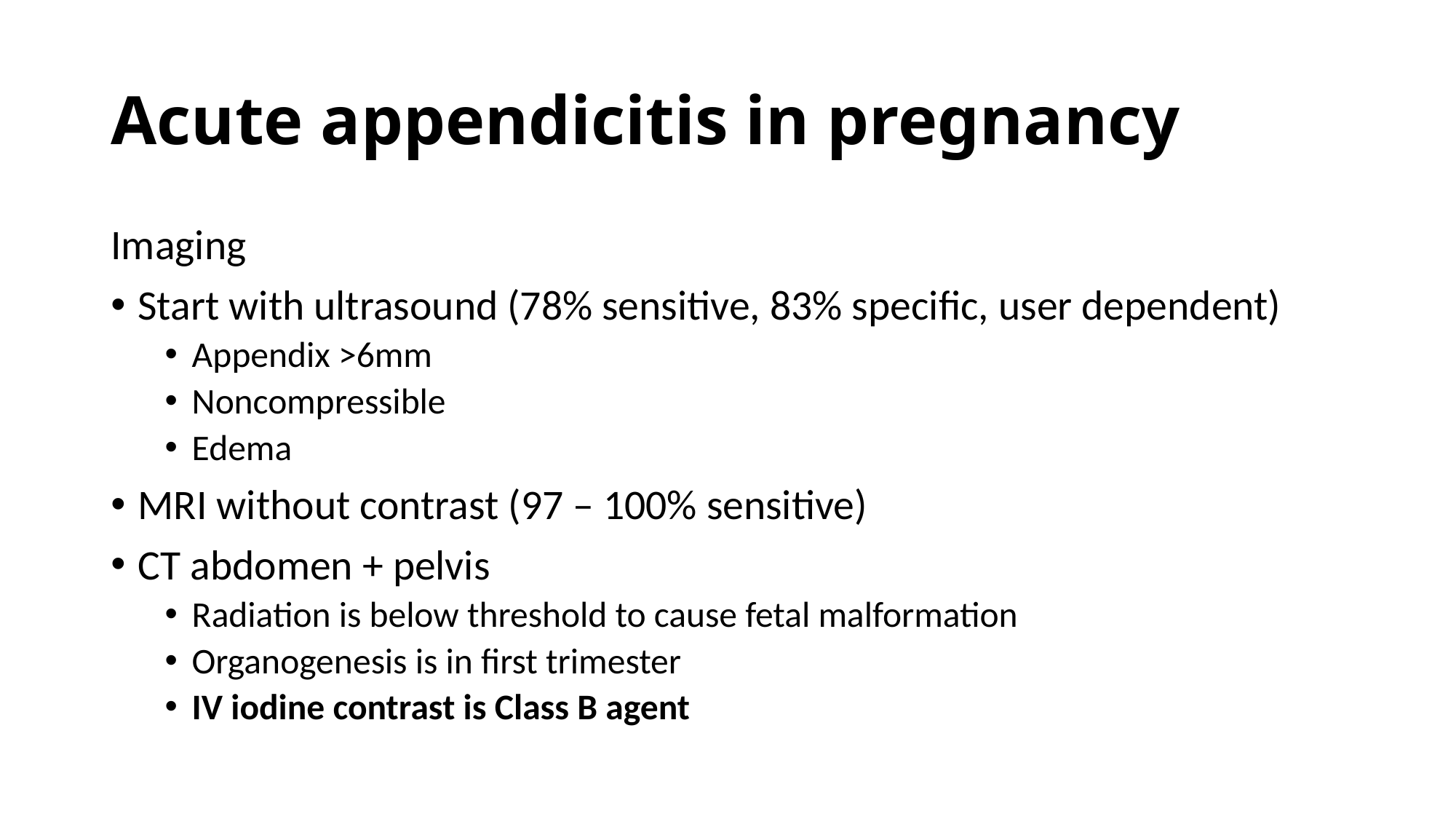

# Acute appendicitis in pregnancy
Imaging
Start with ultrasound (78% sensitive, 83% specific, user dependent)
Appendix >6mm
Noncompressible
Edema
MRI without contrast (97 – 100% sensitive)
CT abdomen + pelvis
Radiation is below threshold to cause fetal malformation
Organogenesis is in first trimester
IV iodine contrast is Class B agent

## Slide 16
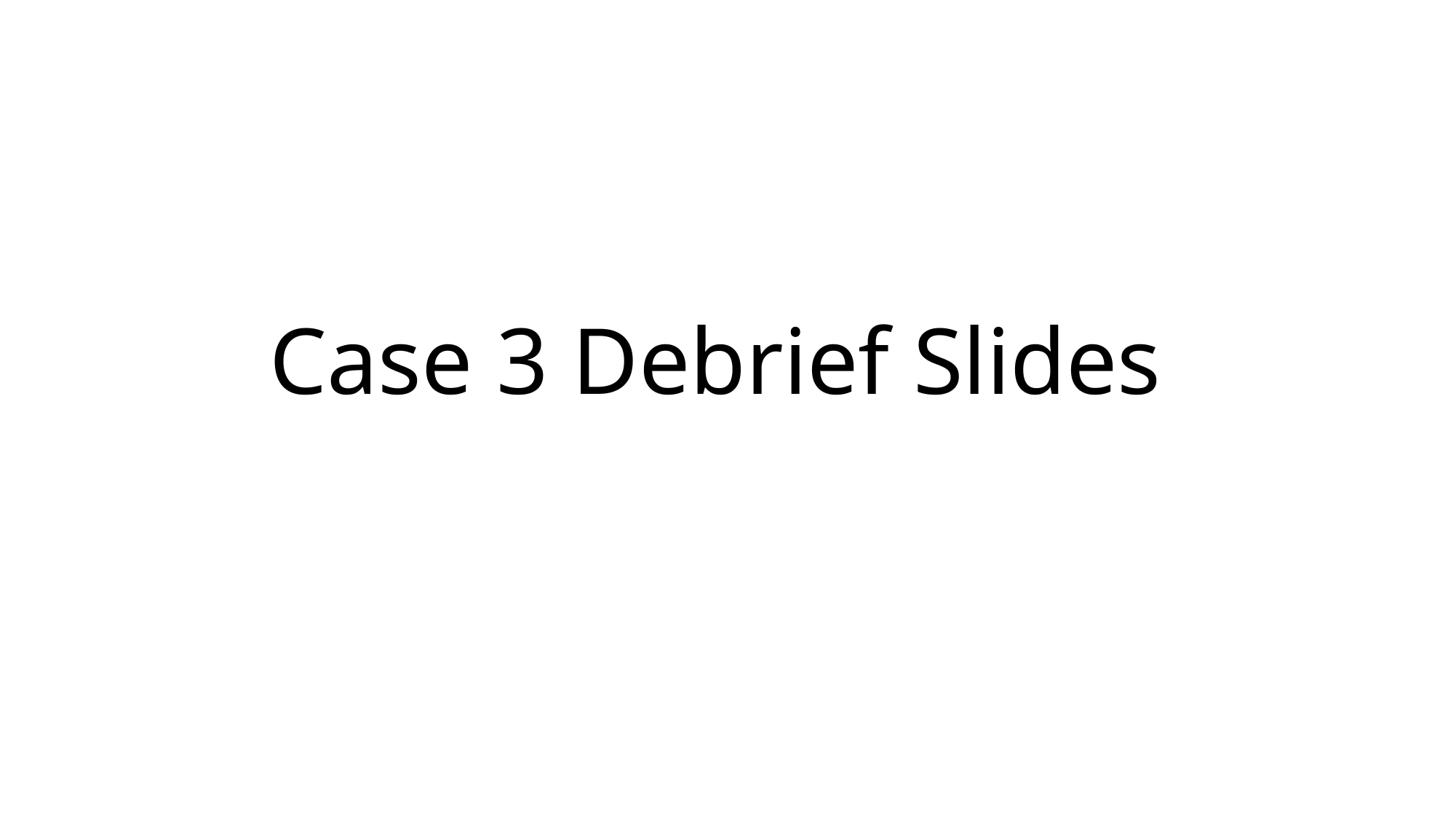

# Case 3 Debrief Slides

## Slide 17
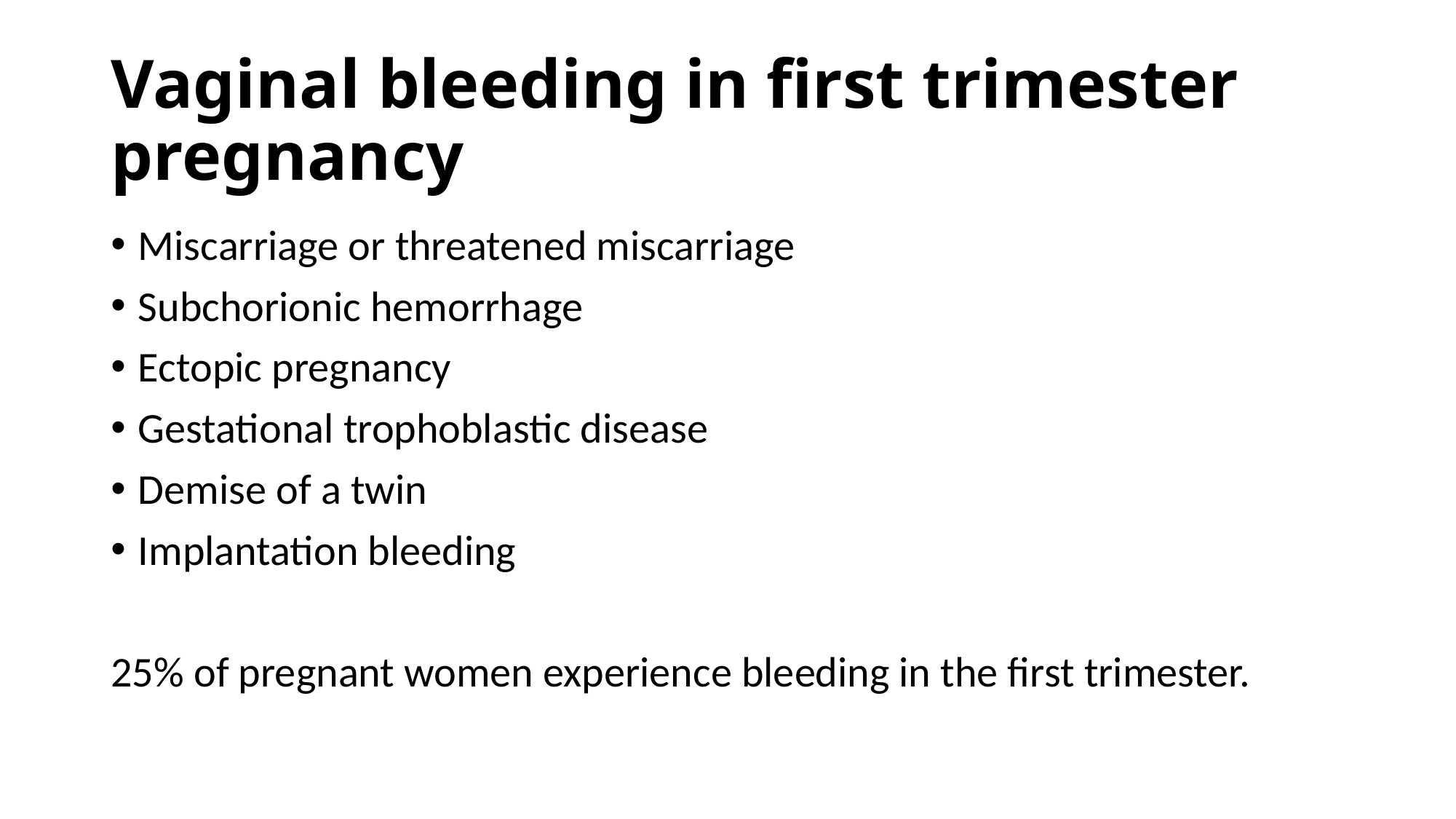

# Vaginal bleeding in first trimester pregnancy
Miscarriage or threatened miscarriage
Subchorionic hemorrhage
Ectopic pregnancy
Gestational trophoblastic disease
Demise of a twin
Implantation bleeding
25% of pregnant women experience bleeding in the first trimester.

## Slide 18
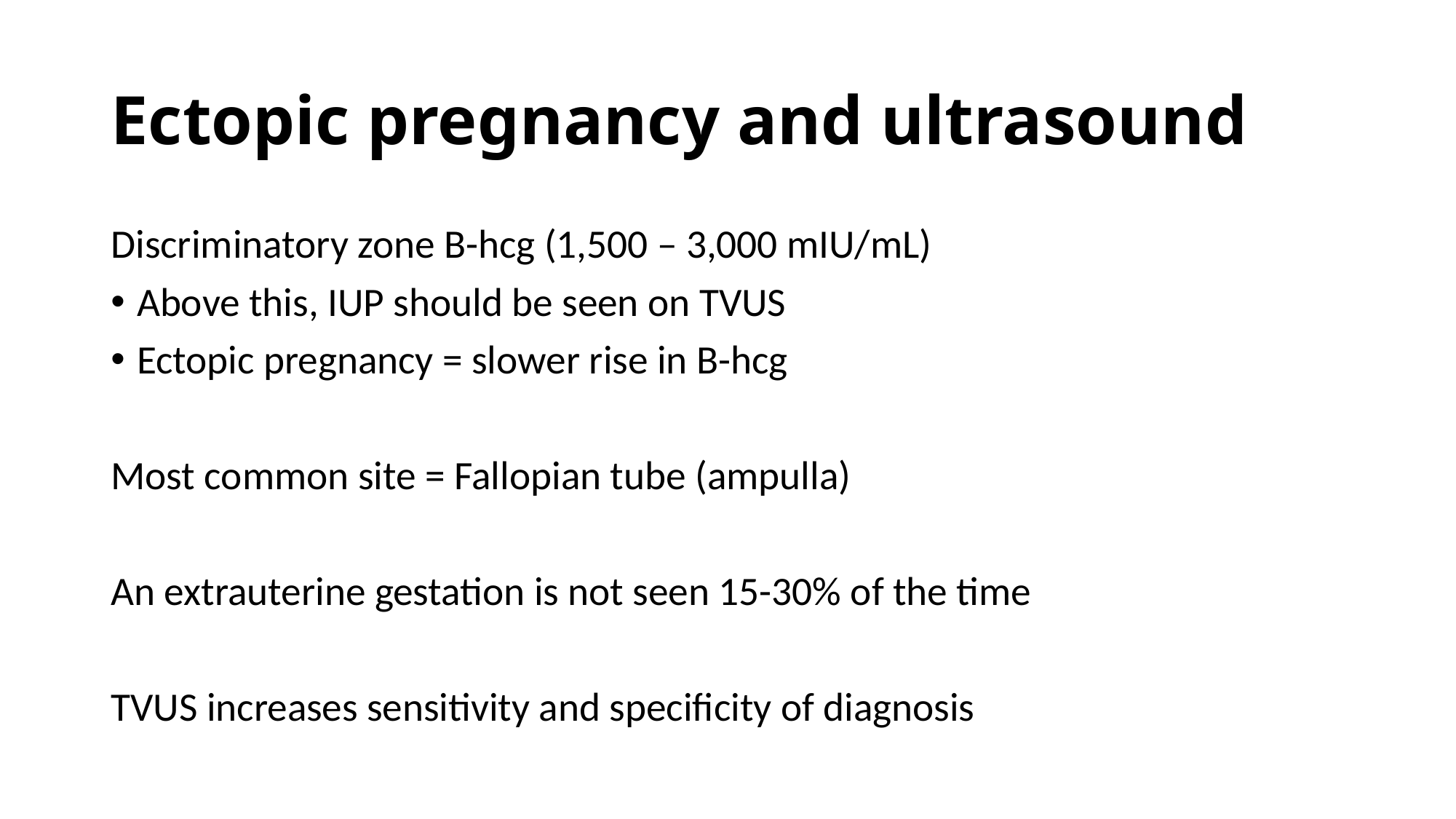

# Ectopic pregnancy and ultrasound
Discriminatory zone B-hcg (1,500 – 3,000 mIU/mL)
Above this, IUP should be seen on TVUS
Ectopic pregnancy = slower rise in B-hcg
Most common site = Fallopian tube (ampulla)
An extrauterine gestation is not seen 15-30% of the time
TVUS increases sensitivity and specificity of diagnosis

## Slide 19
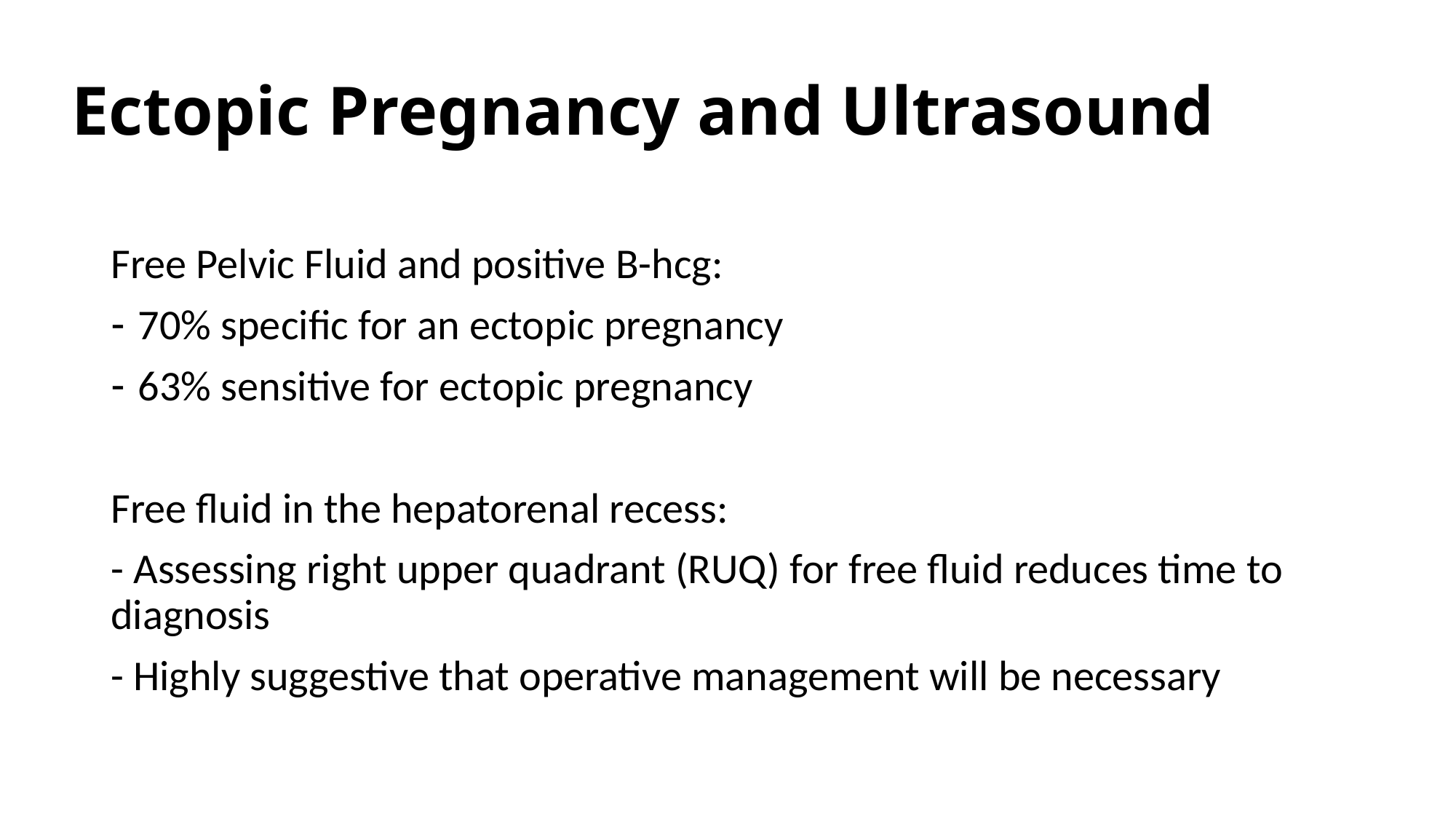

# Ectopic Pregnancy and Ultrasound
Free Pelvic Fluid and positive B-hcg:
70% specific for an ectopic pregnancy
63% sensitive for ectopic pregnancy
Free fluid in the hepatorenal recess:
- Assessing right upper quadrant (RUQ) for free fluid reduces time to diagnosis
- Highly suggestive that operative management will be necessary

## Slide 20
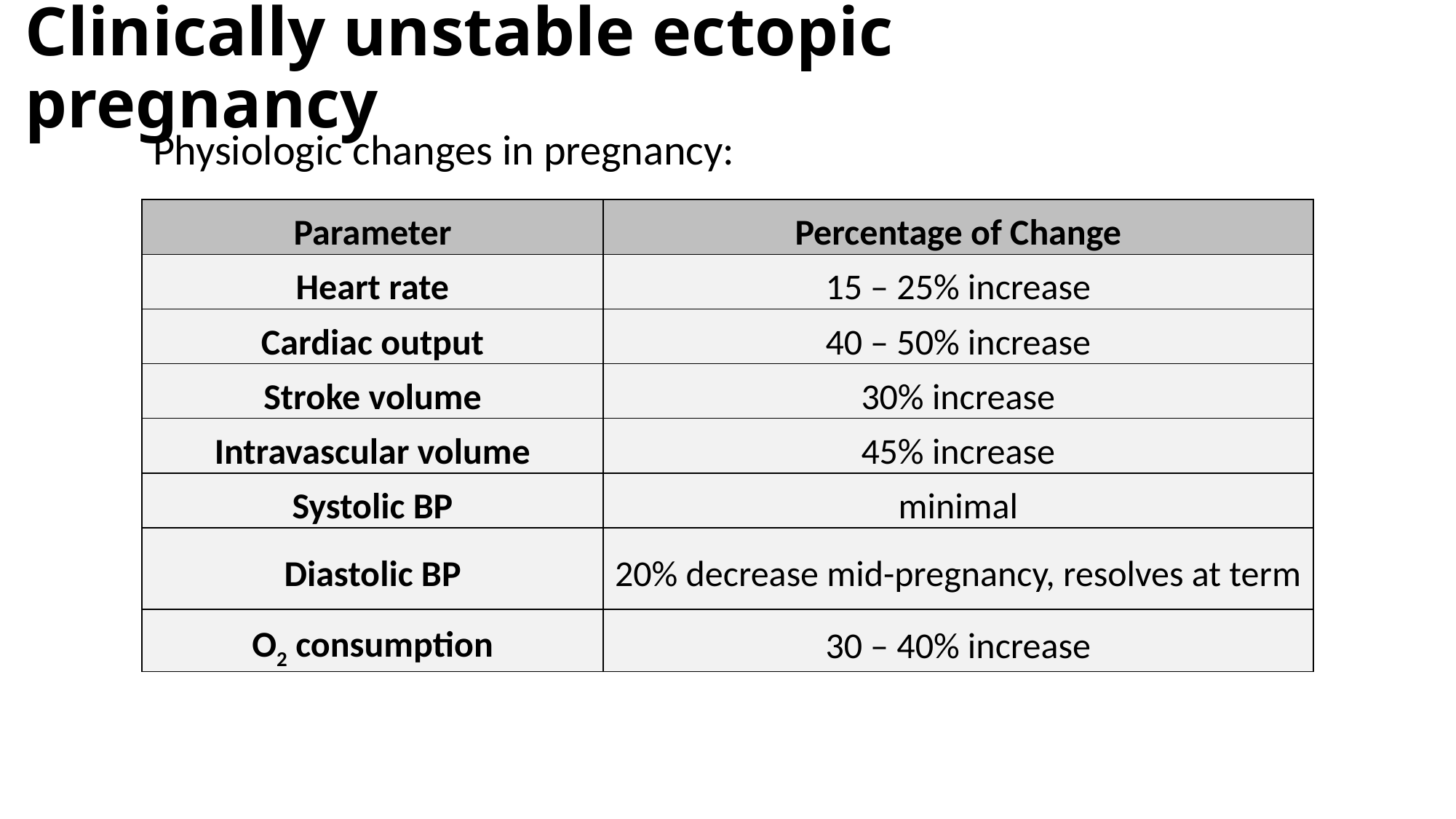

# Clinically unstable ectopic pregnancy
Physiologic changes in pregnancy:
| Parameter | Percentage of Change |
| --- | --- |
| Heart rate | 15 – 25% increase |
| Cardiac output | 40 – 50% increase |
| Stroke volume | 30% increase |
| Intravascular volume | 45% increase |
| Systolic BP | minimal |
| Diastolic BP | 20% decrease mid-pregnancy, resolves at term |
| O2 consumption | 30 – 40% increase |

## Slide 21
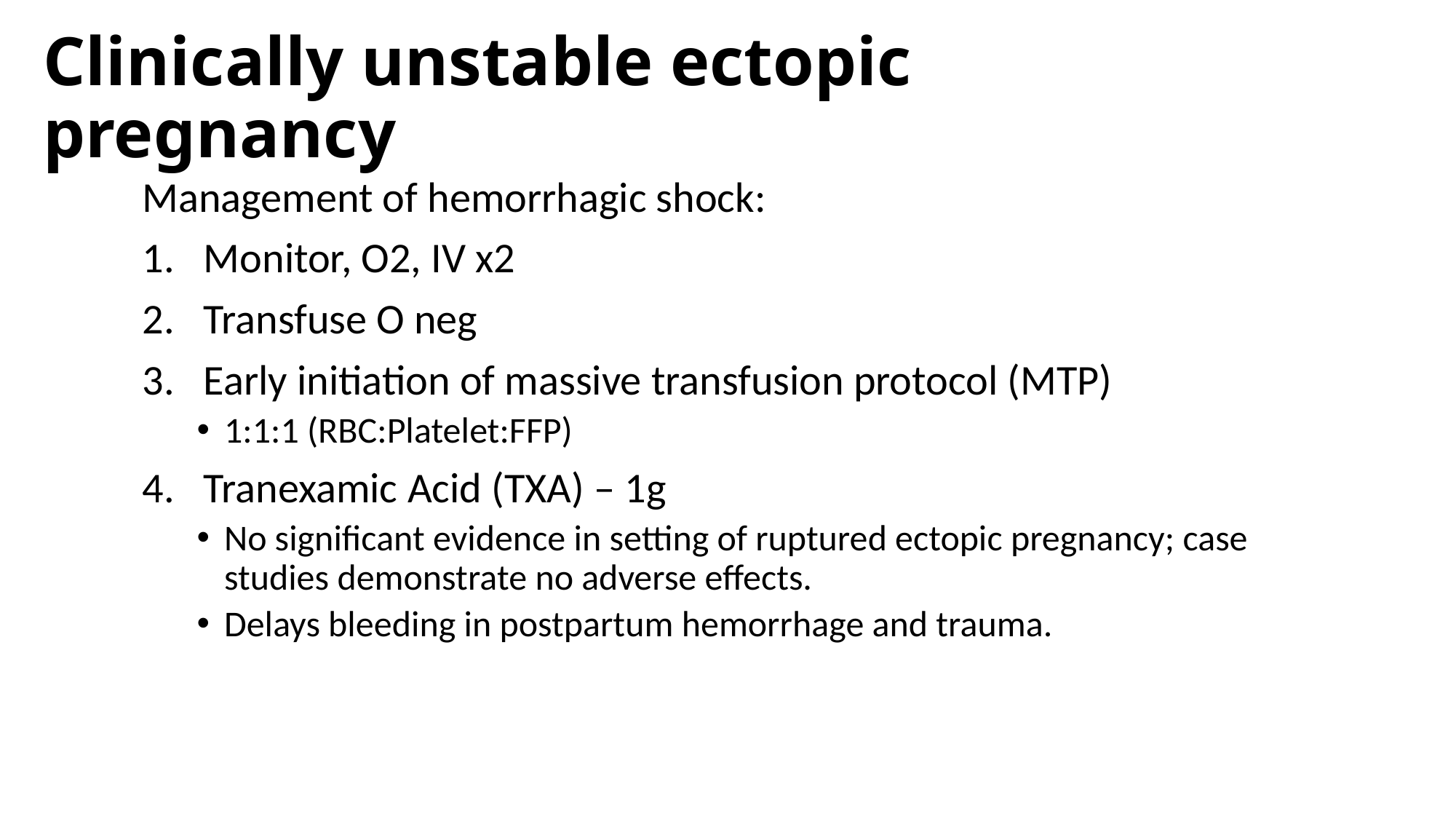

# Clinically unstable ectopic pregnancy
Management of hemorrhagic shock:
Monitor, O2, IV x2
Transfuse O neg
Early initiation of massive transfusion protocol (MTP)
1:1:1 (RBC:Platelet:FFP)
Tranexamic Acid (TXA) – 1g
No significant evidence in setting of ruptured ectopic pregnancy; case studies demonstrate no adverse effects.
Delays bleeding in postpartum hemorrhage and trauma.
