# Supplementary material for: High-Fidelity Simulation with Transvaginal Ultrasound in the Emergency Department
Source: J Educ Teach Emerg Med. 2024 Jul 31;9(3):S65–S135. doi: 10.21980/J8606Q (PMC11312878; doi:10.21980/J8606Q)
Supplement: Supplementary file 2 [file 9-3-S65-Supp2.pptx]

## Slide 1
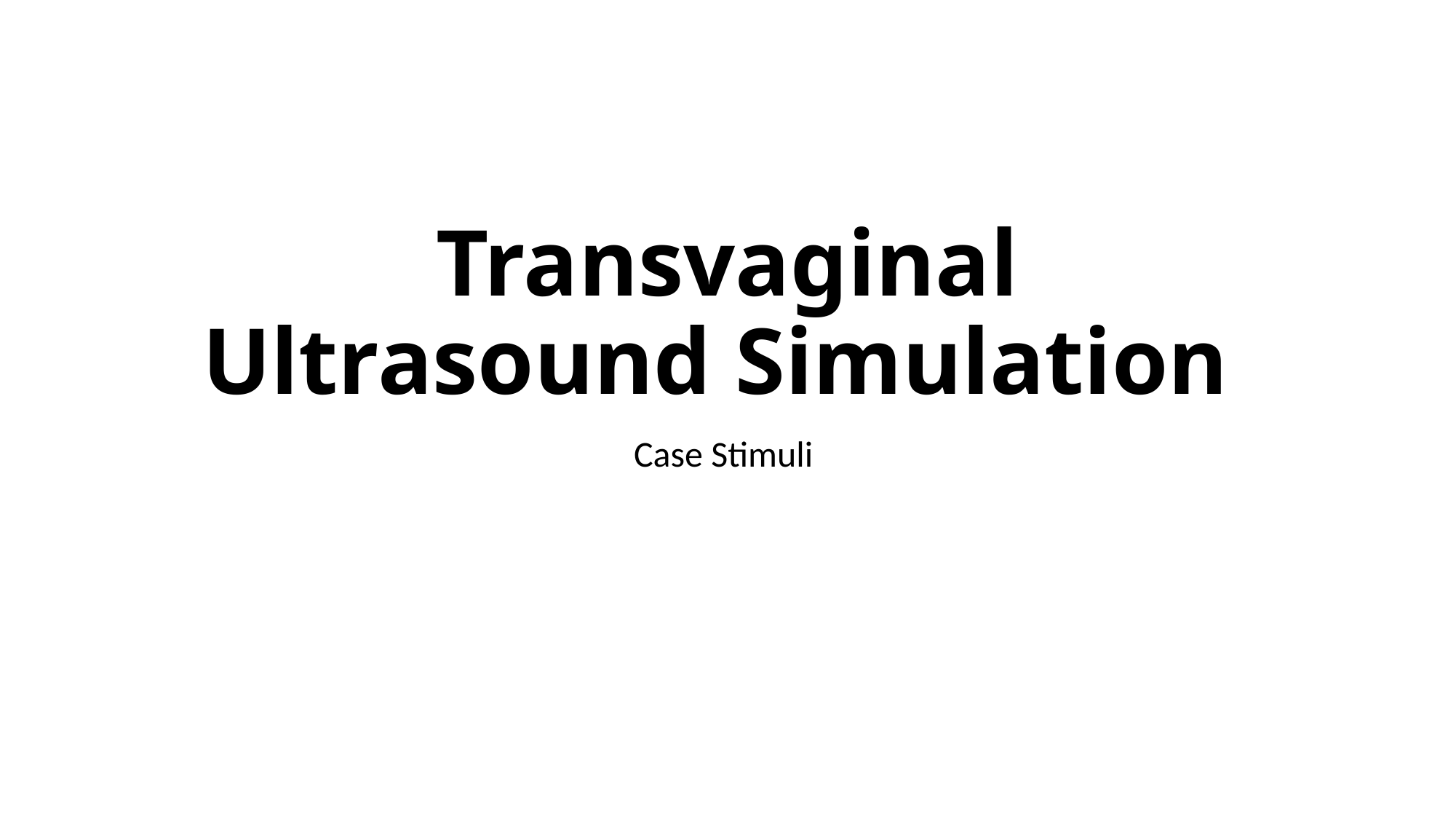

# Transvaginal Ultrasound Simulation
Case Stimuli

## Slide 2
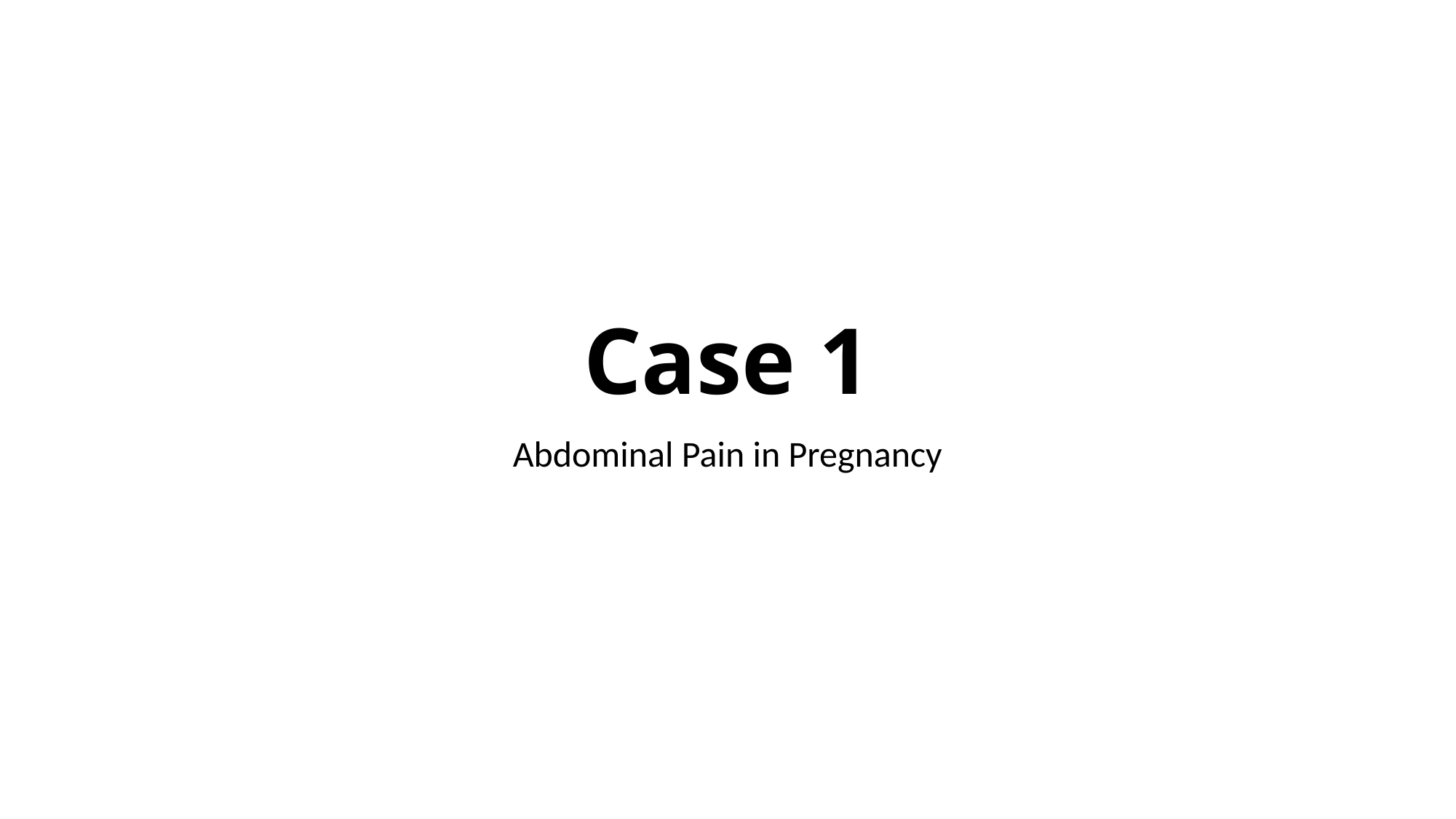

# Case 1
Abdominal Pain in Pregnancy

## Slide 3
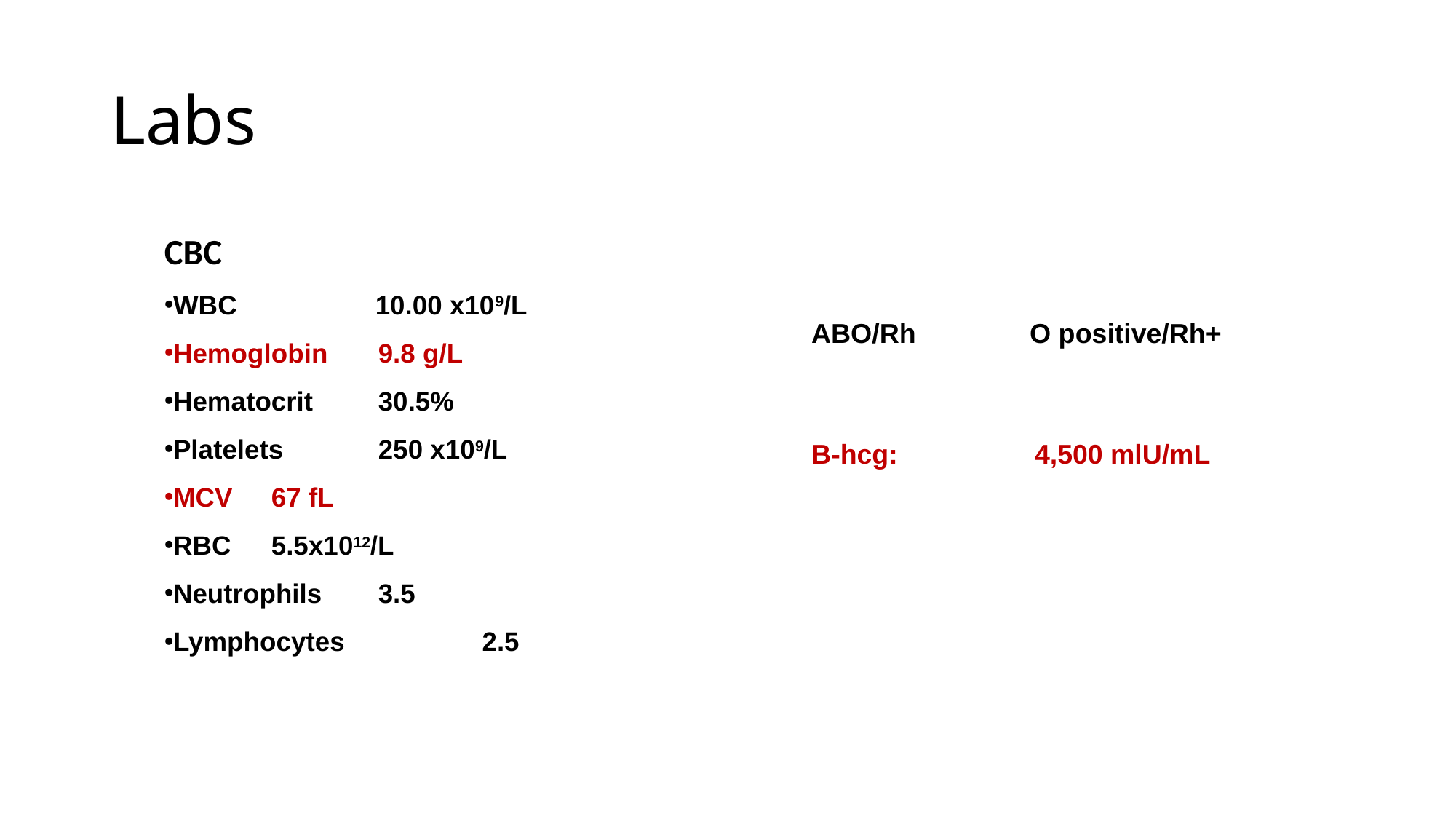

# Labs
CBC
WBC		 10.00 x109/L
Hemoglobin 		9.8 g/L
Hematocrit 		30.5%
Platelets 		250 x109/L
MCV			67 fL
RBC			5.5x10^12^/L
Neutrophils 		3.5
Lymphocytes 	 2.5
ABO/Rh 	O positive/Rh+
B-hcg:	 4,500 mlU/mL

## Slide 4
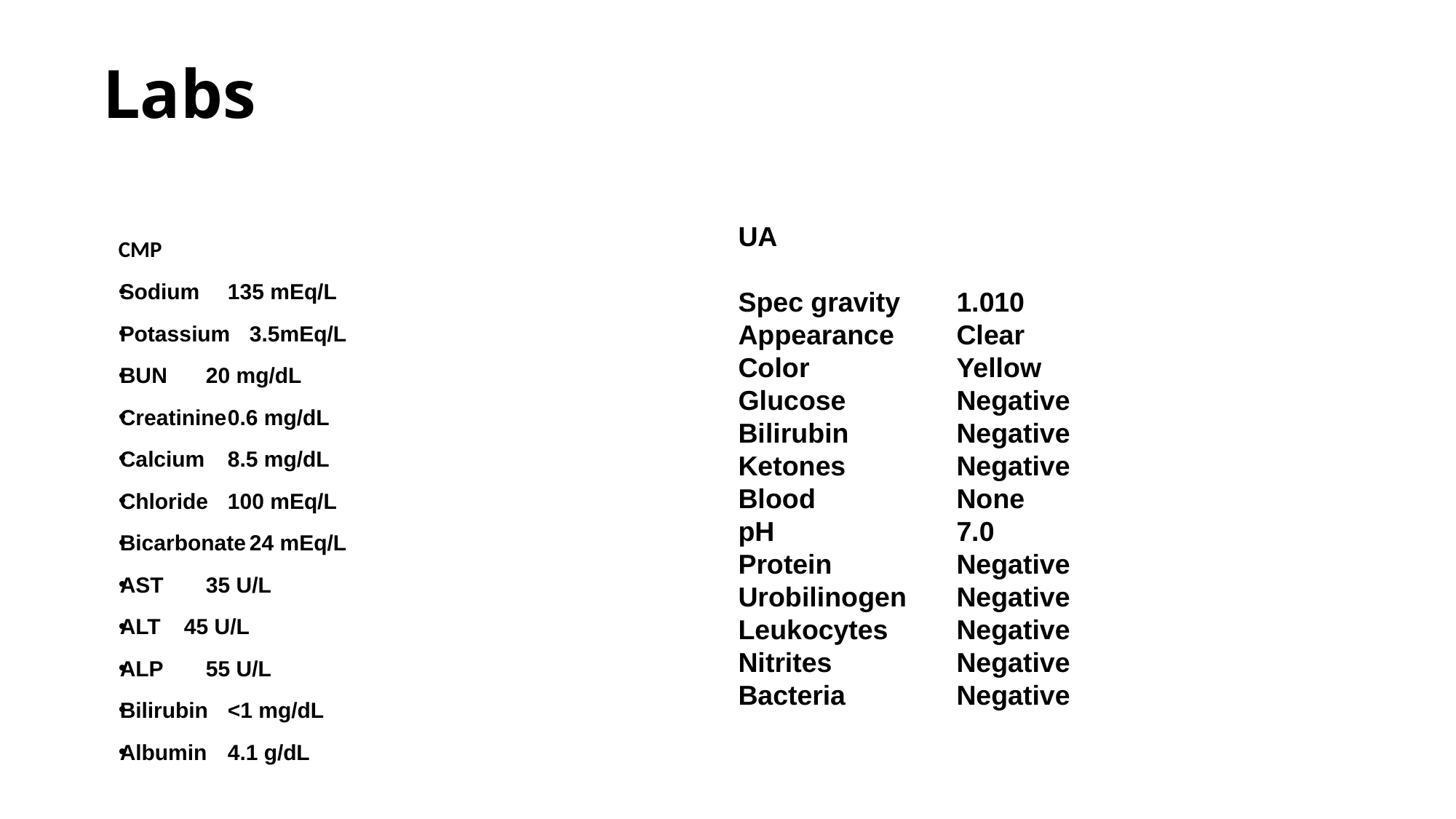

# Labs
CMP
Sodium 	135 mEq/L
Potassium 	3.5mEq/L
BUN		20 mg/dL
Creatinine	0.6 mg/dL
Calcium 	8.5 mg/dL
Chloride 	100 mEq/L
Bicarbonate	24 mEq/L
AST		35 U/L
ALT		45 U/L
ALP 		55 U/L
Bilirubin 	<1 mg/dL
Albumin 	4.1 g/dL
UA
Spec gravity 	1.010
Appearance	Clear
Color		Yellow
Glucose		Negative
Bilirubin 	Negative
Ketones		Negative
Blood 		None
pH		7.0
Protein		Negative
Urobilinogen 	Negative
Leukocytes	Negative
Nitrites		Negative
Bacteria 	Negative

## Slide 5
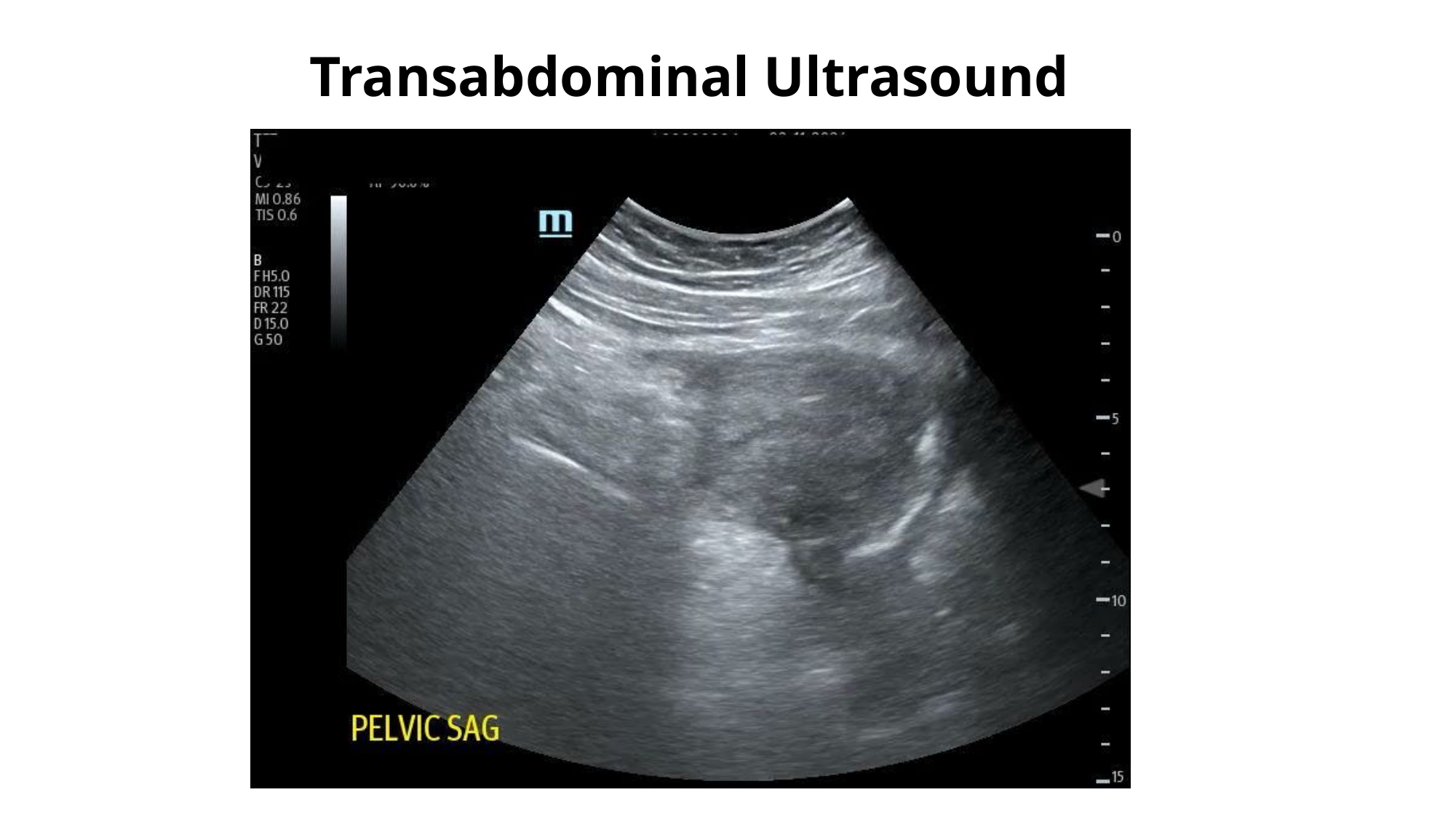

# Transabdominal Ultrasound

## Slide 6
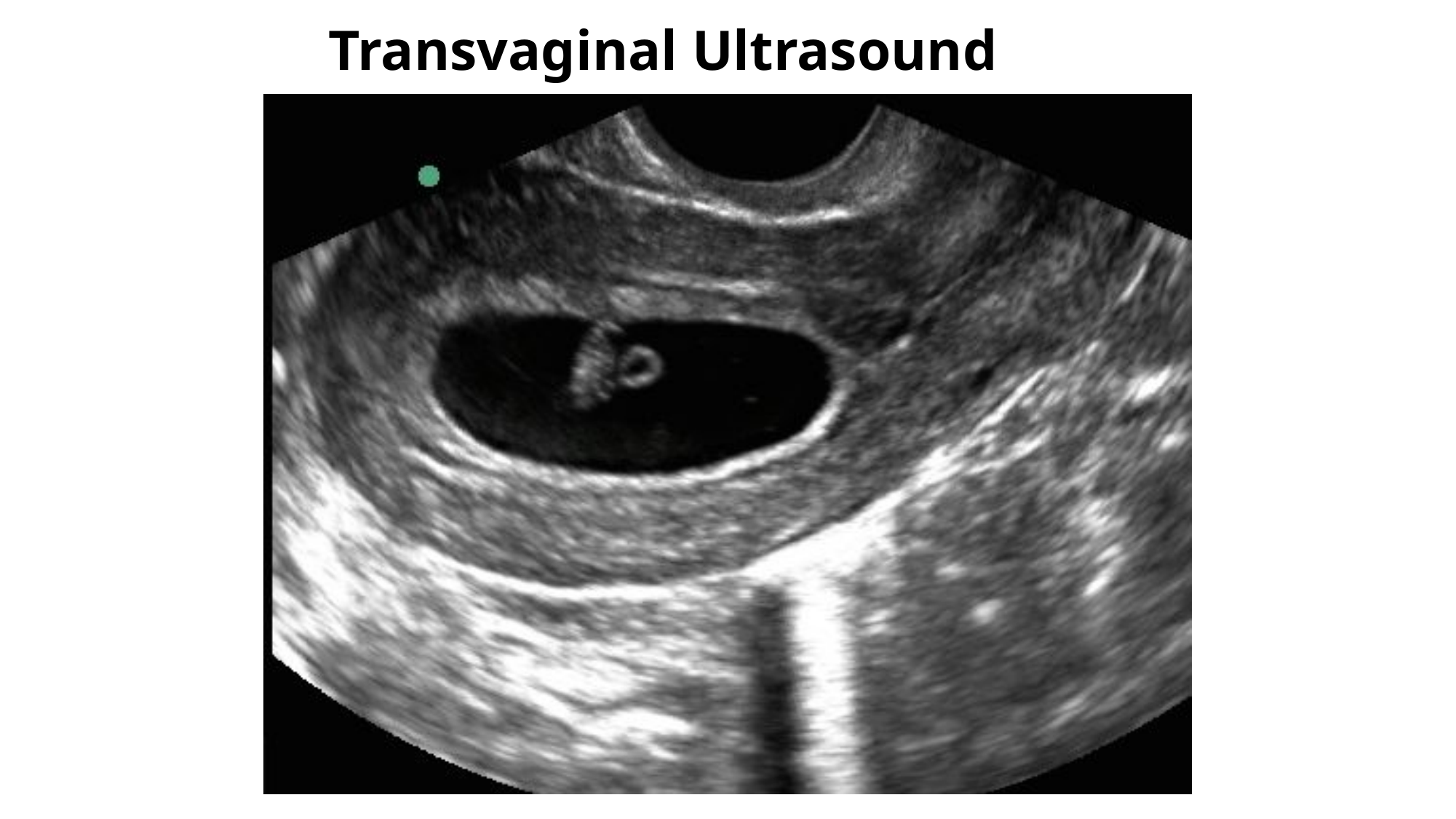

# Transvaginal Ultrasound

## Slide 7
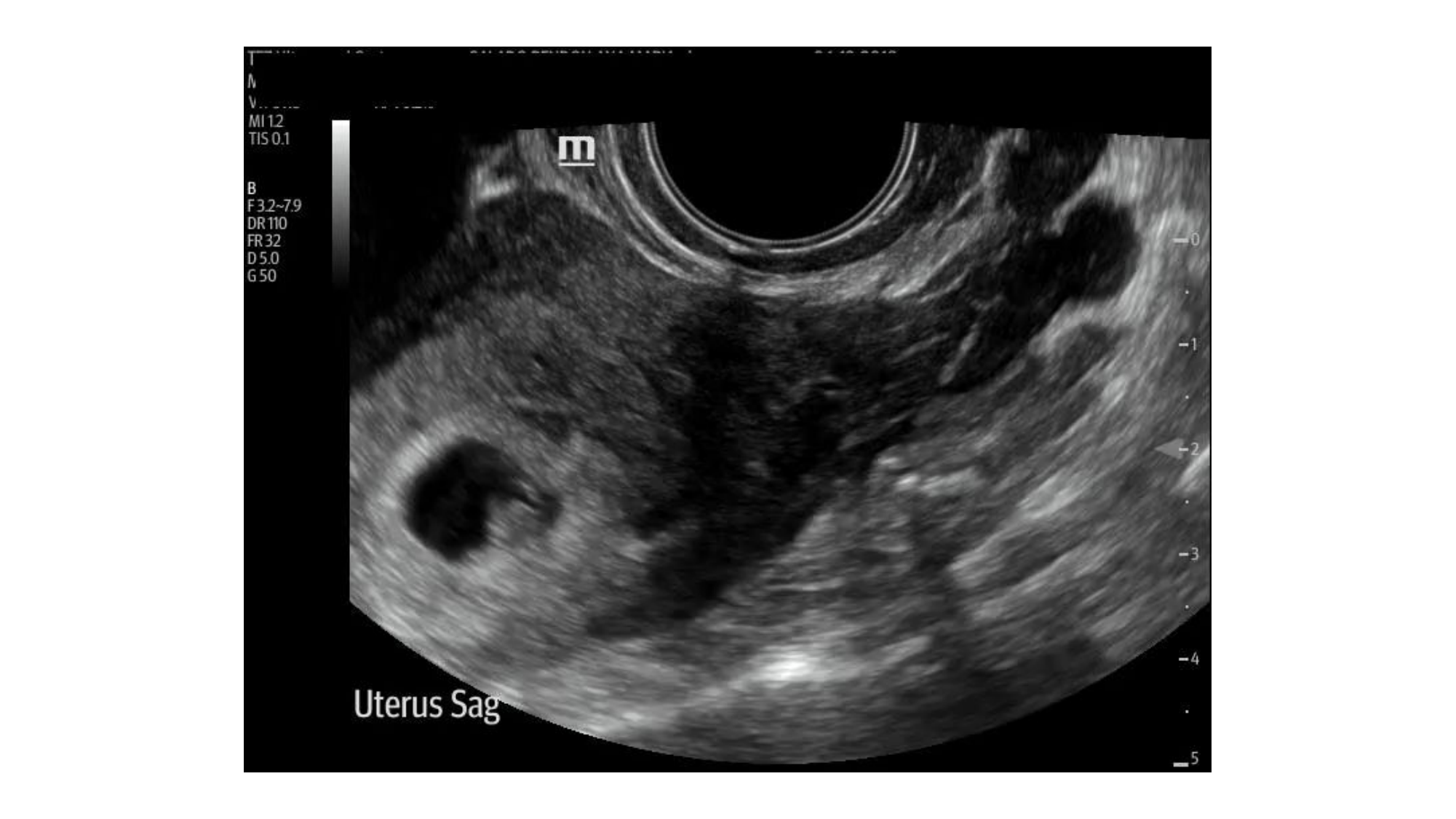

## Slide 8
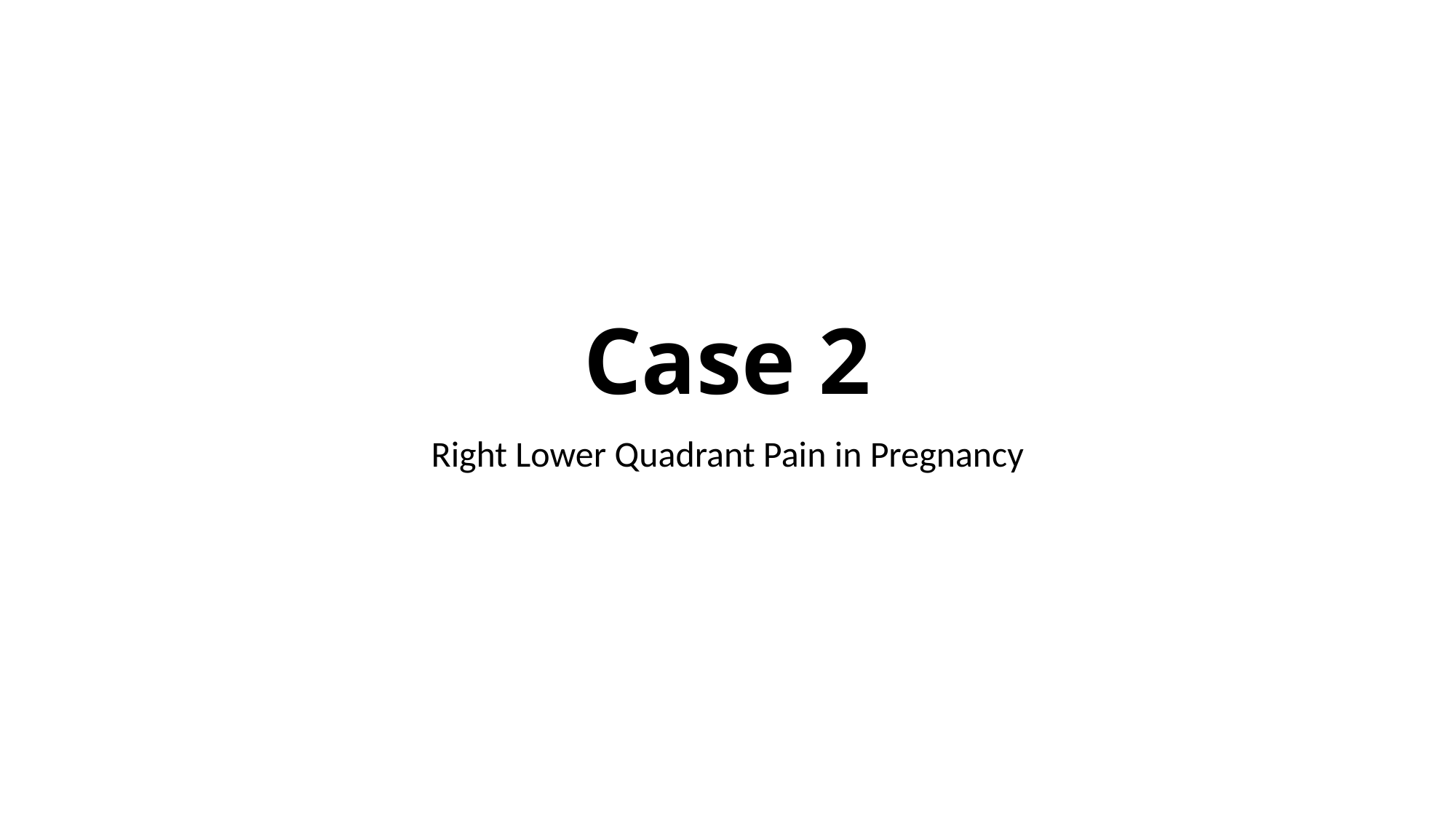

# Case 2
Right Lower Quadrant Pain in Pregnancy

## Slide 9
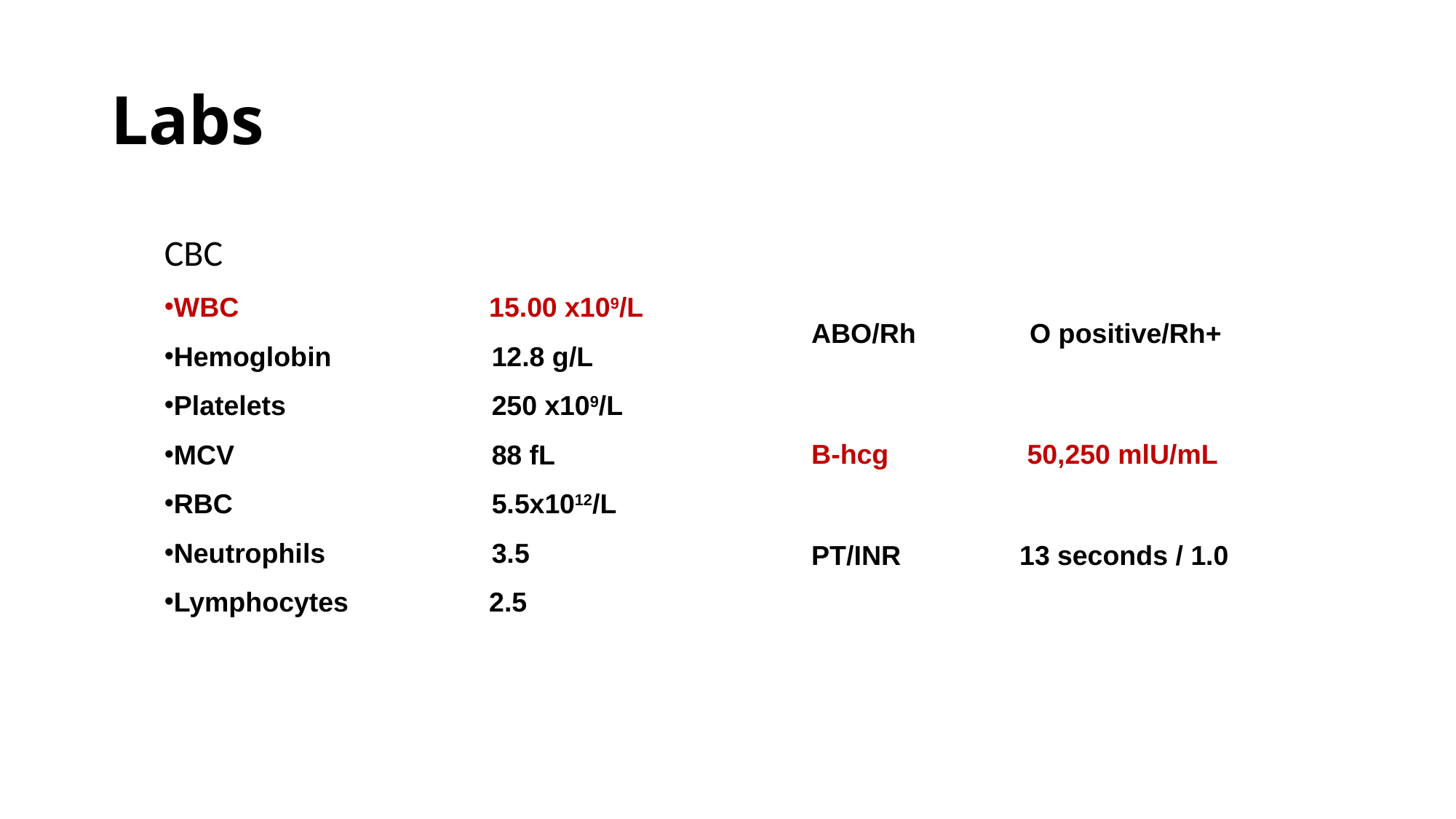

# Labs
CBC
WBC		 15.00 x109/L
Hemoglobin 		12.8 g/L
Platelets 		250 x109/L
MCV			88 fL
RBC			5.5x10^12^/L
Neutrophils 		3.5
Lymphocytes 	 2.5
ABO/Rh 	O positive/Rh+
B-hcg	 50,250 mlU/mL
PT/INR	 13 seconds / 1.0

## Slide 10
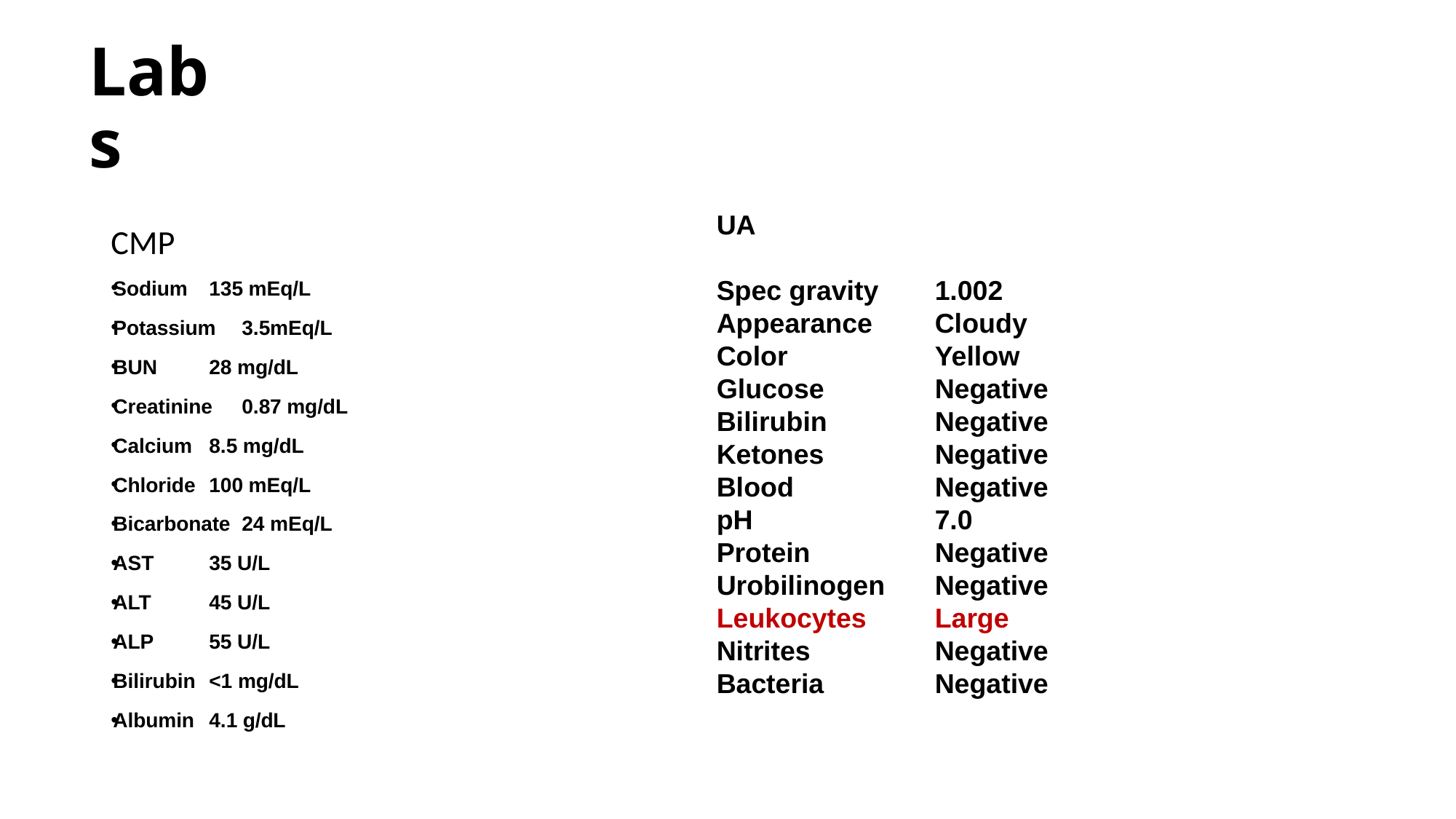

# Labs
CMP
Sodium 	135 mEq/L
Potassium 	3.5mEq/L
BUN		28 mg/dL
Creatinine	0.87 mg/dL
Calcium 	8.5 mg/dL
Chloride 	100 mEq/L
Bicarbonate	24 mEq/L
AST		35 U/L
ALT		45 U/L
ALP 		55 U/L
Bilirubin 	<1 mg/dL
Albumin 	4.1 g/dL
UA
Spec gravity 	1.002
Appearance	Cloudy
Color		Yellow
Glucose		Negative
Bilirubin 	Negative
Ketones		Negative
Blood 		Negative
pH		7.0
Protein		Negative
Urobilinogen 	Negative
Leukocytes	Large
Nitrites		Negative
Bacteria 	Negative

## Slide 11
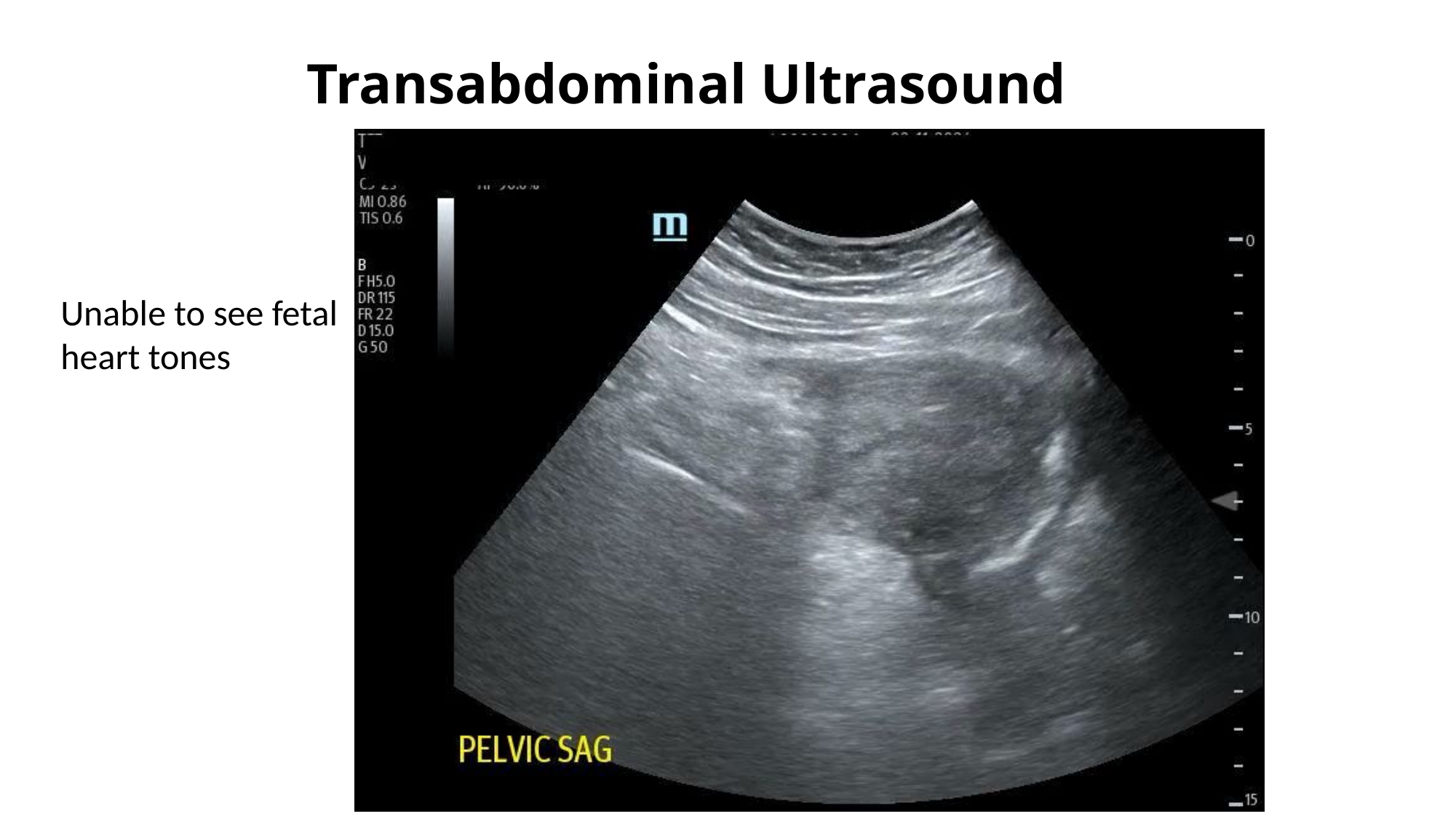

# Transabdominal Ultrasound
Unable to see fetal heart tones

## Slide 12
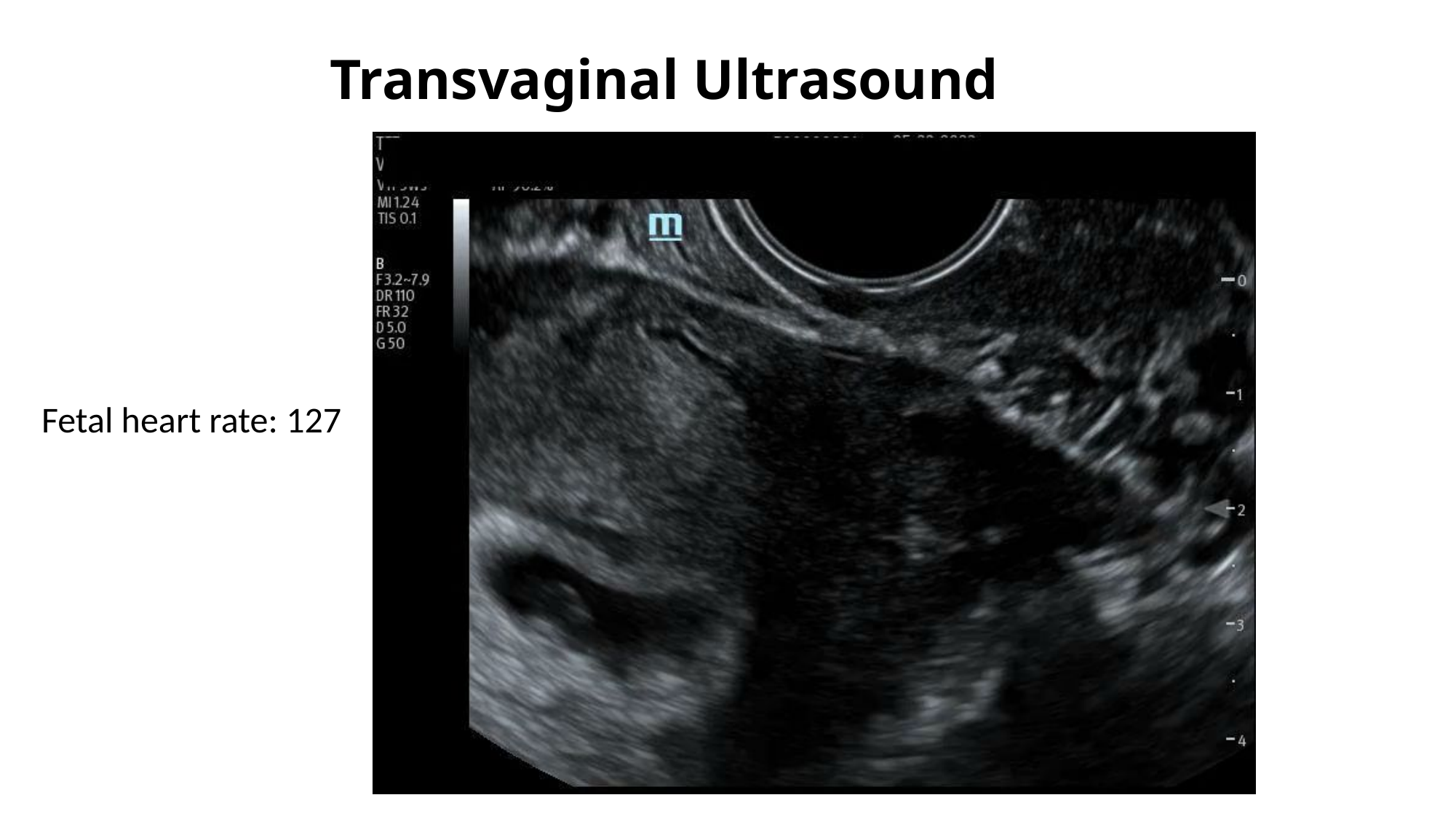

# Transvaginal Ultrasound
Fetal heart rate: 127

## Slide 13
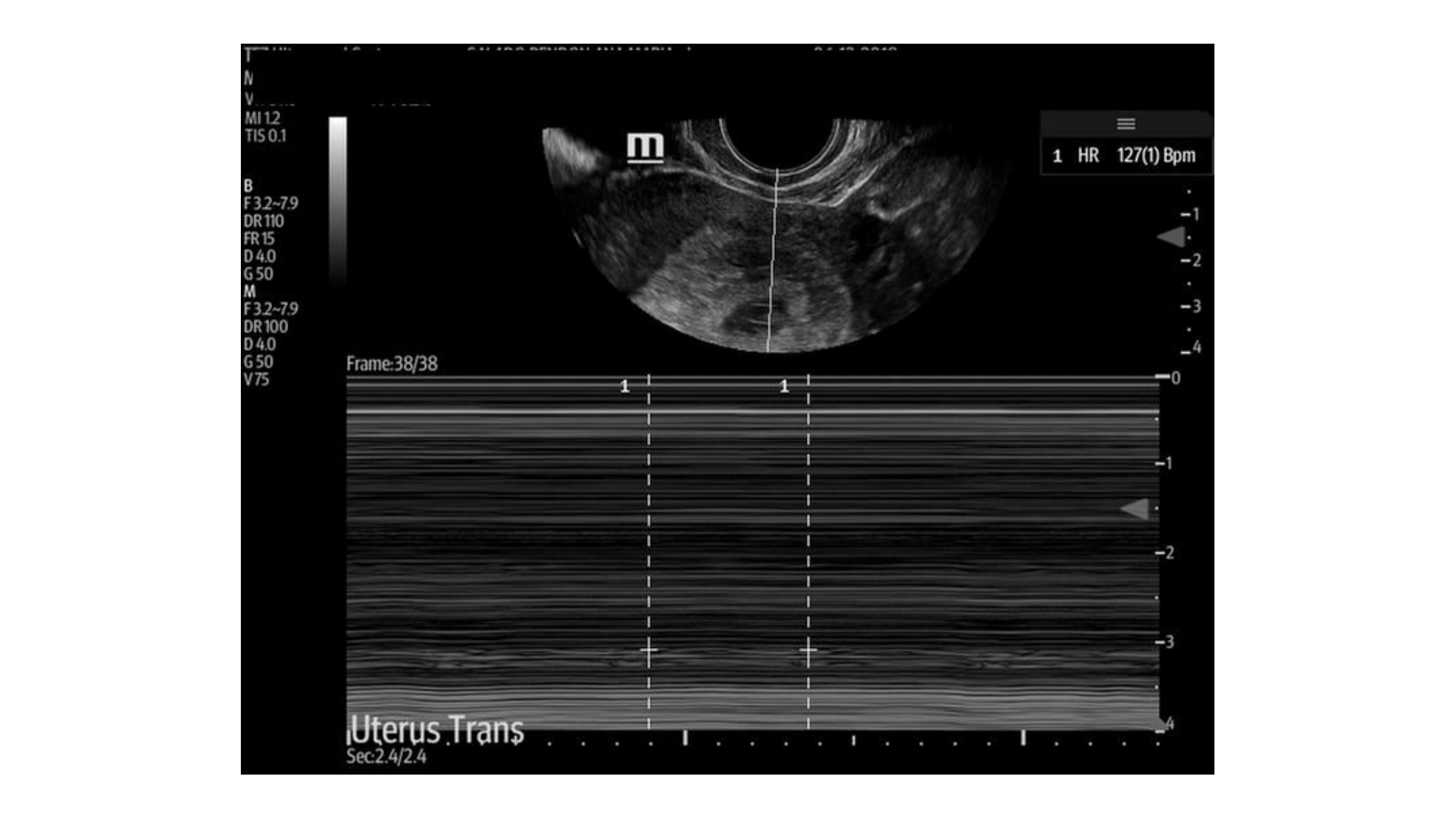

## Slide 14
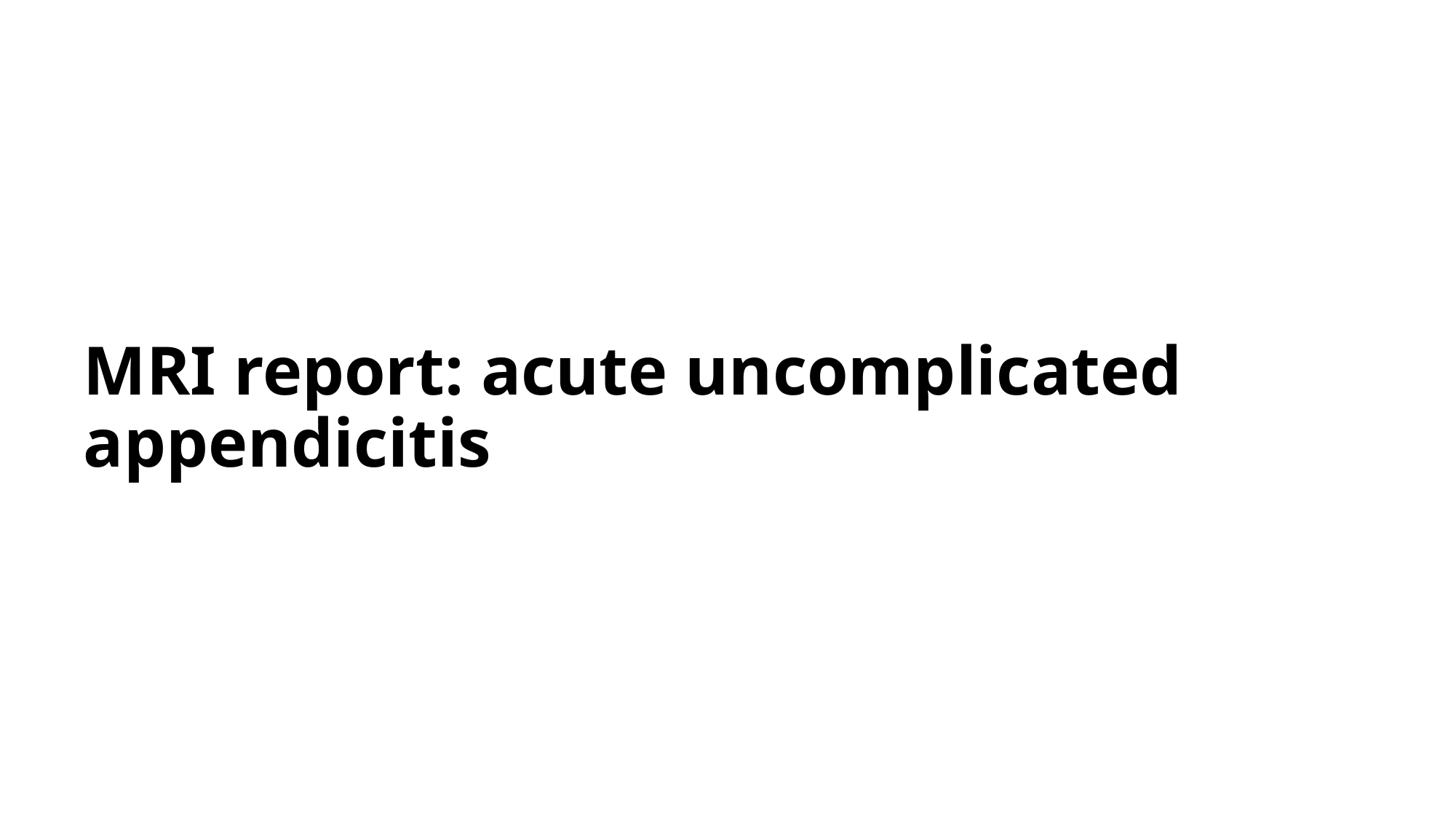

# MRI report: acute uncomplicated appendicitis

## Slide 15
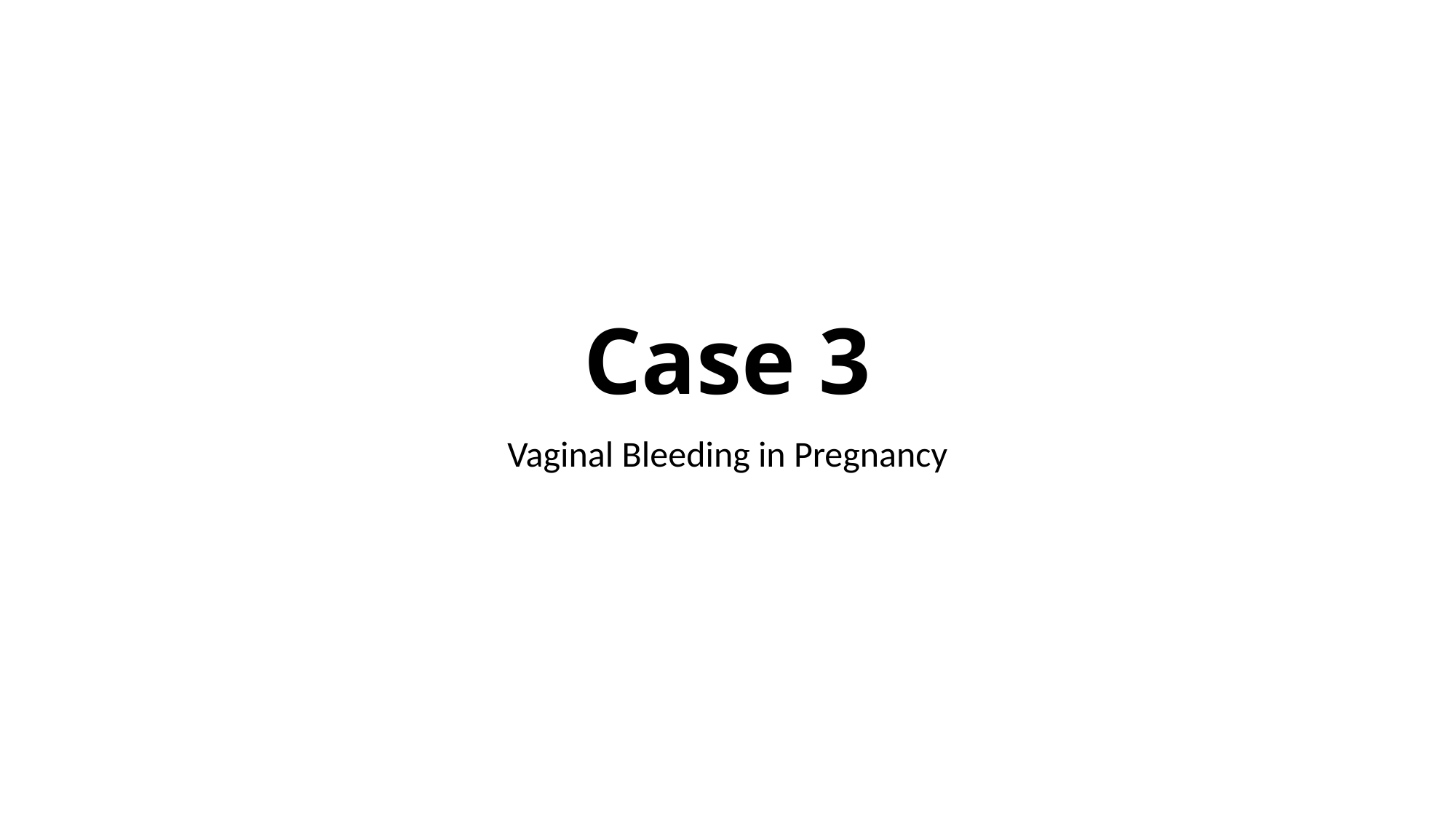

# Case 3
Vaginal Bleeding in Pregnancy

## Slide 16
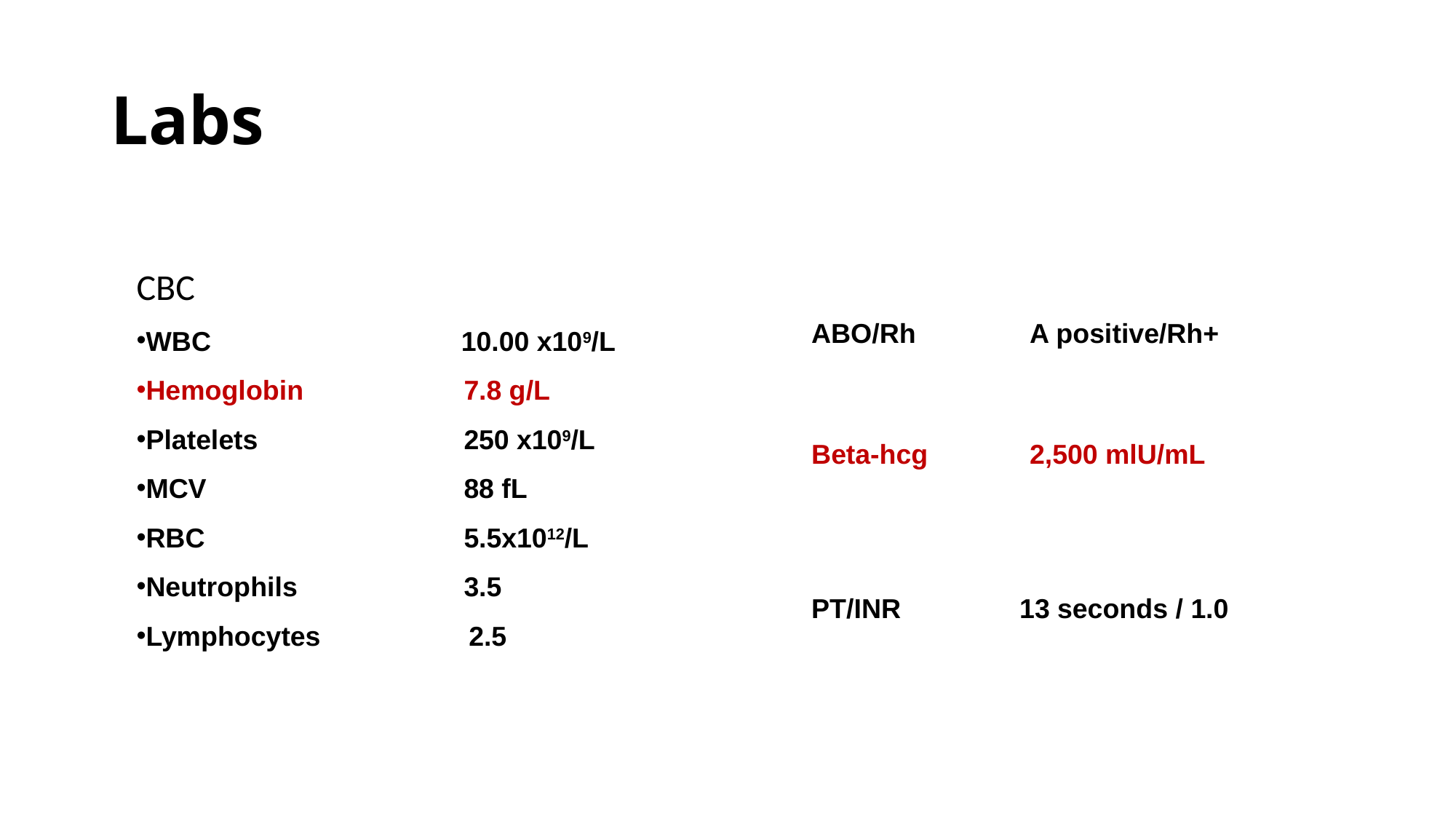

# Labs
CBC
WBC		 10.00 x109/L
Hemoglobin 		7.8 g/L
Platelets 		250 x109/L
MCV			88 fL
RBC			5.5x10^12^/L
Neutrophils 		3.5
Lymphocytes 	 2.5
ABO/Rh 	A positive/Rh+
Beta-hcg	2,500 mlU/mL
PT/INR	 13 seconds / 1.0

## Slide 17
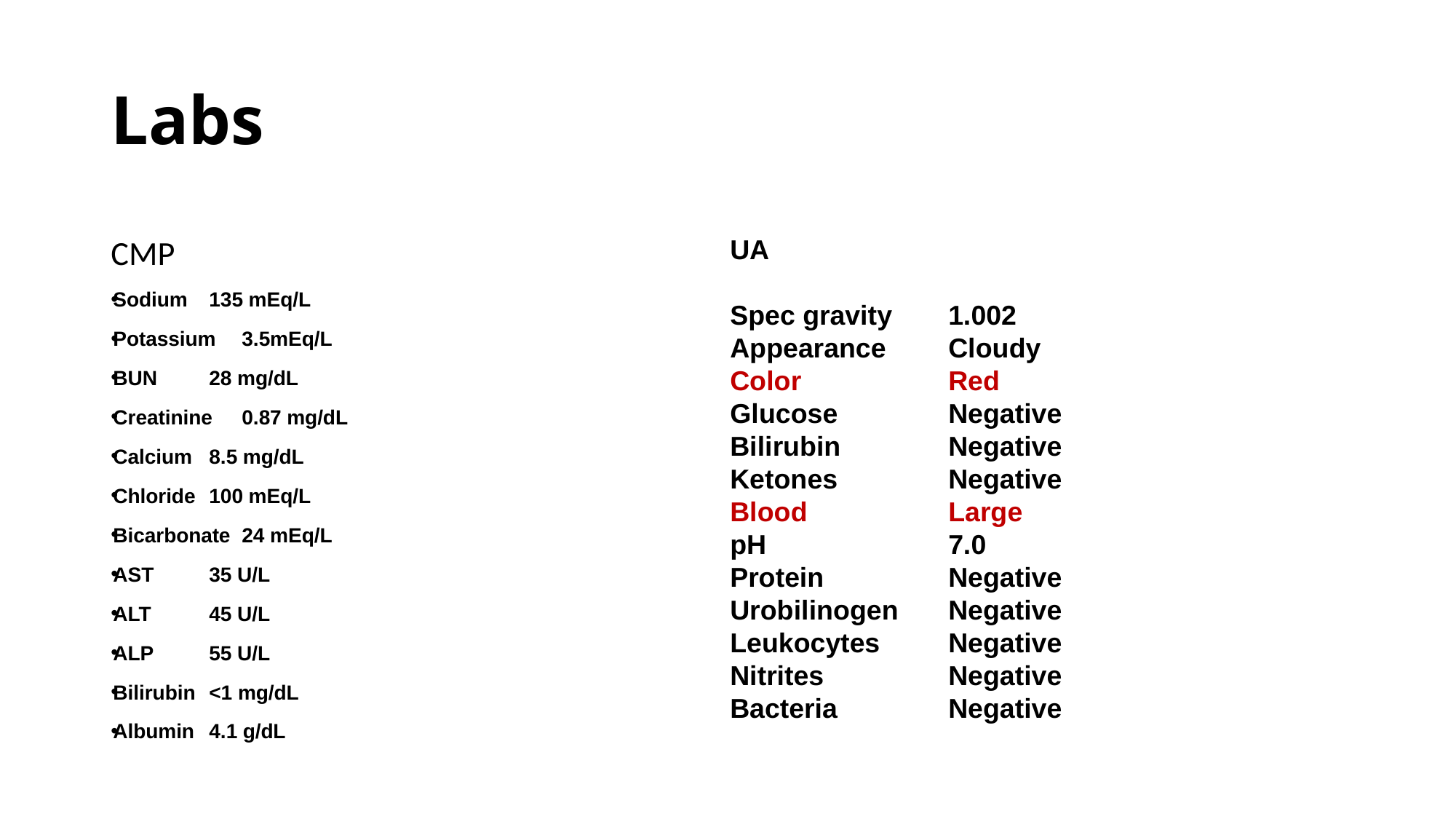

# Labs
CMP
Sodium 	135 mEq/L
Potassium 	3.5mEq/L
BUN		28 mg/dL
Creatinine	0.87 mg/dL
Calcium 	8.5 mg/dL
Chloride 	100 mEq/L
Bicarbonate	24 mEq/L
AST		35 U/L
ALT		45 U/L
ALP 		55 U/L
Bilirubin 	<1 mg/dL
Albumin 	4.1 g/dL
UA
Spec gravity 	1.002
Appearance	Cloudy
Color		Red
Glucose		Negative
Bilirubin 	Negative
Ketones		Negative
Blood 		Large
pH		7.0
Protein		Negative
Urobilinogen 	Negative
Leukocytes	Negative
Nitrites		Negative
Bacteria 	Negative

## Slide 18
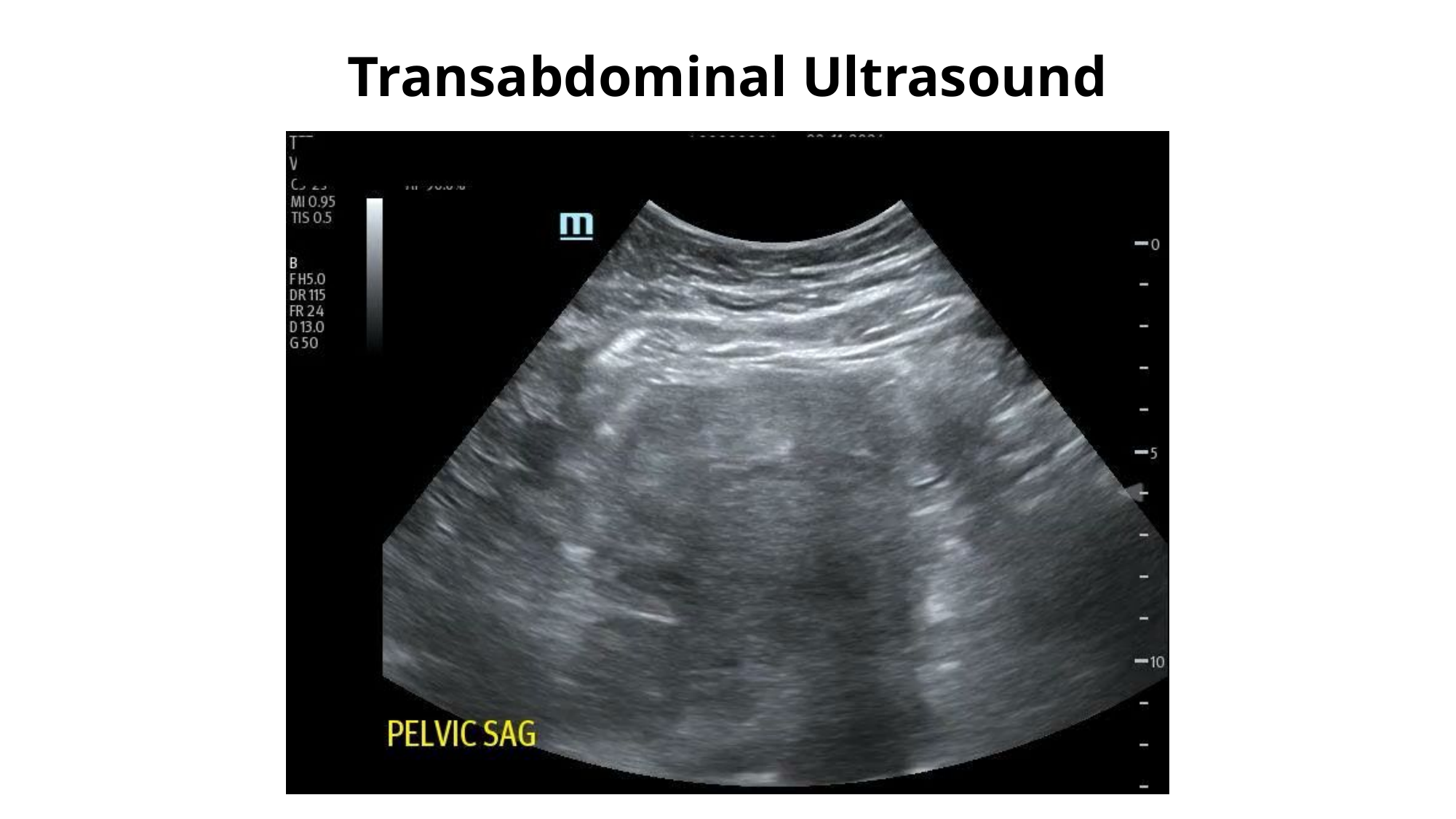

# Transabdominal Ultrasound

## Slide 19
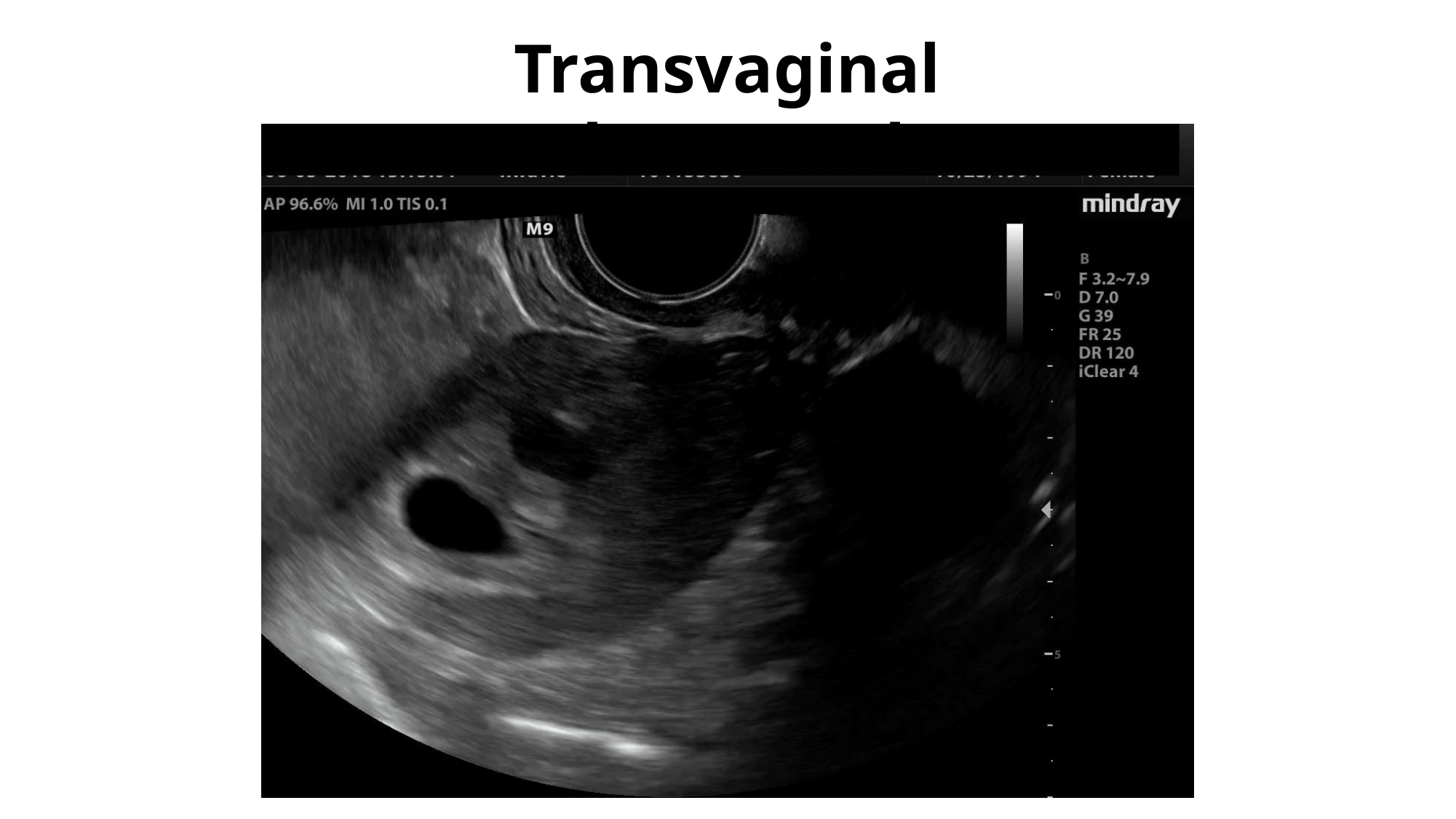

Transvaginal Ultrasound

## Slide 20
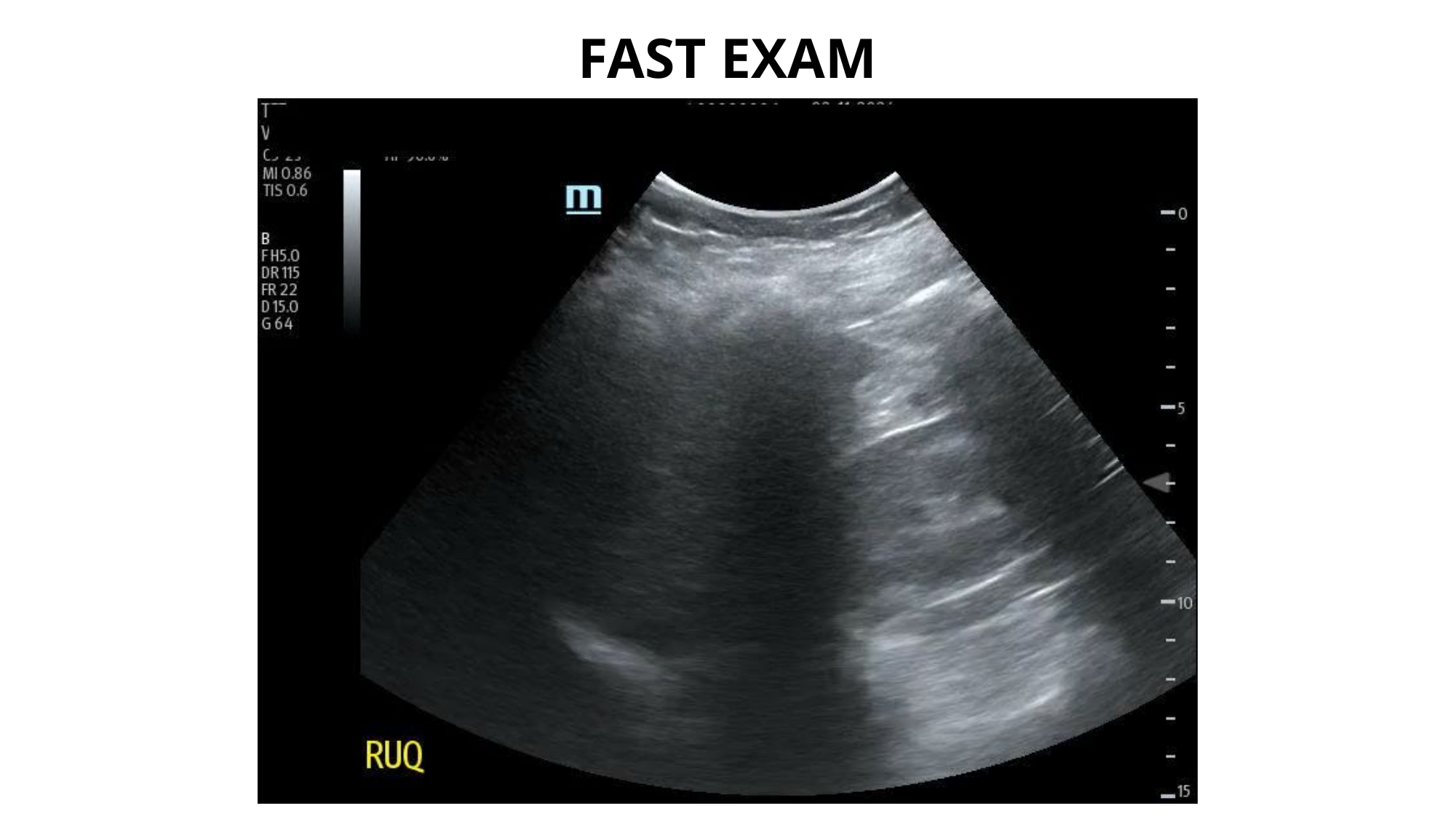

# FAST EXAM

## Slide 21
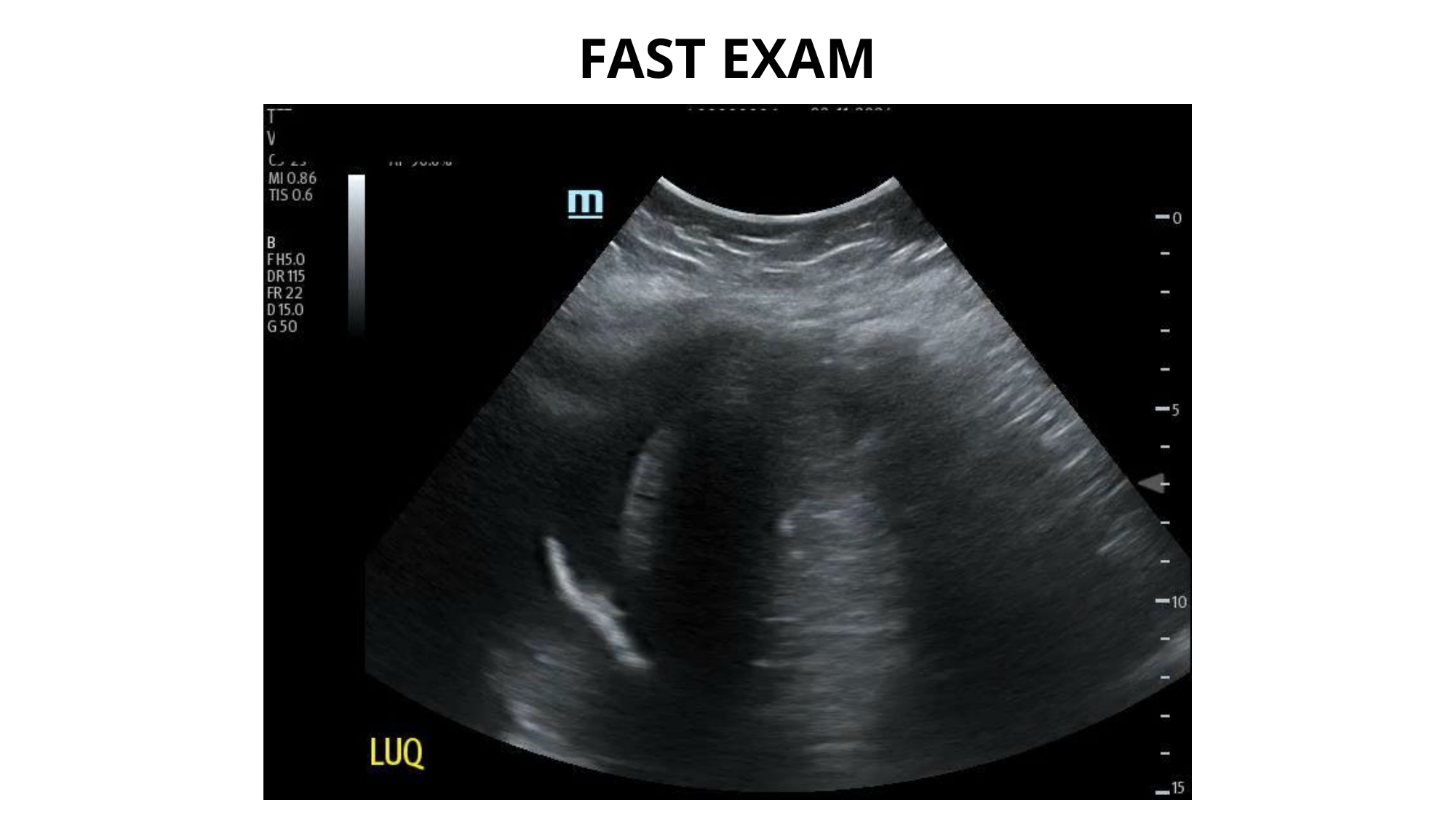

# FAST EXAM
